# Presence or absence of a novel charge-transfer complex in the base-catalyzed hydrolysis of *N*-ethylbenzamide or ethyl benzoate

**DOI:** 10.3762/bjoc.9.22

**Published:** 2013-01-29

**Authors:** Shinichi Yamabe, Wei Guan, Shigeyoshi Sakaki

**Affiliations:** 1 Fukui Institute for Fundamental Chemistry, Kyoto University, Takano-Nishihiraki-cho 34-4, Sakyo-ku, Kyoto 606-8103, Japan, phone: +81-075-711-7907

**Keywords:** basic hydrolyses, DFT calculations, ethyl benzoate, *N*-ethylbenzamide, reactive intermediates, transition states

## Abstract

Reaction paths of base-catalyzed hydrolyses of isoelectronic substrates, Ph–C(=O)–X–Et [X = O (ethyl benzoate) and X = NH (*N*-ethylbenzamide)], were traced by DFT calculations. To simulate bond interchanges accompanied by proton transfers, a cluster model of Ph–C(=O)–X–Et + OH^−^(H_2_O)_16_ was employed. For X = O, three elementary processes and for X = NH four ones were obtained. The rate-determining step of X = O is the first TS (TS1, the OH^−^ addition step), while that of X = NH is TS2. TS2 of X = NH leads to a novel Mulliken charge-transfer complex, Ph–(OH)(O=)C∙∙∙N(H_2_)–Et. The superiority or inferiority between the direct nucleophilic process or the general base-catalyzed process for TS1 was examined with the model Ph–C(=O)–X–Et + OH^−^(H_2_O)*_n_*, *n* = 3, 5, 8, 12, 16, 24 and 32. The latter process was calculated to be more favorable regardless of the number (*n*, except *n* = 3) of water molecules. The counter ion Na^+^ works unfavorably on the ester hydrolysis, particularly on TS1. A minimal model of TS1 was proposed and was found to be insensitive to *n*.

## Introduction

Basic hydrolyses of esters and amides have been extensively studied experimentally [1]. Use of dilute alkali is the usual way of hydrolyzing esters, and the reaction is called saponification. The base-catalyzed hydrolysis of amides is an important model for the enzymatic cleavage of peptide bonds [2–3]. The base-promoted hydrolyses of carboxylic esters and amides accompanying the ^18^O exchange have been investigated to characterize reversibly formed intermediates [4–16].

Through the analysis of heavy-atom isotope effects, the rate-determining step of the alkaline hydrolysis of methyl benzoate (Ph–C(=O)–OMe) was shown to be the formation of the anionic tetrahedral intermediate by O'Leary and Marlier [17]. Marlier suggested that the attacking nucleophile in aqueous solution is water with OH^−^ assistance in the hydrolysis of methyl formate (HCOOCH_3_) [18]. This suggestion is in sharp contrast to the traditional B_ac_2 mechanism [19]. In this mechanism, the tetrahedral intermediate is formed by direct nucleophilic collisions between hydroxide ions and ester molecules. Marlier's suggestion was supported by a kinetic study of the saponification of ethyl acetate (CH_3_COOC_2_H_5_) [20].

**Scheme 1:**
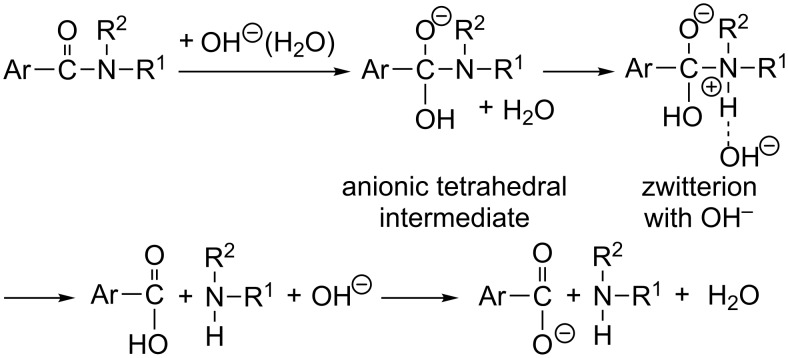
A scheme of the base-catalyzed amide hydrolysis involving a zwitterion suggested by analyses of solvent kinetic isotope effects [22].

For the base-catalyzed amide hydrolysis, Brown and co-workers made extensive studies of the carbonyl ^18^O exchange and D_2_O solvent kinetic isotope effects [21–25]. They suggested intervention of a pair of a zwitterion and OH^−^ as well as that of the anionic tetrahedral intermediate (Scheme 1).

Although many theoretical studies of the basic amide hydrolysis have been reported so far [26–37], the presence or absence of the zwitterion has not been scrutinized.

As for zwitterions of amino acids and peptides, the effect of the solvent on the structure and various spectra has been examined carefully [38–46]. Explicit solvent models were reported to be necessary, and it was shown that the use of continuum solvent models is not adequate. For instance, Degtyarenko and co-workers demonstrated that 20 water molecules are needed to completely solvate the L-alanine zwitterion [46]. The average number of water molecules in the first hydration shell of an alanine molecule was found to be seven [44]. Thus, more than seven water molecules would be required to examine the reaction paths of hydrolyses reliably. However, less than six water molecules are included in the precedent computational studies [26–37]. As stated in [20], "an appropriate mechanistic picture for the system (saponification) must take into account the solvent molecules that should be included in the minimal TS structure".

**Scheme 2:**
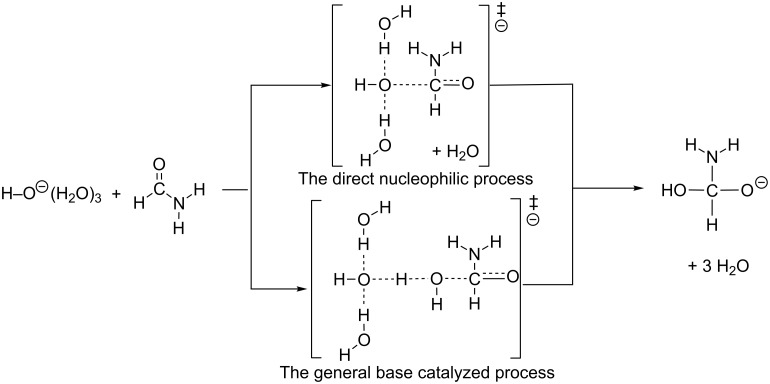
Two processes suggested by a proton-inventory NMR study [25].

The mechanisms of the well-known two base-catalyzed hydrolyses are still unclear in the following points:

1. The rate-determining step of the ester hydrolysis was suggested to be the nucleophilic OH^−^ addition to the carbonyl carbon according to the kinetic result of the heavy-atom isotope effect [17]. On the other hand, in the hydrolyses of a series of toluamides (Me–C_6_H_4_–C(=O)–N(R^1^)(R^2^)), the rate-determining steps were reported to vary from the OH^−^ attack to breakdown of the anionic tetrahedral intermediate [22]. It seems that the rate-determining step of the amide reaction is not as definite as that of the ester one.

2. The number of elementary processes in both hydrolyses is yet unknown. Is the zwitterion shown in Scheme 1 also present in the ester reaction?

3. In [25], the direct nucleophilic process was suggested to be more favorable than the general base-catalyzed process for the hydrolysis of formamide (Scheme 2). This suggestion is in contrast to Marlier's one mentioned above for the hydrolysis of methyl formate [18]. Does the amide take a different OH^−^ addition process from that of the ester? Is the controversial OH^−^ addition transition state affected by the number of water molecules adopted in calculations?

4. The base-catalyzed hydrolysis is conducted usually by the use of the NaOH (i.e., 0.01 to 1.0 mol/L aqueous) solution. Then, the role of the counter ion Na^+^ on the reaction paths and energies needs to be investigated.

5. Is "the minimal TS structure" [20] predictable in the framework of the molecular model prior to any calculations?

**Scheme 3:**
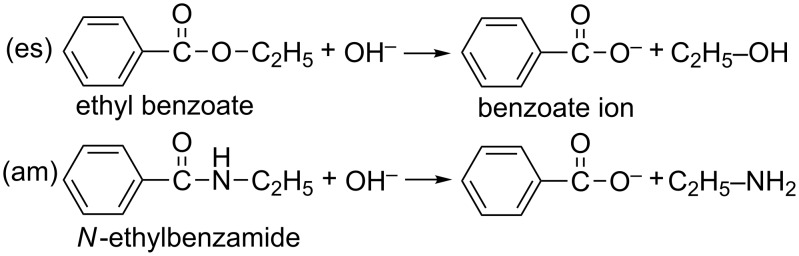
Hydrolysis of the ester (saponification) and the amide adopted in this work. These are assigned as (es) and (am), respectively.

In this work, DFT calculations were carried out to shed light on the five points above, 1–5. As isoelectronic substrates, ethyl benzoate and *N*-ethylbenzamide were employed, of which the reactions are shown in Scheme 3.

The former (ester) reaction has been studied well and its activation energy was reported to be 14.6 kcal/mol [47]. The latter (amide) analogous one (*N*,*N*-dimethyltoluamide, Me–C_6_H_4_–C(C=O)–NMe_2_) was investigated and the experimental activation free energy was reported to be 27.1 kcal/mol [22]. The hydrolysis of the phenyl-group-containing substrate was studied computationally in a reaction between *N*-methylbenzamide and OH^−^ [37]. However, only one water molecule was contained in the reaction system.

**Scheme 4:**
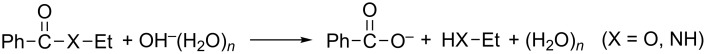
A reaction model including the water cluster.

In the present study, the number of water molecules (*n*) is changed systematically in Scheme 4 to address the latter part of point 3.

## Method of calculations

The reacting systems were investigated by density functional theory calculations. The B3LYP [48–49] method was used to trace the reaction path. B3LYP seems to be a suitable method, which includes the electron correlation effect to some extent. The basis set employed was 6-31(+)G(d), where diffuse functions are added to oxygen and nitrogen atoms. Since the present systems are large (for the largest stoichiometry C_9_H_76_NO_34_^(−1)^ of *n* = 32 (amide), 952 basis functions of 6-31(+)G(d) in the geometry optimizations), calculations with higher-level basis sets than 6-31(+)G(d) are too difficult.

As for the key step, i.e., the OH^−^ addition process, TS geometries of *n* = 16 were re-optimized with B3LYP/6-311+G(d,p), B3PW91 [50]/6-31(+)G(d), B3PW91/6-31(+)G(d) SCRF (self-consistent reaction field) = PCM [51–53], B3PW91/6-311+G(d,p) and M06-2X [54]/6-31(+)G(d). These re-optimizations are needed to check whether the obtained TS structures are insensitive to the adopted method or not in relation to the former part of point 3.

Transition states (TSs) were sought first by partial optimizations at bond-interchange regions. Second, by the use of Hessian matrices, TS geometries were optimized. They were characterized by vibrational analyses, which checked whether the obtained geometries have single imaginary frequencies (ν^≠^s). From TSs, reaction paths were traced by the intrinsic reaction coordinate (IRC) method [55–56] to obtain the energy-minimum geometries. In order to check the character of the HO^−^ addition TS, classical trajectory calculations using the atom-centered density-matrix-propagation molecular dynamics (ADMP) model [57–59] were also conducted.

Relative energies (Δ*E*s) and Gibbs free ones (Δ*G*s) were obtained by single-point calculations of RB3LYP/6-311++G(d,p) {SCRF = PCM, solvent = water} on the RB3LYP/6-31(+)G(d) geometries and their ZPVE and thermal corrections, respectively.

All the calculations were carried out by using the GAUSSIAN 09 [60] program package. The computations were performed at the Research Center for Computational Science, Okazaki, Japan.

## Results and Discussion

### Consideration of minimal and extended TS structures

**Figure 1:**
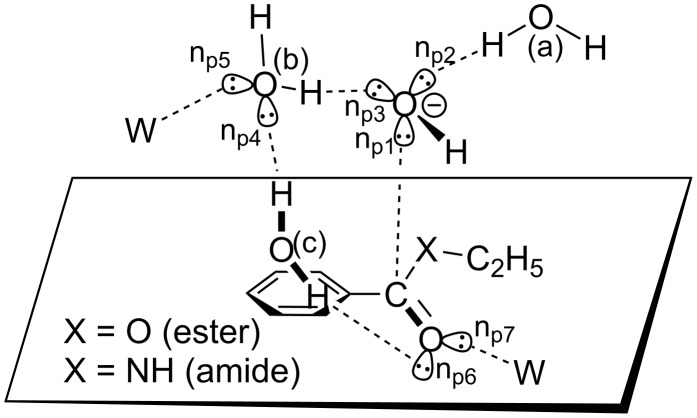
A minimal model of the OH^−^ nucleophilic addition to the substrate, Ph–C(=O)–X–Et. Three ((a), (b) and (c), *n* = 3) water molecules participate in the main hydrogen bonds to stabilize the TS structure. W stands for the water molecule as an outer catalyst. n_p_ denotes the lone-pair orbitals on the oxygen atom.

According to the requirement of "an appropriate mechanistic picture of the minimal TS structure" [20], a model of the OH^−^ addition to the carbonyl carbon was made and is shown in Figure 1. At the OH^−^ addition, one lone-pair orbital (n_p1_) of OH^−^ is directed to the carbonyl carbon. The other two ones (n_p2_ and n_p3_) should be linked to two water molecules, (a) and (b). n_p4_ and n_p5_ of the water (b) become anionic through the OH^−^ → H_2_O(b) charge transfer (CT). Then, a water (c) may be linked to n_p4_ of H_2_O(b) and n_p6_ of the carbonyl oxygen in the bridged form. n_p6_ and n_p7_ of the carbonyl oxygen becomes anionic as the OH^−^ addition proceeds. Two anionic n_p5_ and n_p7_ are linked with the outer two water molecules (W's). The assumed picture in Figure 1 is in line with the experimental suggestion that five water molecules participate in the reaction center [25].

**Figure 2:**
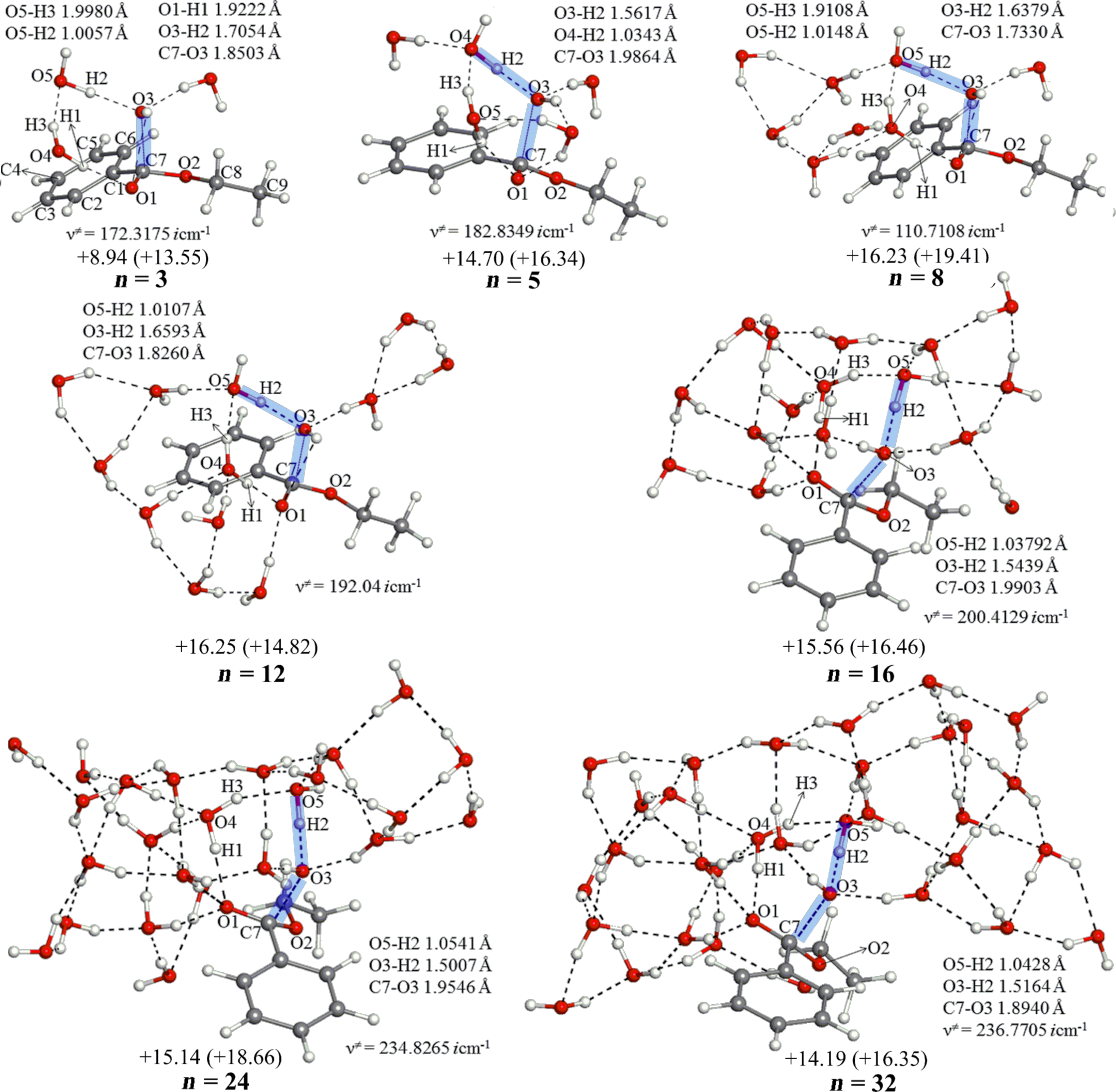
Geometries and B3LYP/6-31(+)G(d) activation energies of TS1(es) in the reaction between ethyl benzoate and OH^−^(H_2_O)*_n_*. Activation and activation free energies in kilocalories per mole (1 kcal = 4.184 kJ) are shown without and with parentheses, respectively. For instance, *E*_a_ = + 8.94 kcal/mol and Δ*G*^≠^ = + 13.55 kcal/mol for *n* = 3. Cartesian coordinates of all the TS geometries are shown in VII.a (Supporting Information File 1).

The assumption in Figure 1 was examined by varying the number of water molecules in Scheme 4 at the OH^−^ addition transition state (TS1). Figure 2 shows geometries of TS1(es) for *n* = 3, 5, 8, 12, 16, 24 and 32 in the ester hydrolysis. Hereafter, the ester reaction is shown by (es) and the amide one is by (am). They exhibit that the skeletal part of *n* = 3 (without two W's in Figure 1) is retained in all the TS1(es) geometries. In addition, the *n* = 5 geometry is close to that drawn qualitatively in Figure 1. Here, H3–O5–H1 is the bridged H_2_O (c) in Figure 1.

**Figure 3:**
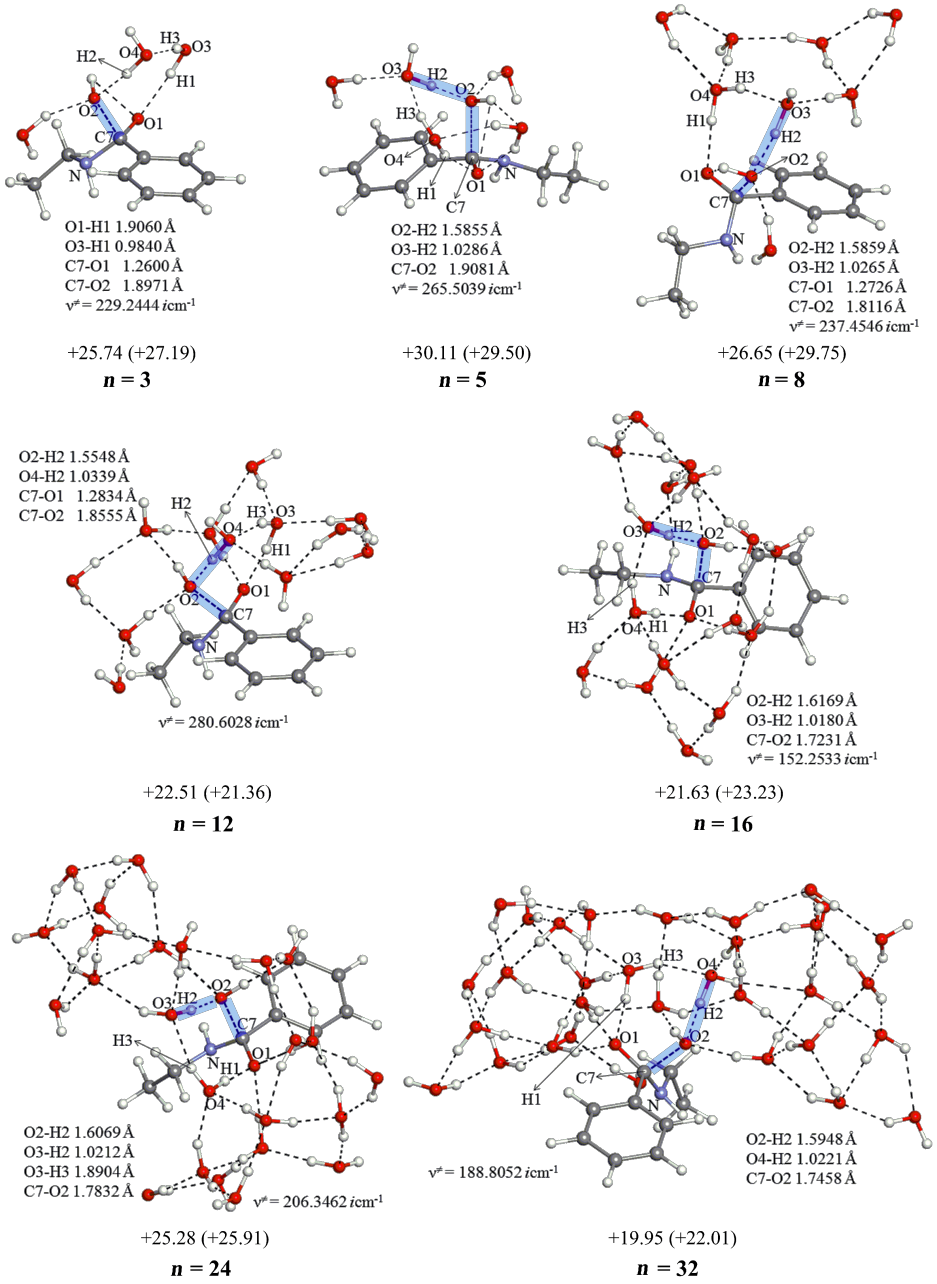
Geometries and activation energies of TS1(am) in the reaction between *N*-ethylbenzamide and OH^-^(H_2_O)*_n_*. XYZ coordinates in VII.b (Supporting Information File 1).

Figure 3 shows those in the amide hydrolysis. Again, the *n* = 3 central parts are retained in TS1(am) geometries of *n* = 5, 8, 12, 16, 24 and 32.

From all TS1 geometries (except that of *n* = 3), IRC calculations were carried out to judge which is more likely, the direct nucleophilic process or the general base-catalyzed one in Scheme 2. All the "reverse" geometries (i.e., those of the reactant-like complex) are found to have the OH^−^ at the H_2_O(b) position (see Figure 1). Thus, the general base-catalyzed process was calculated to be more likely regardless of the number of water molecules. The judgment is also checked by the other methods than B3LYP/6-31(+)G(d). They are B3LYP/6-311+G(d,p), B3PW91/6-31(+)G(d), B3PW91/6-31(+)G(d) SCRF = PCM, B3PW91/6-311+G(d,p) and M06-2X/6-31(+)G(d). By their TS and IRC calculations of the *n* = 16 system, the general base-catalyzed process was confirmed. Key distances in TS1(es) and TS1(am) are shown in Tables S1 and S2 (Supporting Information File 1), respectively.

The trajectory calculation may give a different result, if the potential surface at the OH^−^ addition step is shallow. In order to check this point, the ADMP molecular dynamics calculation was made starting from TS1(es) of the ester *n* = 16. After 800 femtoseconds, the resultant geometry is shown in the right of Figure S1. The geometry is similar to that of the reactant-like complex obtained in the IRC calculation. Again, the general base-catalyzed process was confirmed.

In view of geometries and the calculated activation energies, the *n* = 16 model was selected to trace elementary processes, in a balance between reliability and computational difficulty.

### Reaction paths in the ester hydrolysis

**Figure 4:**
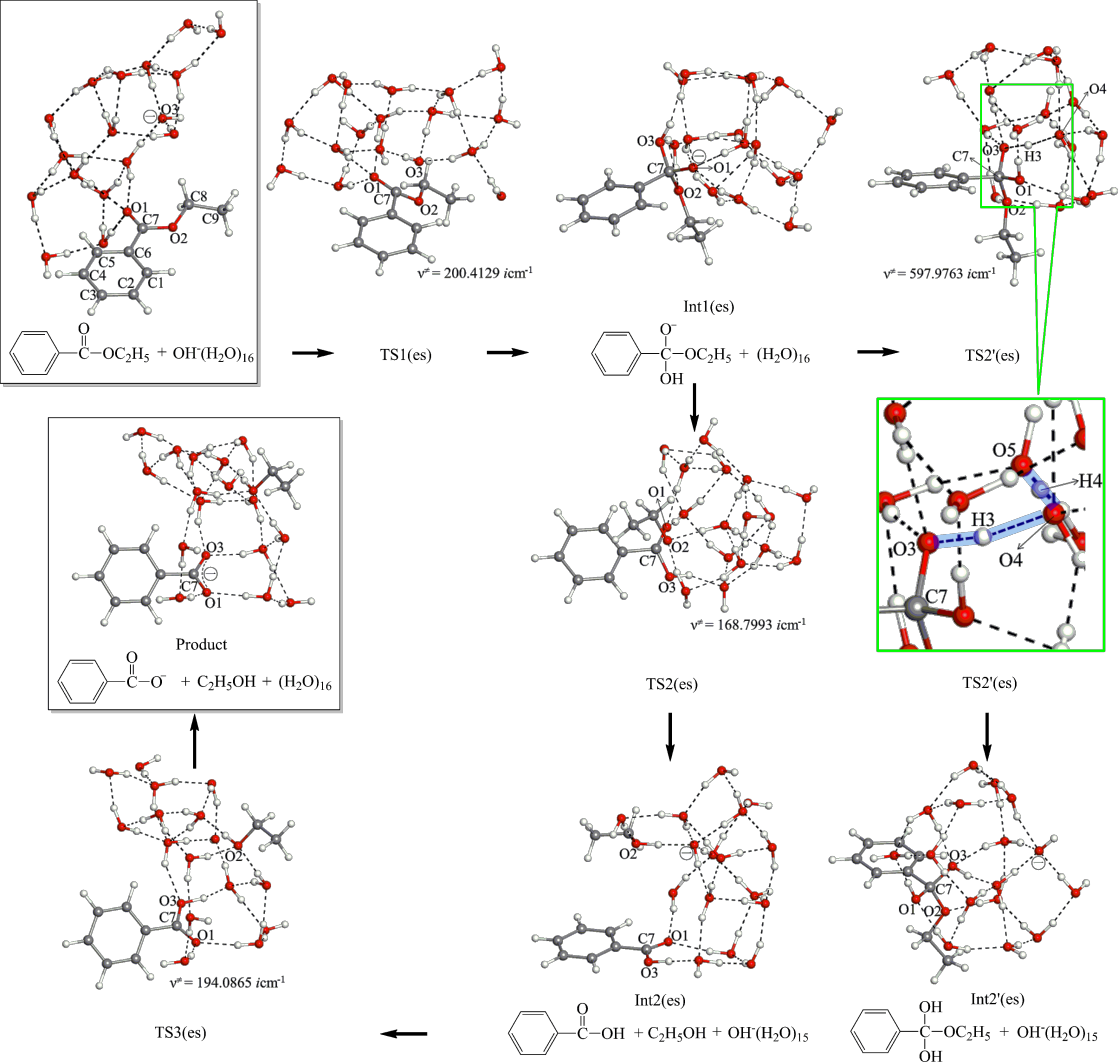
Reaction paths of the ester *n* = 16 hydrolysis, starting from the boxed reactant-like complex toward the boxed product. To TS2'(es), the magnified cut figure is attached. XYZ coordinates in VII.c (Supporting Information File 1).

Figure 4 exhibits geometric changes in the *n* = 16 ester hydrolysis. Starting from the reactant-like complex, OH^−^ adds to the carbonyl carbon at TS1(es). After TS1(es), the expected anionic tetrahedral intermediate, Int1(es), is formed. At the intermediate, the alkoxide oxygen O(3) is the most anionic.

**Scheme 5:**
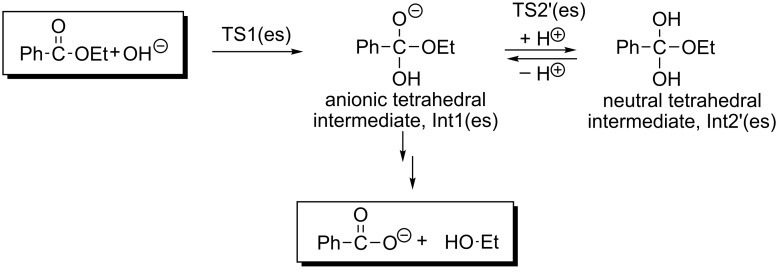
A possibility that the neutral tetrahedral intermediate is the stock of concentrations for the irreversible hydrolysis route.

A proton-attach TS, TS2'(es), was obtained. After TS2'(es), a neutral tetrahedral intermediate Int2'(es), is formed. If this intermediate is very stable, it should be in equilibrium with the reactant-like complex. Then, the hydrolysis occurs as a nonequilibrium route according to Le Chatelier's principle (Scheme 5).

The scheme will be evaluated by comparing the calculated energies. As the alternative route to Int1(es), TS2(es) was obtained. At TS2(es), C∙∙∙O cleavage and the proton transfer occur simultaneously. This process is different to that thought so far (C∙∙∙O scission only forming C_2_H_5_O^−^). Formation of the unstable ethoxide ion is avoided by the concomitant proton transfer. After TS2(es), the {Ph–COOH + Et–OH + OH^−^(H_2_O)_15_} intermediate (Int2(es)) is afforded. The combination of Ph–COOH and OH^−^ leads to TS3(es), where the double proton transfer is involved. After TS3(es), the product of {Ph–COO^−^ + Et–OH + (H_2_O)_16_} is generated.

Figure 4 demonstrates that the hydrolysis of ethyl benzoate has three elementary processes (except TS2'(es)). The ethoxide-ion intermediate and the zwitterion shown in Scheme 1 were not found during the reaction. It is noteworthy that four TSs (TS1(es), TS2'(es), TS2(es) and TS3(es)) contain proton transfers. For instance, the reaction center of TS2'(es) may be described as O5----H4----O4----H3----O3. Lines ---- indicate the intermediate character of O–H covalent and O∙∙∙H hydrogen bonds. Thus, proton transfers were found to regulate, significantly, the reaction paths of the ester hydrolysis.

### Reaction paths in the amide hydrolysis

**Figure 5:**
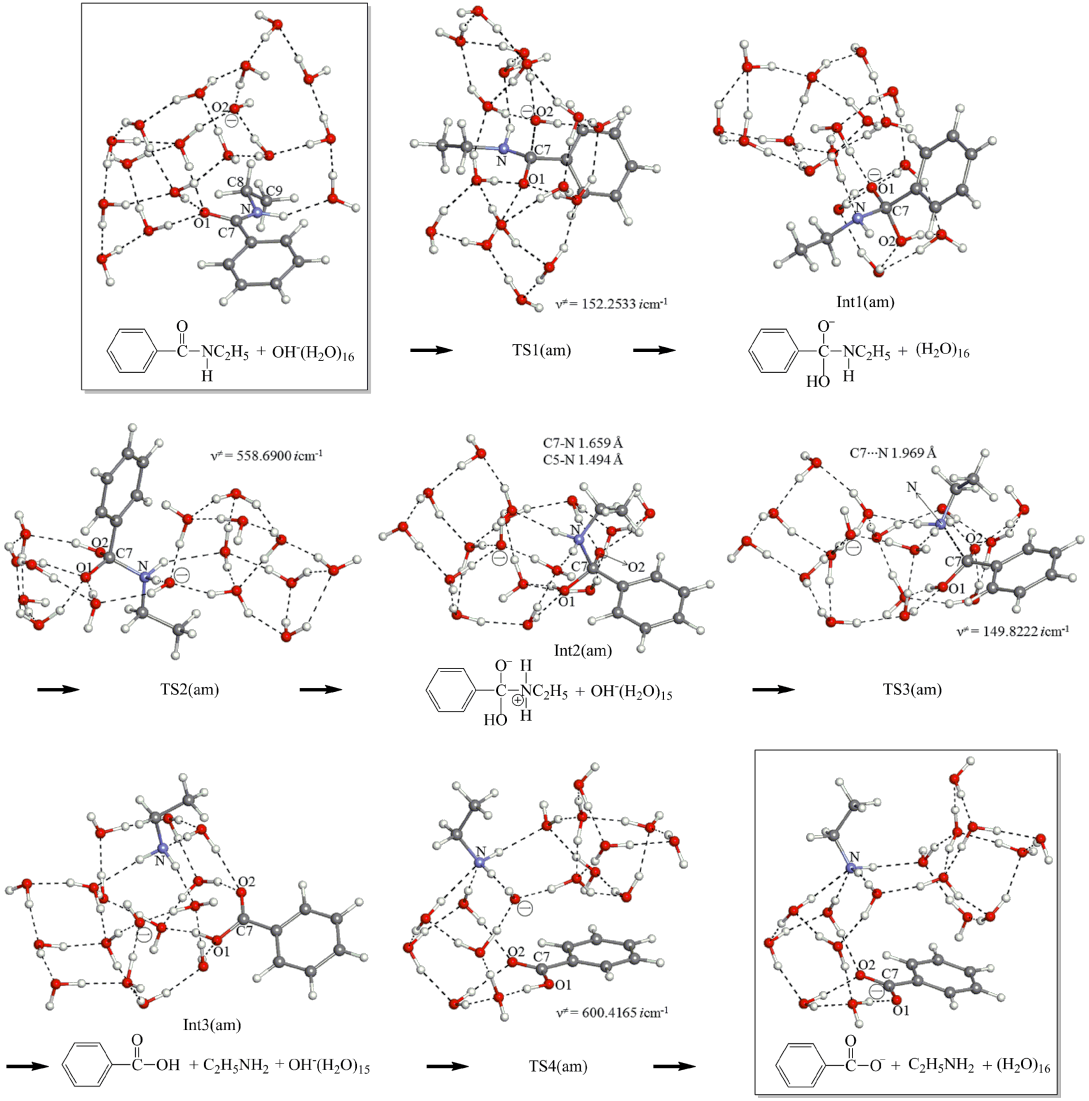
Reaction paths of the amide *n* = 16 hydrolysis. XYZ coordinates in VII.d (Supporting Information File 1).

Figure 5 exhibits geometric changes in the *n* = 16 amide hydrolysis. The first step is the OH^−^ addition (TS1(am)) leading to the anionic tetrahedral intermediate, Int1(am). From the anion, a path similar to TS2(es) in Figure 4 was sought. However, a different TS, namely TS2(am), was obtained. At TS2(am), only the double proton transfer takes place, where the C(7)–N bond is retained. A "zwitterion ion" intermediate Int2(am) suggested in Scheme 1 was derived. This is the first case where the intermediate is calculated. However, the geometry is regarded as a Mulliken CT complex rather than a zwitterion, Et–(H_2_)N → C(OH)(=O)–Ph. In fact, the C(7)–N distance, 1.659 Å, is appreciably larger than the 1.494 one of N–C(5). The CT complex may intervene only when it is surrounded by the water cluster. Hydrogen bonds to two amino hydrogens enhance the nucleophilicity of the nitrogen n_p_. Those to the carbonyl oxygen enhance the electrophilicity of the carbonyl carbon. When the geometry of the CT complex moiety [Et–(H_2_)N----C(OH)(=O)–Ph] is taken up and is re-optimized by B3LYP/6-31(+)G(d) SCRF = PCM, Et–NH_2_ is completely separated from Ph–C(=O)–OH (infinite separation). On the other hand, when a geometry composed of Et(H_2_)N----C(=O)(OH)Ph and five H_2_O molecules is optimized by B3LYP/6-31(+)G(d) SCRF = PCM and B3PW91/6-311+G(d,p) SCRF = PCM, the CT-complex geometry is obtained (Figure S2). Thus, intervention of zwitter-ions and CT complexes should be described by cluster geometries with water molecules explicitly contained. This result is consistent with the proposal for the L-alanine zwitterion [38–46]. From the CT complex, the C(7)∙∙∙N bond scission occurs at TS3(am). After TS3(am), the {Ph–COOH + Et–NH_2_ + OH^−^(H_2_O)_15_} intermediate, Int3(am), is generated. The generation is followed by TS4(am), which leads to the product {Ph–COO^−^ + Et–NH_2_ + (H_2_O)_16_}.

Figure 5 demonstrates that the hydrolysis of *N*-ethylbenzamide has four elementary processes. A crucial difference between the ester and amide hydrolyses is found in TS2; TS2(es) leads to the separated Ph–COOH and EtOH, while TS2(am) to the CT complex, Et–(H_2_)N → C(OH)(=O)–Ph. The difference may be represented by that between the hard-base oxygen and soft-base nitrogen according to Pearson's HSAB concept [61].

### Energy changes along the reaction paths

**Figure 6:**
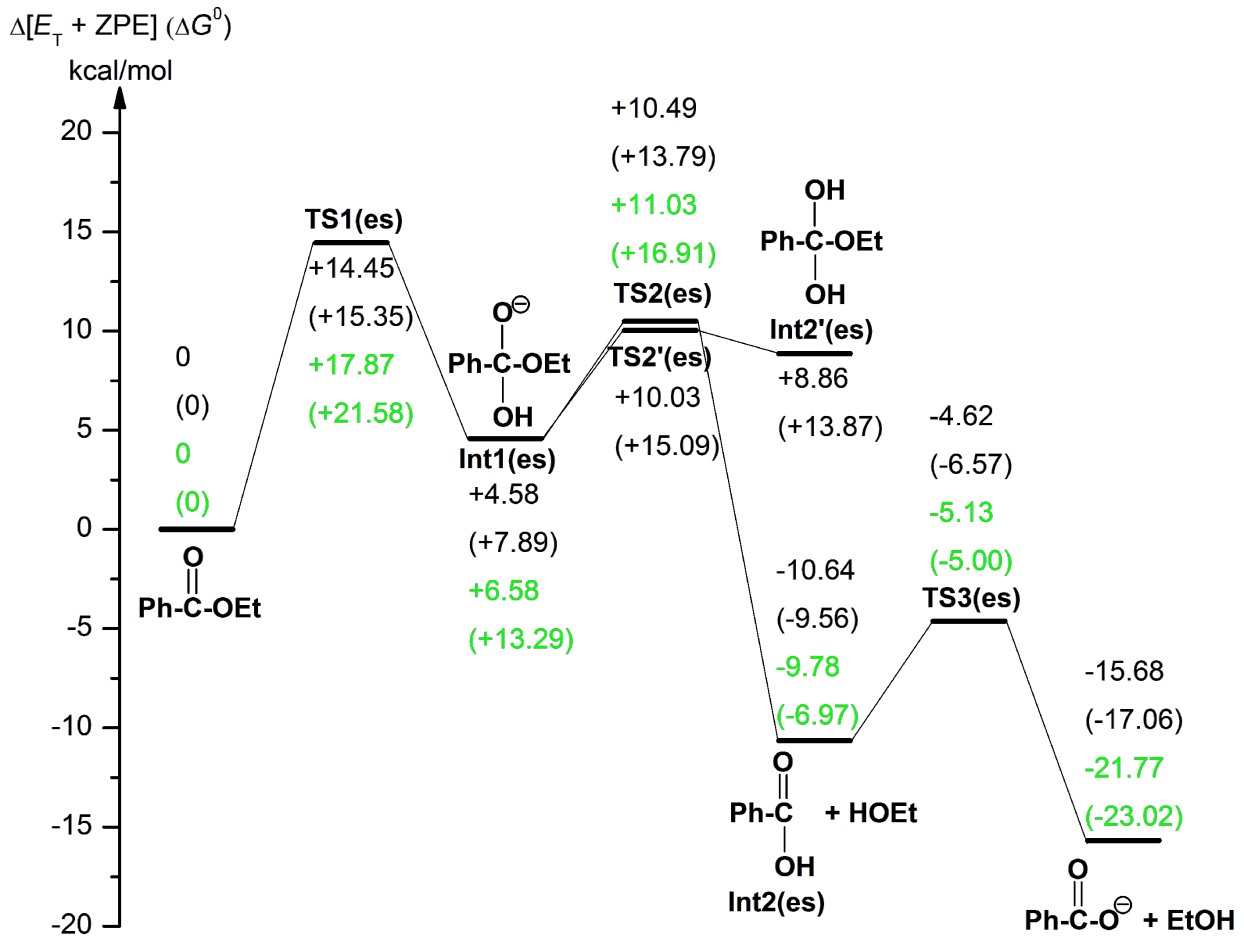
Changes of B3LYP/6-311++G(d,p) SCRF = PCM//B3LYP/6-31(+)G(d) Et + ZPE and (Gibbs free energies) of the ester hydrolysis in Figure 4 and Figure S3 (Supporting Information File 1). Energies given in green are for the Na^+^-containing reaction in Figure S3.

**Figure 7:**
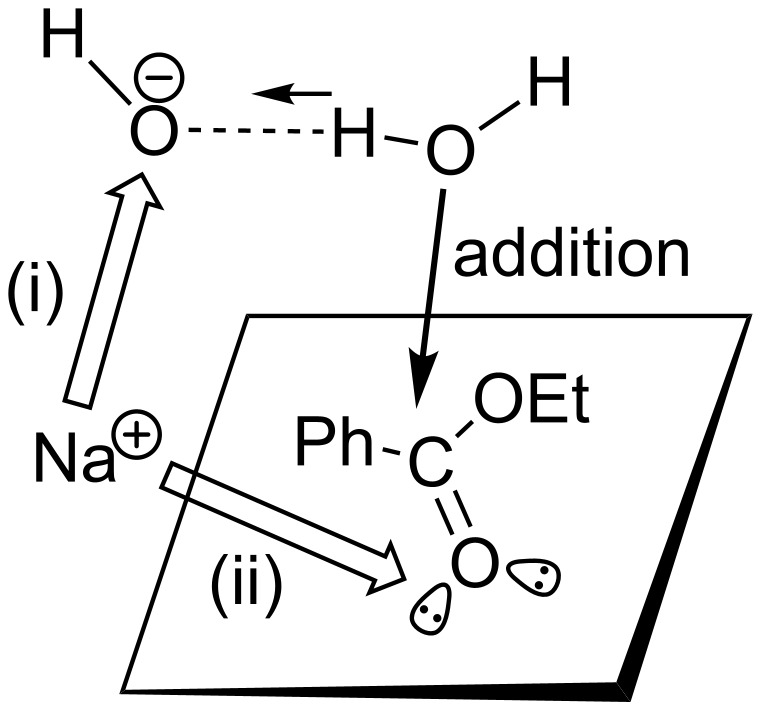
The effect of the counter ion Na^+^ on TS1(es). When the position of Na^+^ is near the nucleophile OH^−^ in (i), its addition is decelerated. On the contrary, when it is near the carbonyl oxygen in (ii), the addition is accelerated owing to the enhancement of the electrophilicity of the carbonyl carbon.

Figure 6 shows energy changes for the ester hydrolysis of Figure 4. Those of Na^+^-containing paths in the system, Ph–C(=O)–OEt + NaOH(H_2_O)_16_, are also shown in green. Geometric changes in the Na^+^-containing system are exhibited in Figure S3 (Supporting Information File 1). For the geometry optimization, the position of Na^+^ was assumed such that the reaction is promoted (i.e., (ii) in Figure 7).

In the changes of the Na^+^-free system (Figure 4), the rate-determining step was confirmed to be TS1(es), with the calculated activation energy +14.45 kcal/mol (exp., +14.6 kcal/mol [47]). While TS2'(es) has a slightly lower energy (= +10.03 kcal/mol) than that (= +10.49 kcal/mol) of TS2(es), the former leads to an unstable intermediate, Ph–C(OH)_2_–OEt, with the energy +8.86 kcal/mol. Therefore, the possibility {Ph–C(OH)_2_–OEt as the concentration stock} raised in Scheme 5 has been ruled out. The energy changes of Figure 6 (without Na^+^) demonstrate that the hydrolysis proceeds smoothly and suggest that intermediates may not be detected experimentally. When the Na^+^ ion is included in the system, the activation energy of TS1(es) is enlarged appreciably (= +17.87 kcal/mol) in spite of the positional assumption (ii) in Figure 7. Thus, the counter ion works unfavorably on the rate-determining step TS1(es). The Na^+^ ion has a very large hydration energy (= −97 kcal/mol), and the cation needs to be surrounded by many water molecules in the hydration shell far from the reaction region.

**Figure 8:**
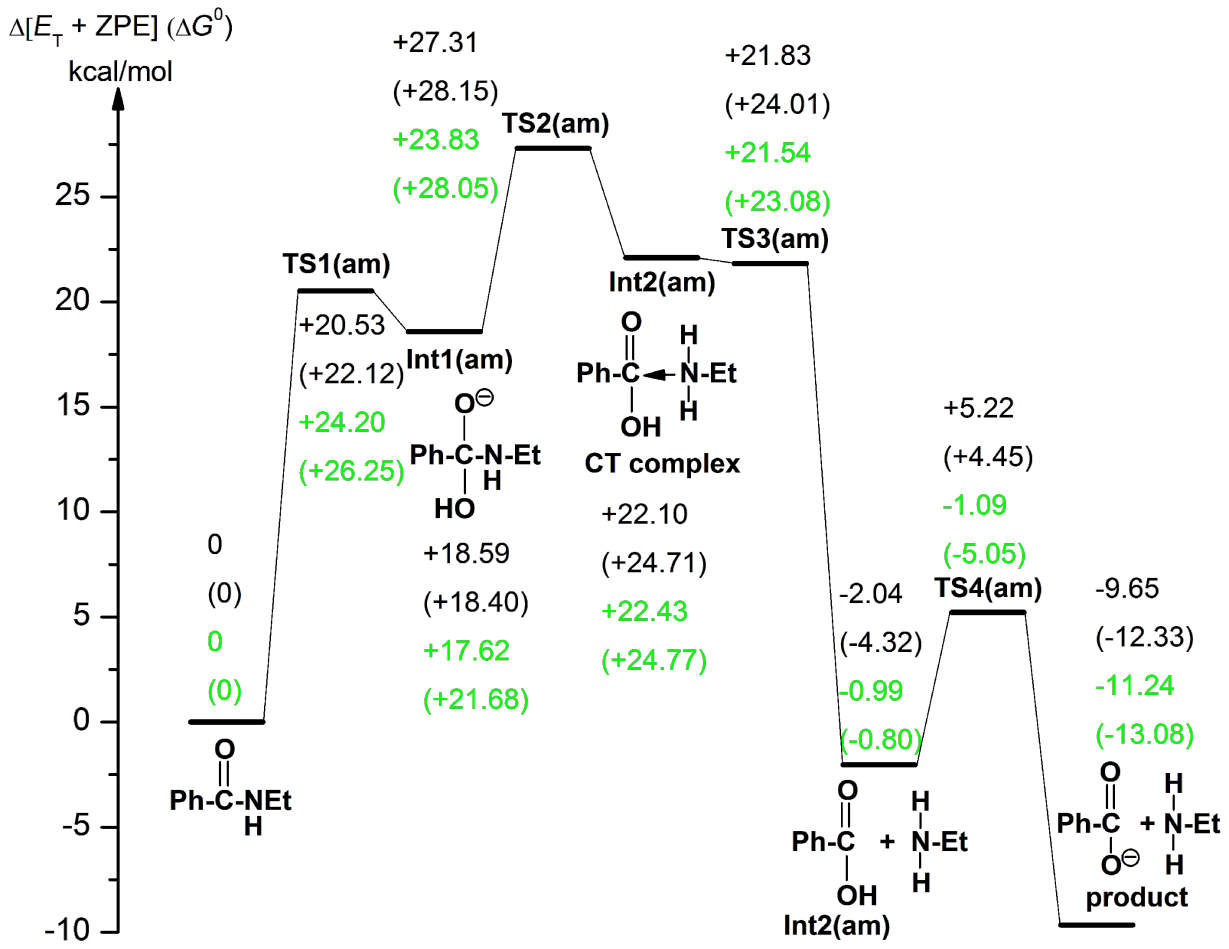
Changes of Et + ZPE and (Gibbs free energies) of the amide hydrolysis in Figure 5 and Figure S4 (Supporting Information File 1). Energies in green are for the Na^+^-containing reaction in Figure S4.

Figure 8 shows energy changes for the amide hydrolysis of Figure 5. Those of Na^+^-containing paths in the system, Ph–C(=O)–NH–Et + NaOH(H_2_O)_16_ in Figure S4 (Supporting Information File 1), are also shown in green. In energies of the Na^+^-free system, the rate-determining step was calculated to be TS2(am) with the activation energy 27.31 kcal/mol. This value is comparable to the experimental one 27.1 kcal/mol [22] in the basic hydrolysis of *N*,*N*-dimethyltoluamide (*para*-Me–C_6_H_4_–C(=O–NMe_2_)). This result is consistent with the experimental suggestion that the second TS may be rate-determining as shown in Figure 5 of [22]. However, it is in contrast with the general scheme that the first OH^−^ addition step is rate-determining [36]. The result of Ea{TS1(am)} < Ea{TS2(am)} was checked by re-optimizing their geometries with B3LYP/6-311+G(d,p). Activation (free) energies were calculated to be 23.29 (25.74) kcal/mol for TS1(am) and 25.69 (28.68) for TS2(am) (detailed data in VII.i, Supporting Information File 1).

In the B3LYP/6-31(+)G(d) Et + ZPE (without B3LYP/6-311++G(d,p) SCRF = PCM single-point calculations), +21.63 kcal/mol of TS1(am) is similar to +21.92 kcal/mol of TS2(am). This ambiguity at the computational level was removed in the (H_2_O)_16_-using hydrolyses of two para-substituted aromatic amides, Y–C_6_H_4_–C(=O)–NH–Et Y = MeO and O_2_N. For Y = MeO, +23.64 (+26.45) kcal/mol of TS2(am) is larger than +19.80 (+22.93) kcal/mol of TS1(am). For Y = O_2_N, also, +22.99 (+24.86) kcal/mol of TS2(am) is larger than +18.80 (+20.56) kcal/mol of TS1(am) (detailed data in VII.j, Supporting Information File 1). Thus, as far as the aromatic amide is concerned, TS2(am) is thought to be rate-determining.

The effect of the counter ion Na^+^ on activation energies was examined. The effect on free-energy changes of TS2(am), (+28.15 kcal/mol) and (+28.05 kcal/mol) was found to be small. Thus, in the amide hydrolysis of *n* = 16, the Na^+^ cation is separated well from the reaction center.

## Conclusion

**Scheme 6:**
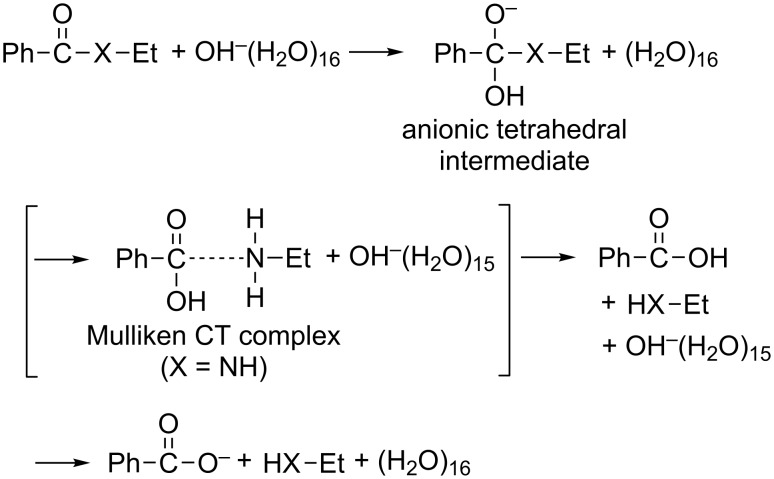
Summary of the present calculations.

In this work, reaction paths of base-catalyzed hydrolyses of isoelectronic substrates (ethyl benzoate and *N*-ethylbenzamide) were traced by DFT calculations. In Scheme 6, the obtained result is summarized.

**Figure 9:**
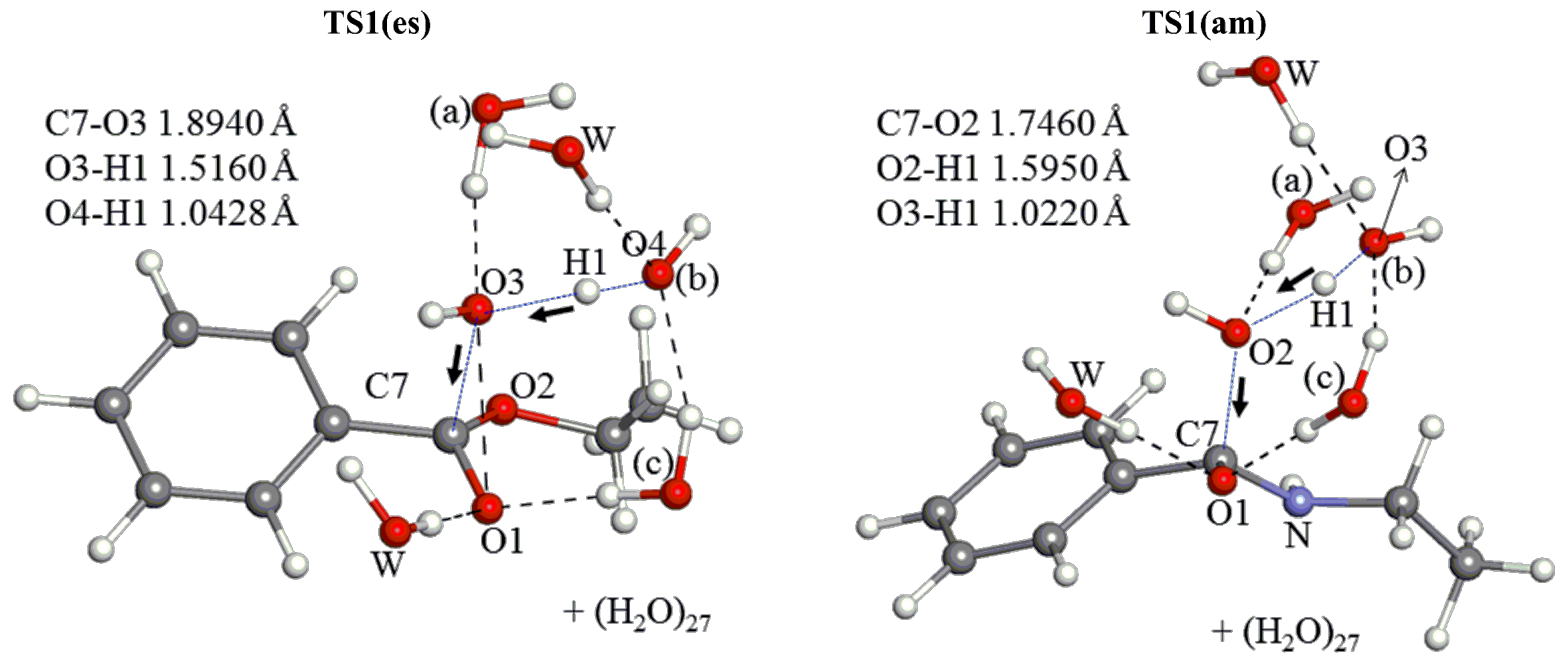
Central parts of the geometries of TS1(es) and TS1(am) of *n* = 32, which are taken from Figure 2 and Figure 3, respectively. Notations, (a), (b), (c) and W, are defined in Figure 1.

The five points 1–5 raised in the Introduction may be addressed on the basis of computational results:

1. The rate-determining step of the ester hydrolysis is the OH^−^ addition step, TS1(es). The energy change demonstrates that the reaction occurs spontaneously toward the product. On the other hand, that of the amide hydrolysis is TS2(am). TS2(am) is not at the "breakdown of the anionic tetrahedral intermediate" [21–25] but at the formation of the Mulliken CT complex.

2. The number of elementary processes is either three for the ester hydrolysis or four for the amide hydrolysis. The zwitterion suggested experimentally [25] is rather a Mulliken CT complex involved only in the amide hydrolysis. The intermediate is obtainable only in the H_2_O-containing cluster system.

3. At both TS1(es) and TS1(am), the general base mechanism is more likely than the direct nucleophilic process regardless of the number of water molecules (*n* > 3).

4. The counter ion Na^+^ works unfavorably on the hydrolysis, particularly on TS1(es). The ion should be separated from the reaction region in the hydration shell.

5. A minimal TS1 model composed of the substrate Ph–CO–X–Et, OH^−^(H_2_O)_3_ and W_2_ (W: catalytic water molecule) has been constructed in Figure 1. The model has been examined in Figure 2 and Figure 3 with the number (*n*) of water molecules, *n* = 3, 5, 8, 12, 16, 24 and 32. The model has been retained in all TS geometries, as exemplified in the *n* = 32 TS1(es) and TS1(am) (Figure 9).

This work has demonstrated that proton transfers along hydrogen bonds have a significant role on the progress of the hydrolysis.

## Supporting Information

Detailed geometric data along with those of complementary calculations. Figure S1 (geometry changes by the ADMP dynamical calculation), Figure S2 (the CT complex geometry), Figures S3 and S4 (reaction paths), Tables S1 and S2 (method dependence of TS1 geometries), and Cartesian coordinates of the optimized geometries.

File 1 Detailed geometric data along with those of complementary calculations.
