# Supplementary material for: Presence or absence of a novel charge-transfer complex in the base-catalyzed hydrolysis of N-ethylbenzamide or ethyl benzoate
Source: Beilstein J Org Chem. 2013 Jan 29;9:185–96. doi: 10.3762/bjoc.9.22 (PMC3566851; doi:10.3762/bjoc.9.22)
Supplement: File 1 — Detailed geometric data along with those of complementary calculations. [file Beilstein_J_Org_Chem-09-185-s001.pdf]

Supporting Information

for

Presence or absence of a novel charge-transfer complex in the base-catalyzed hydrolysis of *N*-ethylbenzamide or ethyl benzoate

Shinichi Yamabe*, Wei Guan and Shigeyoshi Sakaki

Address: Fukui Institute for Fundamental Chemistry, Kyoto University, Takano-Nishihiraki-cho 34-4, Sakyo-ku, Kyoto 606-8103, Japan, phone: +81-075-711-7907.

Email: yamabes@fukui.kyoto-u.ac.jp

*Corresponding author

Detailed geometric data along with those of complementary calculations

I. Figure S1 Geometric changes by an ADMP dynamical calculation from the TS1(es) geometry in the reaction of Ph-C(=O)-O-Et + $OH^-(H_2O)_{16}$. XYZ coordinates are in ESI VII [5]. S2

II. Figure S2 The geometry optimized by B3LYP/6-31(+)G(d) SCRF = PCM and (B3PW91/6-311+G(d,p) SCRF = PCM). Can the geometry of the Mulliken charge-transfer complex of benzoic acid (Ph-C(=O)OH) and ethylamine (H_5C_2-NH_2) be expressed by the aid of $(H_2O)_5$ and SCRF = PCM solvent effect? The answer is yes. XYZ coordinates are in ESI VII [6]. S2

III. Figure S3 Reaction paths of the hydrolysis in Ph-C(=O)-O-Et + $NaOH(H_2O)_{16}$. XYZ coordinates are in ESI VII [7]. S3

IV. Figure S4 Reaction paths of the hydrolysis in Ph-C(=O)-NH-Et + $NaOH(H_2O)_{16}$. XYZ coordinates are in ESI VII [8]. S4

V. Table S1 Dependence of computational methods on TS1(es) geometries in the n =16 ester hydrolysis. S5

VI. Table S2 Dependence of computational methods on TS1(am) geometries in the n =16 amide hydrolysis. S5

VII. Cartesian Coordinates of B3LYP/6-31(+)G(d) and B3LYP/6-311+G(d,p) geometry optimizations:

a. TS1(es) geometries in Figure 1. Hereafter, n denotes the number of water molecules around Ph-COO-Et and OH^-. S6–S19

b. TS1(am) geometries in Figure 2. Hereafter, n denotes the number of water molecules around Ph-CO-NH-Et and OH^-. S20–S33

c. Geometries of the n = 16 ester hydrolysis in Figure 3. S34–S51

d. Geometries of the n = 16 amide hydrolysis in Figure 4. S52–S70
e. The result of ADMP dynamical calculations starting from the n = 16 TS1(es) in Figures 1 and 3. The geometry after 800 femtoseconds shown in Figure S3. S71–S73
f. Figure S2. The geometry of the Mulliken CT complex composed of benzoic acid, ethylamine and $(H_2O)_5$. Stoichiometry $C_9H_{23}NO_7$. S74–S76
g. The Na^+-containing ester hydrolysis shown in Figure S3. S77–S91
h. The Na^+-containing amide hydrolysis shown in Figure S4. S92–S110
i. B3LYP/6-311+G(d,p) geometries of the reactant-like complex (precursor), TS1(am) and T2(am). S111–S116
j. para-Y-substituted N-ethylbenzamide + $OH^-(H_2O)_{16}$, Y = MeO and NO_2. Precursor, TS1(am) and TS2(am). S117–S129

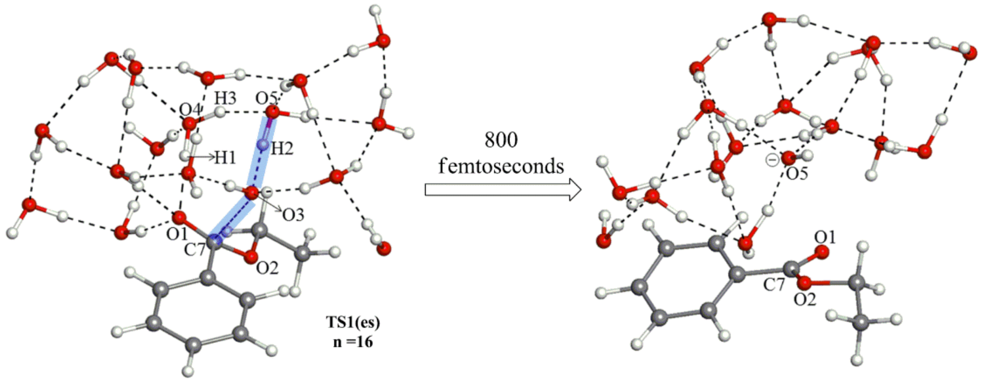

Figure S1 Geometric changes by an ADMP dynamical calculation from the TS1(es) geometry in the reaction of Ph-C(=O)-O-Et + $OH^-(H_2O)_{16}$. XYZ coordinates are in ESI VII [5].

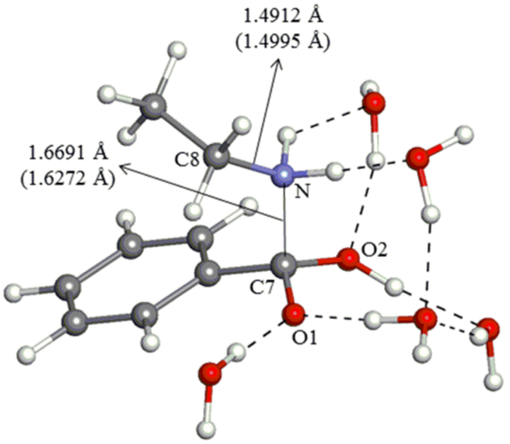

Figure S2 The geometry optimized by B3LYP/6-31(+)G(d) SCRF = PCM and (B3PW91/6-311+G(d,p) SCRF = PCM). Can the geometry of the Mulliken charge-transfer complex of benzoic acid (Ph-C(=O)OH) and ethylamine (H_5C_2-NH_2) be expressed by the aid of $(H_2O)_5$ and SCRF = PCM solvent effect? The answer is yes. XYZ coordinates are in ESI VII [6].

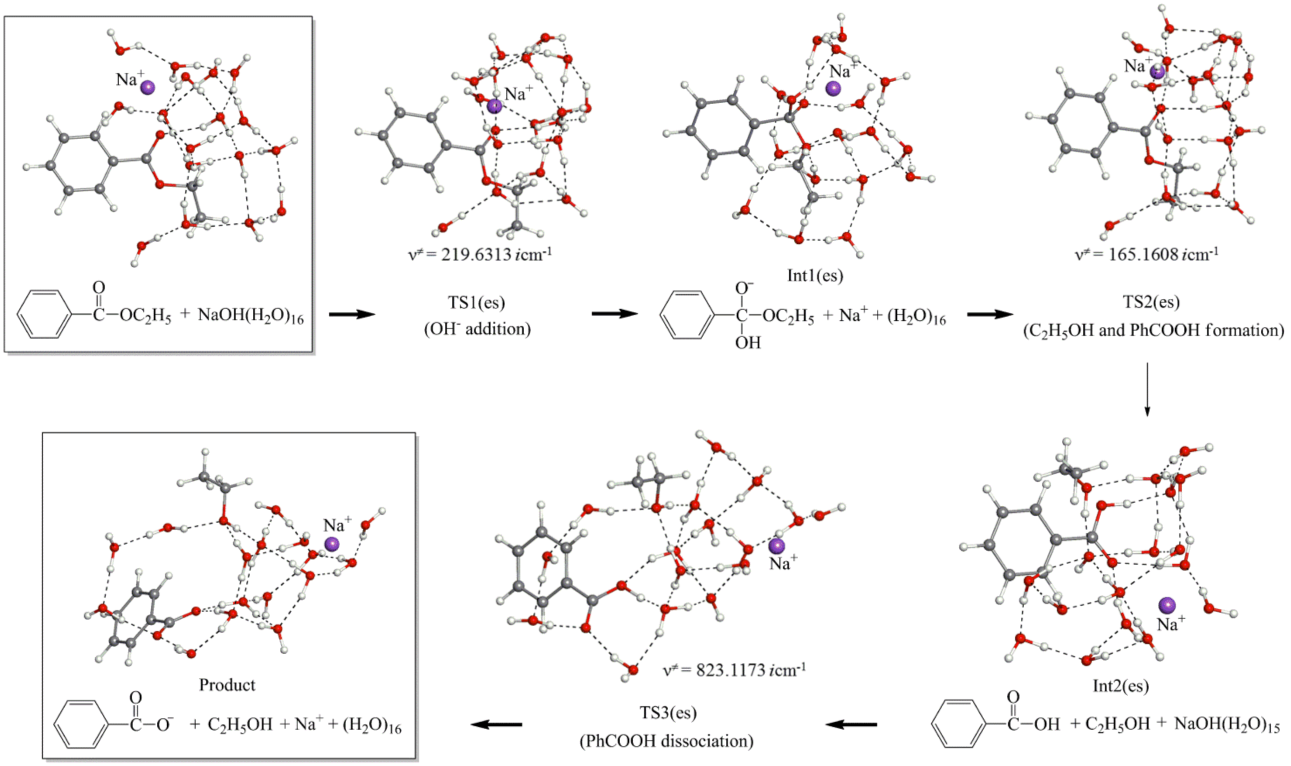

Figure S3 Reaction paths of the hydrolysis in Ph-C(=O)-O-Et + $NaOH(H_2O)_{16}$. XYZ coordinates are in ESI VII [7].

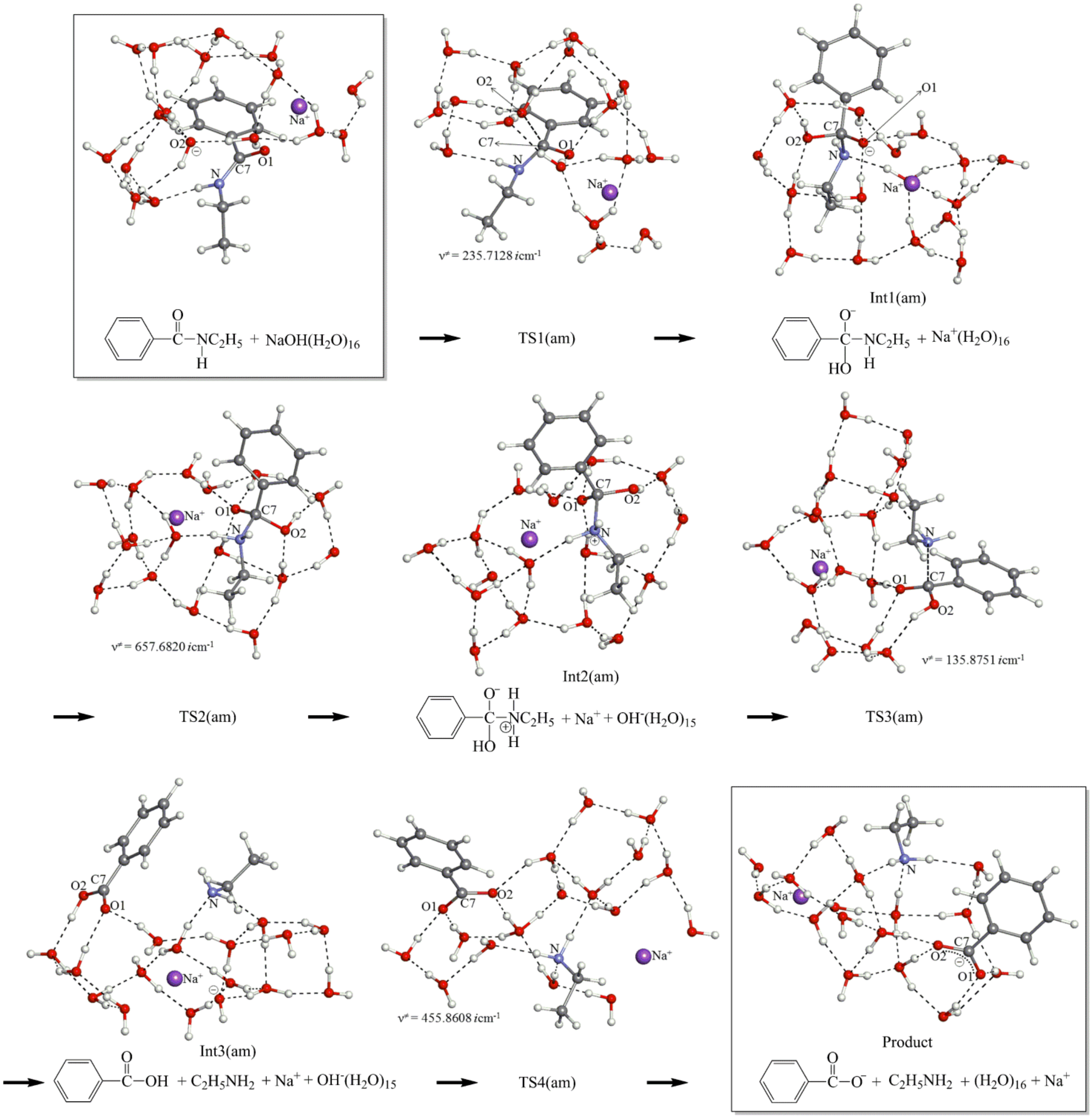

Figure S4 Reaction paths of the hydrolysis in Ph-C(=O)-NH-Et + $NaOH(H_2O)_{16}$. XYZ coordinates are in ESI VII [8].

Table S1 Dependence of computational methods on TS1(es) geometries in the *n* =16 ester hydrolysis.[a]

method	$\nu^{\neq}$ ($i$cm^{-1})	$C_7 \cdots O_3$ (Å)	$O_3 \cdots H_2$ (Å)	$O_5 \cdots H_2$ (Å)
B3LYP/6-31(+)G(d)	200.41	1.990	1.544	1.038
B3LYP/6-311+G(d,p)	192.01	1.955	1.570	1.023
B3PW91/6-31(+)G(d)	183.61	2.066	1.467	1.060
PCM/B3PW91/6-31(+)G(d)	249.05	2.014	1.565	1.032
B3PW91/6-311+G(d,p)	197.65	1.997	1.497	1.041
M06-2X/6-31(+)G(d)	278.88	2.041	1.522	1.033

a) The numbering of atoms is shown in Figure 1.

Table S2 Dependence of computational methods on TS1(am) geometries in the *n* =16 amide hydrolysis.[a]

method	$\nu^{\neq}$ ($i$cm^{-1})	$C_7 \cdots O_2$ (Å)	$O_2 \cdots H_2$ (Å)	$O_3 \cdots H_2$ (Å)
B3LYP/6-31(+)G(d)	152.25	1.723	1.617	1.018
B3LYP/6-311+G(d,p)	113.80	1.697	1.645	1.006
B3PW91/6-31(+)G(d)	227.51	1.867	1.576	1.027
PCM/B3PW91/6-31(+)G(d)	289.16	1.882	1.563	1.028
B3PW91/6-311+G(d,p)	215.21	1.761	1.574	1.018
M06-2X/6-31(+)G(d)	271.49	1.889	1.656	1.007

a) The numbering of atoms is shown in Figure 2.

Cartesian Coordinates of B3LYP/6-31(+)G(d) and B3LYP/6-311+G(d,p) geometry optimizations

[1] TS1(es) geometries in Figure 1. Hereafter, n denotes the number of water molecules around Ph-COO-Et and OH^-

n=3 TS

ethyl benzoate(C6H5-COO-C2H5) and HO-(H2O)3, HO-, addition TS
zuka03aa.chk

Standard orientation:

Center Number	Atomic Number	Atomic Type	Coordinates (Angstroms) X	Y	Z
1	6	0	0.728327	-0.592871	-0.389120
2	6	0	-0.511088	0.261283	-0.588849
3	8	0	-0.517536	1.223134	-1.391341
4	8	0	-0.585852	1.003929	1.104238
5	1	0	-1.038267	1.829453	0.861533
6	6	0	1.845269	-0.341286	-1.190251
7	6	0	2.998526	-1.117829	-1.060244
8	6	0	0.779497	-1.630564	0.550105
9	8	0	-1.643156	-0.587092	-0.537163
10	6	0	-2.870805	0.027511	-0.925427
11	1	0	-3.133839	0.812045	-0.202694
12	1	0	-2.749077	0.502127	-1.904450
13	6	0	-3.945021	-1.049177	-0.961851
14	1	0	0.609092	2.753086	-1.100161
15	8	0	1.194357	3.485434	-0.805908
16	1	0	1.527247	3.170954	0.059298
17	1	0	-2.442648	-1.183515	1.850487
18	8	0	-2.214787	-0.607065	2.595601
19	1	0	-1.597485	0.042708	2.155660
20	1	0	0.917052	1.656884	1.576863
21	8	0	1.722347	2.215201	1.802937
22	1	0	1.442228	2.741493	2.565831
23	1	0	-3.675452	-1.842499	-1.668081
24	1	0	-4.087112	-1.504216	0.025287
25	1	0	-4.903265	-0.616334	-1.275543
26	6	0	1.931181	-2.405695	0.679520
27	6	0	3.044460	-2.154351	-0.127274
28	1	0	1.795792	0.471892	-1.907118
29	1	0	3.862716	-0.908767	-1.687201
30	1	0	-0.078091	-1.813083	1.188482
31	1	0	1.962363	-3.204383	1.417732
32	1	0	3.943690	-2.758294	-0.022497

SCF Done: E(RB+HF-LYP) = -804.632753111 A.U. after 1 cycles
Convg = 0.1662D-08 -V/T = 2.0094
S**2 = 0.0000
Zero-point correction= 0.258592 (a.u.)
Thermal correction to Energy= 0.278339
Thermal correction to Enthalpy= 0.279283
Thermal correction to Gibbs Free Energy= 0.208811
Sum of electronic and zero-point Energies= -804.374161
Sum of electronic and thermal Energies= -804.354414
Sum of electronic and thermal Enthalpies= -804.353470
Sum of electronic and thermal Free Energies= -804.423942

n=5 TS

ethyl benzoate and HO-(H2O)5, HO- addition TS1(es)
zuka05a.chk
Standard orientation:

1	6	0	0.600624	-0.780942	-0.664532
2	6	0	-0.789370	-0.190240	-0.659086
3	8	0	-1.099653	0.761281	-1.409456
4	8	0	-0.660679	0.616081	1.151754
5	1	0	-1.337170	1.312738	1.064544
6	6	0	1.482174	-0.399055	-1.680628
7	6	0	2.766917	-0.942415	-1.743213
8	6	0	1.017355	-1.713401	0.294353
9	8	0	-1.716188	-1.180540	-0.375215
10	6	0	-3.092369	-0.794796	-0.488615
11	1	0	-3.308041	-0.012796	0.249247
12	1	0	-3.271029	-0.382743	-1.487143
13	6	0	-3.945288	-2.027973	-0.240266
14	1	0	-0.031149	2.413693	-1.257499
15	8	0	0.263342	3.292104	-0.935891
16	1	0	0.787736	3.072830	-0.135334
17	1	0	-1.863412	-1.909431	2.160696
18	8	0	-1.521889	-1.328218	2.856315
19	1	0	-1.185733	-0.548566	2.327189
20	1	0	0.633115	1.470606	1.338222
21	8	0	1.415846	2.140087	1.432876
22	1	0	1.275295	2.567557	2.290789
23	1	0	-3.708954	-2.817905	-0.961741
24	1	0	-3.786939	-2.424197	0.768881
25	1	0	-5.006946	-1.772702	-0.340404
26	6	0	2.300601	-2.255017	0.231788

27	6	0	3.179779	-1.874386	-0.788560
28	1	0	1.150443	0.326593	-2.416093
29	1	0	3.445587	-0.634660	-2.534832
30	1	0	0.345988	-1.984667	1.101108
31	1	0	2.619712	-2.968997	0.987064
32	1	0	4.180073	-2.299206	-0.835262
33	1	0	-2.354436	2.159263	-0.753496
34	8	0	-2.520897	2.960124	-0.217814
35	1	0	-1.668038	3.431795	-0.321215
36	1	0	3.105899	1.376397	1.398509
37	8	0	3.974029	0.911508	1.365319
38	1	0	3.784176	0.114324	0.846228

SCF Done: E(RB+HF-LYP) = -957.521199563 A.U. after 1 cycles
Convg = 0.2048D-08 -V/T = 2.0093
S**2 = 0.0000
Zero-point correction= 0.309377 (a.u.)
Thermal correction to Energy= 0.334313
Thermal correction to Enthalpy= 0.335257
Thermal correction to Gibbs Free Energy= 0.253374
Sum of electronic and zero-point Energies= -957.211822
Sum of electronic and thermal Energies= -957.186887
Sum of electronic and thermal Enthalpies= -957.185943
Sum of electronic and thermal Free Energies= -957.267825

n=8 TS

ethyl benzoate and HO-(H2O)8, HO- addtion TS
zuka03cc.log
Standard orientation:

Center Number	Atomic Number	Atomic Type	Coordinates (Angstroms) X	Y	Z
1	6	0	-1.073567	-0.895704	-0.625382
2	6	0	-1.793402	0.408425	-0.291537
3	8	0	-1.334862	1.511745	-0.718983
4	8	0	-1.635750	0.424046	1.434157
5	1	0	-1.761439	1.375504	1.593949
6	6	0	-0.098352	-0.877172	-1.626124
7	6	0	0.576375	-2.047622	-1.983592
8	6	0	-1.372906	-2.104943	0.016980
9	8	0	-3.182115	0.167471	-0.454536
10	6	0	-4.004193	1.336830	-0.497305
11	1	0	-3.994129	1.833901	0.483403

12	1	0	-3.599734	2.038862	-1.232472
13	6	0	-5.415839	0.902311	-0.859323
14	1	0	0.190741	2.072760	-0.189854
15	8	0	1.066981	2.341163	0.214288
16	1	0	1.186752	1.712777	0.958133
17	1	0	-4.292065	-1.001827	1.540633
18	8	0	-3.873784	-0.933442	2.412761
19	1	0	-3.042994	-0.445100	2.193733
20	1	0	-0.081249	0.199837	1.898997
21	8	0	0.909861	0.218151	2.116011
22	1	0	0.983646	0.288655	3.079200
23	1	0	1.940578	-1.058635	1.358326
24	8	0	2.510877	-1.713603	0.889800
25	1	0	1.898862	-2.228205	0.337169
26	1	0	1.824678	4.192662	0.451091
27	8	0	2.539759	4.849123	0.333473
28	1	0	3.203378	4.337511	-0.161351
29	1	0	2.481789	2.252747	-0.781142
30	8	0	3.370153	2.328010	-1.226264
31	1	0	3.179389	2.410253	-2.172358
32	1	0	3.618797	-0.746570	-0.350175
33	8	0	4.371651	-0.364679	-0.848556
34	1	0	4.158859	0.587206	-0.945454
35	1	0	4.525879	-2.146995	1.425518
36	8	0	5.480516	-2.008099	1.290382
37	1	0	5.487364	-1.365848	0.552756
38	1	0	-5.426979	0.394418	-1.829828
39	1	0	-5.827396	0.216739	-0.109588
40	1	0	-6.075748	1.776444	-0.917315
41	6	0	-0.696091	-3.273583	-0.334006
42	6	0	0.281148	-3.250512	-1.335938
43	1	0	0.124919	0.065817	-2.114375
44	1	0	1.338607	-2.017681	-2.757870
45	1	0	-2.124573	-2.125282	0.799243
46	1	0	-0.929497	-4.204683	0.176776
47	1	0	0.806235	-4.162542	-1.610031

SCF Done: E(RB+HF-LYP) = -1186.84664368 A.U. after 1 cycles

Convg = 0.2672D-08 -V/T = 2.0093

S**2 = 0.0000

Zero-point correction= 0.385558 (a.u.)

Thermal correction to Energy= 0.418605

Thermal correction to Enthalpy= 0.419550

Thermal correction to Gibbs Free Energy= 0.316946

Sum of electronic and zero-point Energies= -1186.461085

Sum of electronic and thermal Energies= -1186.428038

Sum of electronic and thermal Enthalpies= -1186.427094
Sum of electronic and thermal Free Energies= -1186.529697

n=12 TS

ethyl benzoate and HO-(H2O)8+4, HO- addition TS
zuka03dd.chk

Standard orientation:

Center Number	Atomic Number	Atomic Type	Coordinates (Angstroms) X	Y	Z
1	6	0	-0.414011	-0.492230	-1.266906
2	6	0	-0.935588	0.764169	-0.597454
3	8	0	-0.134895	1.680437	-0.230856
4	8	0	-1.537625	0.034866	0.964587
5	1	0	-1.508808	0.830098	1.524497
6	6	0	0.944209	-0.566339	-1.587916
7	6	0	1.461469	-1.694325	-2.232460
8	6	0	-1.255953	-1.564744	-1.592771
9	8	0	-2.135412	1.136008	-1.198301
10	6	0	-2.626848	2.447038	-0.871164
11	1	0	-2.854870	2.492585	0.201727
12	1	0	-1.851352	3.188531	-1.082246
13	6	0	-3.872704	2.696006	-1.704164
14	1	0	1.031594	1.365259	1.053926
15	8	0	1.658493	1.182243	1.800979
16	1	0	1.359077	0.326699	2.173228
17	1	0	-4.215009	-0.185096	-0.307764
18	8	0	-4.100582	-0.580909	0.570568
19	1	0	-3.131527	-0.396961	0.777287
20	1	0	-0.400972	-0.920889	1.704727
21	8	0	0.369086	-1.334187	2.212446
22	1	0	-0.011830	-1.751372	2.999432
23	1	0	1.483507	-2.459399	1.293842
24	8	0	2.082144	-3.043897	0.773897
25	1	0	1.620227	-3.184283	-0.068751
26	1	0	2.501174	2.697169	2.460575
27	8	0	3.125118	3.447048	2.588488
28	1	0	3.977431	3.012093	2.749641
29	1	0	3.355075	0.953815	0.988086
30	8	0	4.237346	0.994749	0.561168
31	1	0	4.223885	1.864386	0.099269
32	1	0	3.596398	-1.974214	0.080876
33	8	0	4.458500	-1.703474	-0.292624

34	1	0	4.546029	-0.751094	-0.062897
35	1	0	3.896266	-4.136325	1.178577
36	8	0	4.856520	-4.246693	1.062964
37	1	0	5.106092	-3.414751	0.612383
38	1	0	-3.644556	2.626249	-2.772868
39	1	0	-4.663738	1.973232	-1.473438
40	1	0	-4.263022	3.699006	-1.497820
41	6	0	-0.741545	-2.693199	-2.228505
42	6	0	0.618498	-2.761125	-2.552689
43	1	0	1.601078	0.255209	-1.320812
44	1	0	2.523259	-1.740156	-2.457025
45	1	0	-2.309236	-1.518428	-1.338851
46	1	0	-1.401822	-3.522333	-2.469751
47	1	0	1.017737	-3.640558	-3.052095
48	1	0	0.730163	2.869154	-1.215726
49	8	0	1.279567	3.517427	-1.721760
50	1	0	1.482372	3.077295	-2.560433
51	1	0	2.884250	3.698604	-0.755681
52	8	0	3.716904	3.667113	-0.235048
53	1	0	3.455000	3.840791	0.694014
54	1	0	-5.341055	-2.289549	0.836411
55	8	0	-6.196348	-2.627320	1.159526
56	1	0	-6.556875	-1.850146	1.626339
57	1	0	-5.374127	0.014434	1.717166
58	8	0	-6.185326	0.124965	2.269264
59	1	0	-5.874901	0.152414	3.185963

SCF Done: E(RB+HF-LYP) = -1492.61180227 A.U. after 1 cycles
Convg = 0.3406D-08 -V/T = 2.0093
S**2 = 0.0000
Zero-point correction= 0.487061 (a.u.)
Thermal correction to Energy= 0.531069
Thermal correction to Enthalpy= 0.532013
Thermal correction to Gibbs Free Energy= 0.400716
Sum of electronic and zero-point Energies= -1492.124742
Sum of electronic and thermal Energies= -1492.080733
Sum of electronic and thermal Enthalpies= -1492.079789
Sum of electronic and thermal Free Energies= -1492.211086

n=16 TS

ethyl benzoate + HO-(H2O)16 TS1(es)
molecular formula: C9H43O19(1-)
zuka02aa.chk
Standard orientation:

Center Number	Atomic Number	Atomic Type	Coordinates (Angstroms) X	Y	Z
1	6	0	-0.029063	2.670394	-0.007977
2	6	0	-0.333003	1.442850	0.812460
3	8	0	-1.376670	0.754484	0.605280
4	8	0	0.171988	1.585091	2.066932
5	6	0	-0.139275	0.572461	3.039007
6	6	0	-1.002168	3.171408	-0.877904
7	6	0	-0.749097	4.321731	-1.629854
8	6	0	1.198353	3.337491	0.111139
9	6	0	0.566257	0.950146	4.329589
10	1	0	-1.223399	0.524903	3.180571
11	1	0	0.203927	-0.397340	2.667420
12	8	0	1.112393	0.292708	0.071213
13	1	0	0.932669	0.320348	-0.890057
14	1	0	1.114067	-1.184518	0.519889
15	8	0	1.192987	-2.180322	0.801716
16	1	0	1.845657	-2.191722	1.527157
17	1	0	2.282543	-2.529205	-0.575781
18	8	0	3.048589	-2.485507	-1.207847
19	1	0	3.408064	-1.583854	-1.018809
20	1	0	4.170642	-3.491734	-0.329493
21	8	0	4.696286	-3.933476	0.394713
22	1	0	4.341013	-4.832240	0.461187
23	1	0	2.010705	-2.321772	-2.810826
24	8	0	1.402411	-2.060926	-3.535093
25	1	0	0.524611	-2.466652	-3.319601
26	1	0	1.016843	-0.447951	-3.233872
27	8	0	0.682451	0.445165	-2.919219
28	1	0	1.211674	1.125550	-3.359063
29	1	0	4.222380	-2.794697	1.660728
30	8	0	3.807768	-2.051158	2.172094
31	1	0	4.304338	-1.966378	2.999044
32	1	0	-1.780425	0.266555	-1.176166
33	8	0	-1.957116	0.073115	-2.124303
34	1	0	-1.149027	0.394229	-2.581711
35	1	0	-1.491720	-1.730337	-2.649809
36	8	0	-1.210496	-2.637471	-2.894836
37	1	0	-1.749771	-3.194595	-2.285945
38	1	0	-3.752133	-0.711160	-2.126512
39	8	0	-4.520913	-1.258597	-1.870622
40	1	0	-5.010333	-0.733016	-1.199075
41	1	0	-1.479635	-1.082881	0.892639
42	8	0	-1.537605	-2.041667	1.114426

43	1	0	-0.594979	-2.336076	1.095771
44	1	0	-2.722830	-2.085136	2.424479
45	8	0	-3.492898	-1.889416	3.015905
46	1	0	-4.215209	-2.436026	2.670742
47	1	0	-2.530443	-3.008100	-0.197370
48	8	0	-3.023201	-3.456601	-0.916608
49	1	0	-3.672187	-2.781392	-1.227886
50	1	0	2.757130	0.240645	0.083527
51	8	0	3.733753	-0.035893	0.041959
52	1	0	3.882110	-0.538171	0.868944
53	1	0	4.855338	1.390809	-0.064081
54	8	0	5.487433	2.140200	-0.180177
55	1	0	5.722263	2.112831	-1.118899
56	1	0	-3.796555	-0.141584	2.455045
57	8	0	-3.878579	0.723874	1.990145
58	1	0	-3.022635	0.840248	1.523462
59	1	0	-5.125077	0.487778	0.813224
60	8	0	-5.812308	0.299351	0.117364
61	1	0	-6.118306	1.164679	-0.190050
62	6	0	1.449119	4.483124	-0.640012
63	6	0	0.475828	4.978745	-1.514168
64	1	0	-1.953836	2.658323	-0.964538
65	1	0	-1.512870	4.701300	-2.304012
66	1	0	1.955900	2.940920	0.777266
67	1	0	2.408934	4.984545	-0.546915
68	1	0	0.674130	5.872185	-2.101826
69	1	0	1.650787	0.994343	4.183860
70	1	0	0.226743	1.928073	4.686954
71	1	0	0.347041	0.204541	5.102382

SCF Done: E(RB+HF-LYP) = -1798.39889531 A.U. after 1 cycles
Convg = 0.2155D-08 -V/T = 2.0093
S**2 = 0.0000
Zero-point correction= 0.595360 (a.u.)
Thermal correction to Energy= 0.646200
Thermal correction to Enthalpy= 0.647145
Thermal correction to Gibbs Free Energy= 0.506737
Sum of electronic and zero-point Energies= -1797.803536
Sum of electronic and thermal Energies= -1797.752695
Sum of electronic and thermal Enthalpies= -1797.751751
Sum of electronic and thermal Free Energies= -1797.892158

n=24 TS

ethyl benzoate + HO-(H2O)16+8 TS1(es) n=24

molecular formula: C9H59O27(1-)
zuka02jj.chk

Standard orientation:

Center Number	Atomic Number	Atomic Type	X	Y	Z
1	6	0	0.231153	-2.101990	2.247982
2	6	0	-0.240438	-2.091777	0.815798
3	8	0	-1.197209	-1.321989	0.455577
4	8	0	-0.102901	-3.320129	0.265703
5	6	0	-0.723599	-3.580083	-1.011287
6	6	0	-0.488075	-1.393435	3.215261
7	6	0	-0.076800	-1.407582	4.550700
8	6	0	1.360033	-2.839215	2.632113
9	6	0	-0.443431	-5.024520	-1.378149
10	1	0	-1.799038	-3.402566	-0.936764
11	1	0	-0.311729	-2.889657	-1.754423
12	8	0	1.336591	-1.232019	0.044905
13	1	0	1.334708	-0.375930	0.517617
14	1	0	1.322312	-0.944645	-1.427901
15	8	0	1.320102	-0.682757	-2.448945
16	1	0	1.552087	-1.483604	-2.943220
17	1	0	2.732326	0.434606	-2.365037
18	8	0	3.503142	0.920410	-1.978729
19	1	0	3.789866	0.311573	-1.240823
20	1	0	4.993376	1.070126	-2.923689
21	8	0	5.905135	1.074115	-3.311413
22	1	0	6.055825	1.975289	-3.631172
23	1	0	2.797781	2.266346	-1.078030
24	8	0	2.480091	2.934748	-0.416395
25	1	0	1.578943	3.226308	-0.694743
26	1	0	2.074491	1.931546	0.995405
27	8	0	1.676902	1.318416	1.665021
28	1	0	2.351195	1.154773	2.341063
29	1	0	6.113145	-2.737728	-1.800379
30	8	0	6.143228	-2.345061	-0.915963
31	1	0	6.708848	-1.525432	-1.006502
32	1	0	-1.062856	0.405150	0.884262
33	8	0	-0.996019	1.365457	1.120476
34	1	0	-0.163455	1.431132	1.639242
35	1	0	-0.485193	2.612644	-0.129860
36	8	0	-0.258874	3.359399	-0.735855
37	1	0	-0.855062	3.204520	-1.499665
38	1	0	-2.622228	2.131759	0.990407

39	8	0	-3.439917	2.567485	0.653383
40	1	0	-4.141192	1.857116	0.627390
41	1	0	-1.274490	-0.649169	-1.185385
42	8	0	-1.357402	-0.235296	-2.081361
43	1	0	-0.440479	-0.299506	-2.444533
44	1	0	-2.909267	-1.013056	-2.669683
45	8	0	-3.860527	-1.267440	-2.649263
46	1	0	-4.322137	-0.477201	-3.016846
47	1	0	-2.098357	1.384019	-2.021264
48	8	0	-2.538403	2.270228	-2.069947
49	1	0	-2.938315	2.422927	-1.182230
50	1	0	3.120145	-1.206007	0.144393
51	8	0	4.038726	-0.831662	0.045230
52	1	0	4.650561	-1.540458	-0.270794
53	1	0	5.201889	0.408121	0.914472
54	8	0	5.951310	1.022875	1.061264
55	1	0	5.573317	1.928462	1.042223
56	1	0	-4.029139	-1.475559	-0.886374
57	8	0	-4.002729	-1.660386	0.090139
58	1	0	-3.047659	-1.628826	0.326971
59	1	0	-4.901877	-0.241470	0.795767
60	8	0	-5.307293	0.641876	0.965038
61	1	0	-5.357724	0.783514	1.928690
62	6	0	1.768608	-2.851777	3.963035
63	6	0	1.052654	-2.133690	4.926735
64	1	0	-1.372989	-0.837909	2.923523
65	1	0	-0.644475	-0.852756	5.293022
66	1	0	1.916856	-3.383395	1.877701
67	1	0	2.650921	-3.418064	4.249535
68	1	0	1.375787	-2.142656	5.964741
69	1	0	0.631169	-5.204986	-1.492069
70	1	0	-0.828643	-5.697400	-0.605354
71	1	0	-0.951591	-5.258166	-2.319147
72	1	0	6.985690	0.470367	-1.896483
73	8	0	7.411240	0.052505	-1.115968
74	1	0	7.017762	0.504243	-0.329484
75	1	0	3.926598	3.569631	0.433295
76	8	0	4.759566	3.630658	0.957050
77	1	0	5.291918	4.321884	0.538063
78	1	0	-1.351277	4.735394	0.131468
79	8	0	-2.107816	5.164138	0.584198
80	1	0	-2.785931	4.462966	0.619201
81	1	0	-3.967110	-3.221330	-3.024355
82	8	0	-3.900687	-4.191698	-2.936898
83	1	0	-4.252146	-4.374834	-2.042116
84	1	0	-3.941616	1.766770	-3.084115

85	8	0	-4.656848	1.263092	-3.548758
86	1	0	-4.593099	1.504896	-4.483944
87	1	0	-2.601001	6.878876	0.158399
88	8	0	-2.812105	7.837345	0.079594
89	1	0	-2.427503	8.228224	0.877386
90	1	0	-4.282606	2.746552	2.490549
91	8	0	-4.818145	2.371916	3.220629
92	1	0	-5.445695	3.066933	3.467505
93	1	0	-4.591780	-3.380903	0.044066
94	8	0	-4.886318	-4.295433	-0.182493
95	1	0	-5.853212	-4.275368	-0.132879

SCF Done: E(RB+HF-LYP) = -2409.92783513 A.U. after 1 cycles
Convg = 0.3469D-08 -V/T = 2.0092
S**2 = 0.0000
Zero-point correction= 0.799991 (a.u.)
Thermal correction to Energy= 0.871243
Thermal correction to Enthalpy= 0.872187
Thermal correction to Gibbs Free Energy= 0.684917
Sum of electronic and zero-point Energies= -2409.127844
Sum of electronic and thermal Energies= -2409.056592
Sum of electronic and thermal Enthalpies= -2409.055648
Sum of electronic and thermal Free Energies= -2409.242918

n=32 TS

ethyl benzoate and HO-(H2O)32, TS1(es)
zuka02ww.g09.chk Stoichiometry: C9H75O35(1-)
Standard orientation:

Center Number	Atomic Number	Atomic Type	Coordinates (Angstroms) X	Y	Z
1	6	0	-0.732364	-1.234766	2.884903
2	6	0	-1.020399	-1.308196	1.403009
3	8	0	-1.897030	-0.533649	0.878609
4	8	0	-0.901938	-2.594485	0.977416
5	6	0	-1.389196	-2.944300	-0.334236
6	6	0	-1.525368	-0.426985	3.705031
7	6	0	-1.287154	-0.370067	5.080585
8	6	0	0.293631	-1.999132	3.458419
9	6	0	-1.205561	-4.439966	-0.511885
10	1	0	-2.445951	-2.672698	-0.409616
11	1	0	-0.830966	-2.376661	-1.087668
12	8	0	0.624594	-0.628838	0.755042

13	1	0	0.707238	0.259244	1.164311
14	1	0	0.769308	-0.345309	-0.727551
15	8	0	0.940585	-0.064924	-1.717274
16	1	0	1.413617	-0.803162	-2.134948
17	1	0	2.110603	1.175986	-1.317040
18	8	0	2.815092	1.848072	-1.127309
19	1	0	3.088258	1.739819	-0.176498
20	1	0	4.138968	1.453544	-2.014384
21	8	0	4.974793	1.090767	-2.441080
22	1	0	4.778180	0.931806	-3.389673
23	1	0	1.857788	3.431206	-0.755832
24	8	0	1.533549	4.120411	-0.137363
25	1	0	0.611751	4.336518	-0.409368
26	1	0	1.118318	2.912402	1.212357
27	8	0	0.969004	2.194960	1.874658
28	1	0	1.879766	2.012634	2.184649
29	1	0	4.243675	-0.689106	-2.107747
30	8	0	3.841875	-1.576765	-2.234540
31	1	0	4.587037	-2.198722	-2.060475
32	1	0	-1.814043	1.261333	1.270265
33	8	0	-1.770613	2.213886	1.528978
34	1	0	-0.917190	2.281852	2.010300
35	1	0	-1.410165	3.523692	0.232701
36	8	0	-1.245509	4.235379	-0.429639
37	1	0	-1.815988	3.973260	-1.180205
38	1	0	-3.533594	2.880296	1.340565
39	8	0	-4.294182	3.180300	0.797739
40	1	0	-5.061971	2.613726	1.037844
41	1	0	-1.799188	-0.006170	-0.798431
42	8	0	-1.804913	0.351312	-1.725130
43	1	0	-0.859481	0.302031	-1.995059
44	1	0	-3.197122	-0.495349	-2.543799
45	8	0	-4.142083	-0.762887	-2.635516
46	1	0	-4.594164	0.017449	-3.026695
47	1	0	-2.751726	1.921286	-1.689377
48	8	0	-3.325850	2.722114	-1.754521
49	1	0	-3.727207	2.857625	-0.855119
50	1	0	2.092125	-1.399508	0.775444
51	8	0	3.010821	-1.780836	0.581378
52	1	0	3.098575	-1.765536	-0.393653
53	1	0	4.228121	-3.028499	1.234685
54	8	0	5.175172	-3.252394	1.400506
55	1	0	5.507074	-2.435380	1.832209
56	1	0	-4.416835	-0.754509	-0.836345
57	8	0	-4.567199	-0.831918	0.137589
58	1	0	-3.668315	-0.856031	0.542035

59	1	0	-5.579039	0.475946	0.771416
60	8	0	-6.226294	1.190198	0.998440
61	1	0	-6.945584	0.729004	1.486264
62	6	0	0.530538	-1.939610	4.829132
63	6	0	-0.258285	-1.122417	5.644901
64	1	0	-2.330548	0.153156	3.268252
65	1	0	-1.909450	0.263611	5.706745
66	1	0	0.902528	-2.631002	2.821153
67	1	0	1.333799	-2.529741	5.262838
68	1	0	-0.069625	-1.074531	6.714462
69	1	0	-0.143860	-4.710805	-0.510652
70	1	0	-1.701567	-4.980427	0.300726
71	1	0	-1.665112	-4.760248	-1.452670
72	1	0	6.237282	0.295090	-1.423710
73	8	0	6.832623	-0.134510	-0.765700
74	1	0	6.870517	-1.083633	-1.014804
75	1	0	3.854405	-0.984060	-4.119406
76	8	0	4.116051	-0.284298	-4.755237
77	1	0	3.340691	-0.108332	-5.308336
78	1	0	-2.569335	5.658939	0.296994
79	8	0	-3.424513	6.012151	0.607307
80	1	0	-3.957415	5.206796	0.743358
81	1	0	-5.095526	-2.388329	-2.859942
82	8	0	-5.690263	-3.164059	-2.788415
83	1	0	-6.312314	-2.927565	-2.069533
84	1	0	-4.597899	2.212332	-2.753114
85	8	0	-5.293153	1.711941	-3.275270
86	1	0	-5.408832	2.180293	-4.115052
87	1	0	5.770598	-2.947519	-0.265496
88	8	0	6.113364	-2.840574	-1.189776
89	1	0	6.550020	-3.699570	-1.342172
90	1	0	4.396548	-0.738686	1.218331
91	8	0	5.246147	-0.416431	1.605464
92	1	0	5.834859	-0.240968	0.837538
93	1	0	-6.219700	-1.755753	-0.159170
94	8	0	-7.110930	-1.840660	-0.559379
95	1	0	-7.228084	-0.992566	-1.060025
96	1	0	6.151187	-4.809989	0.824634
97	8	0	6.681134	-5.303040	0.163770
98	1	0	6.459417	-6.238164	0.277440
99	1	0	4.414665	1.300008	1.737479
100	8	0	3.702646	1.929525	1.493108
101	1	0	4.090805	2.839927	1.377566
102	1	0	-7.050129	1.132943	-0.689195
103	8	0	-7.383013	0.753358	-1.534220
104	1	0	-6.786901	1.114055	-2.222005

105	1	0	5.806990	2.936183	-1.863730
106	8	0	6.166629	3.662253	-1.321180
107	1	0	6.973823	3.279021	-0.912189
108	1	0	3.488343	4.672655	0.392248
109	8	0	4.371549	4.434409	0.735512
110	1	0	4.958385	4.287566	-0.042500
111	1	0	8.014109	1.241888	-0.431297
112	8	0	8.381916	2.149313	-0.340712
113	1	0	9.170667	2.175759	-0.901557
114	1	0	-4.613998	-4.607638	-2.610894
115	8	0	-3.991033	-5.373897	-2.622718
116	1	0	-4.305321	-5.929852	-3.350097
117	1	0	-8.005223	-1.213489	0.947414
118	8	0	-8.274874	-0.640282	1.706798
119	1	0	-9.178957	-0.355968	1.506968

SCF Done: E(RB3LYP) = -3021.46919438 A.U. after 1 cycles

Convg = 0.1651D-08 -V/T = 2.0092

Zero-point correction= 1.010357 (a.u.)

Thermal correction to Energy= 1.099222

Thermal correction to Enthalpy= 1.100167

Thermal correction to Gibbs Free Energy= 0.877552

Sum of electronic and zero-point Energies= -3020.458837

Sum of electronic and thermal Energies= -3020.369972

Sum of electronic and thermal Enthalpies= -3020.369028

Sum of electronic and thermal Free Energies= -3020.591642

===

[2] TS1(am) geometries in Figure 2. Hereafter, n denotes the number of water molecules around Ph-CO-NH-Et and OH-

n=3 TS

N-ethyl benzamide(C6H5-C(=O)-NHC2H5) and HO-(H2O)3, HO- addition TS1(am)

tuka03aa.chk Stoichiometry: C9H18NO5(1-)

Standard orientation:

Center Number	Atomic Number	Atomic Type	Coordinates (Angstroms) X	Y	Z
1	6	0	0.708812	-0.588882	-0.364345
2	6	0	-0.536797	0.266755	-0.537381
3	8	0	-0.536664	1.244188	-1.333203
4	8	0	-0.499883	1.066827	1.182326
5	1	0	-0.934259	1.893742	0.913961
6	6	0	1.764560	-0.424831	-1.266695
7	6	0	2.905266	-1.224411	-1.173991
8	6	0	0.811885	-1.561976	0.640443
9	7	0	-1.740404	-0.550894	-0.428235
10	6	0	-2.955327	0.059799	-0.970550
11	1	0	-3.164482	0.955563	-0.376369
12	1	0	-2.814370	0.399190	-2.006761
13	6	0	-4.133210	-0.909304	-0.878856
14	1	0	0.715493	2.667542	-1.135889
15	8	0	1.372784	3.361936	-0.903853
16	1	0	1.705173	3.067392	-0.031255
17	1	0	-2.332987	-0.944320	1.684526
18	8	0	-2.172292	-0.582537	2.575950
19	1	0	-1.529947	0.128586	2.313208
20	1	0	1.027121	1.665929	1.565115
21	8	0	1.875778	2.181075	1.749321
22	1	0	1.641483	2.773769	2.478227
23	1	0	-3.940600	-1.827837	-1.450952
24	1	0	-4.334097	-1.194868	0.159971
25	1	0	-5.041564	-0.451810	-1.289909
26	6	0	1.951390	-2.362074	0.729527
27	6	0	3.000347	-2.198981	-0.179250
28	1	0	1.677862	0.342642	-2.029225
29	1	0	3.722145	-1.082561	-1.878456
30	1	0	0.011125	-1.661563	1.366834
31	1	0	2.025778	-3.106032	1.519968
32	1	0	3.890470	-2.820593	-0.104586
33	1	0	-1.560869	-1.456984	-0.858505

SCF Done: E(RB+HF-LYP) = -784.753767799 A.U. after 1 cycles

Convg = 0.2480D-08 -V/T = 2.0095
S**2 = 0.0000
Zero-point correction= 0.271480 (a.u.)
Thermal correction to Energy= 0.291173
Thermal correction to Enthalpy= 0.292117
Thermal correction to Gibbs Free Energy= 0.222385
Sum of electronic and zero-point Energies= -784.482288
Sum of electronic and thermal Energies= -784.462595
Sum of electronic and thermal Enthalpies= -784.461651
Sum of electronic and thermal Free Energies= -784.531383

n=5 TS
N-ethyl benzamide and HO-(H2O)5 HO- addition TS1(am)
tuka05a.chk Stoichiometry C9H22NO7(1-)
Standard orientation:

Center Number	Atomic Number	Atomic Type	Coordinates (Angstroms) X	Y	Z
1	6	0	0.585987	-0.786118	-0.586881
2	6	0	-0.796340	-0.158222	-0.527575
3	8	0	-1.087206	0.797095	-1.310431
4	8	0	-0.574539	0.676786	1.173720
5	1	0	-1.249803	1.375895	1.089372
6	6	0	1.406892	-0.496239	-1.682068
7	6	0	2.667259	-1.085523	-1.797838
8	6	0	1.043266	-1.673626	0.398488
9	7	0	-1.805294	-1.138027	-0.222638
10	6	0	-3.181149	-0.795793	-0.583512
11	1	0	-3.461285	0.098139	-0.016886
12	1	0	-3.270321	-0.535158	-1.648405
13	6	0	-4.128486	-1.942516	-0.235422
14	1	0	-0.016743	2.373700	-1.266311
15	8	0	0.330022	3.264043	-1.029459
16	1	0	0.886502	3.081880	-0.243746
17	1	0	-1.930686	-1.643460	2.075721
18	8	0	-1.527600	-1.253434	2.870185
19	1	0	-1.114328	-0.452760	2.450785
20	1	0	0.756756	1.527089	1.309220
21	8	0	1.558820	2.169357	1.355835
22	1	0	1.459058	2.636176	2.198797
23	1	0	-3.860475	-2.861643	-0.774933
24	1	0	-4.109061	-2.162553	0.838023
25	1	0	-5.157586	-1.686572	-0.513261
26	6	0	2.302591	-2.264053	0.280287

27	6	0	3.118334	-1.974749	-0.818955
28	1	0	1.044363	0.199347	-2.432106
29	1	0	3.298894	-0.847490	-2.650318
30	1	0	0.426034	-1.866551	1.270747
31	1	0	2.653472	-2.940703	1.055780
32	1	0	4.099937	-2.434961	-0.907836
33	1	0	-1.529178	-2.053181	-0.569278
34	1	0	-2.286071	2.186671	-0.707098
35	8	0	-2.463287	3.017865	-0.217965
36	1	0	-1.617446	3.491488	-0.354601
37	1	0	3.220428	1.343512	1.289452
38	8	0	4.069785	0.847018	1.238899
39	1	0	3.836489	0.048509	0.739923

SCF Done: E(RB+HF-LYP) = -937.643011233 A.U. after 1 cycles
Convg = 0.2280D-08 -V/T = 2.0094
S**2 = 0.0000
Zero-point correction= 0.322152 (a.u.)
Thermal correction to Energy= 0.347208
Thermal correction to Enthalpy= 0.348152
Thermal correction to Gibbs Free Energy= 0.265947
Sum of electronic and zero-point Energies= -937.320859
Sum of electronic and thermal Energies= -937.295803
Sum of electronic and thermal Enthalpies= -937.294859
Sum of electronic and thermal Free Energies= -937.377064

n=8 TS
N-ethyl benzamide and HO-(H2O)8, HO- addition TS1(am)
tuka03cc.chk Stoichiometry: C9H28NO10(1-)
Standard orientation:

Center Number	Atomic Number	Atomic Type	Coordinates (Angstroms) X	Y	Z
1	6	0	-1.037207	-0.909048	-0.613492
2	6	0	-1.818637	0.360519	-0.286756
3	8	0	-1.375714	1.488666	-0.674703
4	8	0	-1.594693	0.358990	1.510894
5	1	0	-1.772361	1.302681	1.665436
6	6	0	-0.045803	-0.849282	-1.598436
7	6	0	0.670230	-1.994106	-1.957385
8	6	0	-1.311807	-2.136313	0.009151
9	7	0	-3.252419	0.109252	-0.403369
10	6	0	-4.086703	1.305174	-0.566950
11	1	0	-3.989788	1.903944	0.345090

12	1	0	-3.732335	1.935765	-1.393611
13	6	0	-5.548489	0.913486	-0.774224
14	1	0	0.144752	2.069968	-0.165648
15	8	0	1.024112	2.354373	0.222678
16	1	0	1.161032	1.731923	0.970112
17	1	0	-4.028993	-0.862092	1.350220
18	8	0	-3.867977	-1.061896	2.294001
19	1	0	-2.998267	-0.611446	2.377601
20	1	0	-0.068044	0.218457	1.916746
21	8	0	0.936137	0.256174	2.126177
22	1	0	1.017148	0.359946	3.085712
23	1	0	1.983556	-1.003198	1.407583
24	8	0	2.562898	-1.658995	0.947524
25	1	0	1.955560	-2.195625	0.411632
26	1	0	1.745988	4.207767	0.457775
27	8	0	2.446512	4.881122	0.343779
28	1	0	3.123883	4.384904	-0.147812
29	1	0	2.429822	2.286755	-0.778044
30	8	0	3.313907	2.371995	-1.231637
31	1	0	3.112342	2.474757	-2.173409
32	1	0	3.610779	-0.692576	-0.341565
33	8	0	4.335182	-0.315876	-0.884415
34	1	0	4.116129	0.634946	-0.979164
35	1	0	4.595736	-2.079970	1.393626
36	8	0	5.543666	-1.956719	1.205380
37	1	0	5.519643	-1.317411	0.465717
38	1	0	-5.672714	0.299604	-1.676971
39	1	0	-5.933412	0.342730	0.078292
40	1	0	-6.171055	1.807455	-0.897822
41	6	0	-0.595041	-3.280122	-0.347202
42	6	0	0.398023	-3.214074	-1.331303
43	1	0	0.158809	0.106643	-2.069865
44	1	0	1.447422	-1.931046	-2.714449
45	1	0	-2.069539	-2.183769	0.785226
46	1	0	-0.807352	-4.224037	0.149072
47	1	0	0.955474	-4.106013	-1.607510
48	1	0	-3.406925	-0.545899	-1.168231

SCF Done: E(RB+HF-LYP) = -1166.96917346 A.U. after 1 cycles

Convg = 0.3693D-08 -V/T = 2.0094

S**2 = 0.0000

Zero-point correction= 0.398417 (a.u.)

Thermal correction to Energy= 0.431309

Thermal correction to Enthalpy= 0.432253

Thermal correction to Gibbs Free Energy= 0.330715

Sum of electronic and zero-point Energies= -1166.570757

Sum of electronic and thermal Energies= -1166.537864
Sum of electronic and thermal Enthalpies= -1166.536920
Sum of electronic and thermal Free Energies= -1166.638458

n=12 TS
N-ethyl benzamide and HO-(H2O)12, HO- addtion TS, TS1(am)
tuka03dd.chk Stoichiometry C9H36NO14(1-)
Standard orientation:

Center Number	Atomic Number	Atomic Type	Coordinates (Angstroms) X	Y	Z
1	6	0	0.157672	-1.035726	-1.168732
2	6	0	-0.962084	-0.024549	-0.960683
3	8	0	-0.718930	1.215678	-1.183615
4	8	0	-1.071149	-0.152129	0.887198
5	1	0	-1.602918	0.640954	1.114640
6	6	0	1.258200	-0.663056	-1.949717
7	6	0	2.287236	-1.570362	-2.208860
8	6	0	0.095935	-2.340036	-0.655906
9	7	0	-2.224464	-0.556847	-1.374470
10	6	0	-3.265021	0.383662	-1.802075
11	1	0	-3.525004	1.018259	-0.950185
12	1	0	-2.901316	1.049655	-2.597563
13	6	0	-4.505509	-0.373504	-2.270727
14	1	0	0.593626	1.924783	-0.373116
15	8	0	1.293194	2.384295	0.181699
16	1	0	1.404495	1.779918	0.953599
17	1	0	-3.272489	-1.838256	0.332469
18	8	0	-2.991649	-1.939773	1.259739
19	1	0	-2.197298	-1.318456	1.261792
20	1	0	0.292155	0.057838	1.604569
21	8	0	1.210127	0.298464	2.014954
22	1	0	1.080485	0.364270	2.972611
23	1	0	2.594822	-0.731409	1.536360
24	8	0	3.368135	-1.257492	1.216579
25	1	0	2.989923	-1.916560	0.611607
26	1	0	0.561204	3.919155	0.578203
27	8	0	0.011989	4.742463	0.672424
28	1	0	0.534835	5.452073	0.271781
29	1	0	2.866984	2.647225	-0.598323
30	8	0	3.756373	2.798921	-1.007598
31	1	0	3.624424	2.674271	-1.958726
32	1	0	4.461559	-0.115697	0.123396
33	8	0	5.197657	0.411783	-0.252409

34	1	0	4.810220	1.291044	-0.448033
35	1	0	5.290315	-1.165324	2.177022
36	8	0	6.184676	-0.781410	2.210176
37	1	0	6.185596	-0.205535	1.419146
38	1	0	-4.274930	-1.039399	-3.113699
39	1	0	-4.933201	-0.978983	-1.463636
40	1	0	-5.273840	0.331329	-2.606186
41	6	0	1.124074	-3.248823	-0.915527
42	6	0	2.223585	-2.867162	-1.691910
43	1	0	1.295509	0.345711	-2.347925
44	1	0	3.142892	-1.262919	-2.803739
45	1	0	-0.739969	-2.643756	-0.035598
46	1	0	1.062470	-4.253053	-0.504570
47	1	0	3.024928	-3.574142	-1.892147
48	1	0	-1.735124	2.691809	-0.887553
49	8	0	-2.216245	3.469722	-0.512301
50	1	0	-1.508000	4.070530	-0.190125
51	1	0	-2.736641	2.683286	0.923791
52	8	0	-2.875280	2.082331	1.710646
53	1	0	-2.548350	2.575128	2.477613
54	1	0	-2.542819	-3.666458	1.521796
55	8	0	-2.270595	-4.610605	1.622730
56	1	0	-2.329997	-4.786020	2.572826
57	1	0	-4.308130	-0.730310	2.106990
58	8	0	-4.864399	0.032962	2.363199
59	1	0	-4.312488	0.806807	2.137732
60	1	0	-2.098434	-1.324197	-2.028092

SCF Done: E(RB+HF-LYP) = -1472.74204120   A.U. after   1 cycles

Convg =   0.2594D-08   -V/T = 2.0093

S**2   =   0.0000

Zero-point correction= 0.500557 (a.u.)

Thermal correction to Energy= 0.543788

Thermal correction to Enthalpy= 0.544732

Thermal correction to Gibbs Free Energy= 0.418220

Sum of electronic and zero-point Energies= -1472.241484

Sum of electronic and thermal Energies= -1472.198253

Sum of electronic and thermal Enthalpies= -1472.197309

Sum of electronic and thermal Free Energies= -1472.323821

n=16 TS

N-ethyl benzamide + HO-(H2O)16 TS1, TS1(am)

tuka02aa.chk   Stoichiometry:C9H44NO18(1-)

Standard orientation:

Center Number	Atomic Number	Atomic Type	Coordinates (Angstroms) X	Y	Z
1	6	0	-0.470652	2.399800	-0.844394
2	6	0	-0.180315	1.366931	0.254620
3	8	0	-1.118661	0.470879	0.459457
4	7	0	0.342700	2.010664	1.397317
5	6	0	0.645579	1.245170	2.603188
6	6	0	-1.799864	2.635945	-1.211927
7	6	0	-2.113841	3.598584	-2.173143
8	6	0	0.540684	3.151935	-1.458527
9	1	0	1.076941	2.683243	1.182820
10	6	0	0.885925	2.186439	3.783055
11	1	0	-0.202787	0.591747	2.814586
12	1	0	1.528264	0.597069	2.466522
13	8	0	1.157664	0.509965	-0.411976
14	1	0	0.896889	0.227086	-1.314410
15	1	0	1.587771	-0.852260	0.345443
16	8	0	1.952299	-1.713037	0.748674
17	1	0	2.700652	-1.434461	1.313807
18	1	0	2.706877	-2.407177	-0.567744
19	8	0	3.249789	-2.785737	-1.325315
20	1	0	3.390337	-2.037032	-1.927494
21	1	0	4.767183	-3.037333	-0.478314
22	8	0	5.544084	-3.023980	0.142972
23	1	0	5.564892	-3.901335	0.552826
24	1	0	1.612379	-3.375996	-2.394466
25	8	0	0.918694	-3.204272	-3.058986
26	1	0	0.050873	-3.504101	-2.664685
27	1	0	0.661174	-1.532679	-3.126138
28	8	0	0.461399	-0.551175	-3.066459
29	1	0	0.752093	-0.153017	-3.899660
30	1	0	5.065508	-1.649753	1.162312
31	8	0	4.574746	-0.895313	1.578999
32	1	0	5.059210	-0.636911	2.376590
33	1	0	-1.776876	-0.345121	-0.967672
34	8	0	-2.108694	-0.773240	-1.803528
35	1	0	-1.432968	-0.526168	-2.465828
36	1	0	-1.815175	-2.628230	-2.039072
37	8	0	-1.576407	-3.579154	-2.121011
38	1	0	-1.869970	-3.937887	-1.248433
39	1	0	-3.831575	-1.515954	-1.181114
40	8	0	-4.464323	-2.010375	-0.624051
41	1	0	-4.860708	-1.346189	-0.014687
42	1	0	-0.847662	-1.064812	1.165701
43	8	0	-0.733012	-1.960657	1.595774

44	1	0	0.223355	-2.144736	1.488287
45	1	0	-1.656867	-1.836059	3.101483
46	8	0	-2.343048	-1.621517	3.782176
47	1	0	-3.046979	-2.270386	3.628954
48	1	0	-1.944807	-3.214478	0.852215
49	8	0	-2.601781	-3.826397	0.454936
50	1	0	-3.353363	-3.247451	0.179614
51	1	0	2.816465	1.024139	-0.349095
52	8	0	3.769296	1.284433	-0.214281
53	1	0	4.141875	0.590024	0.364135
54	1	0	3.527289	2.751943	0.736901
55	8	0	3.220396	3.527803	1.273584
56	1	0	3.423512	4.310456	0.741796
57	1	0	-2.912549	-0.008539	2.964230
58	8	0	-3.161896	0.718587	2.350330
59	1	0	-2.434337	0.745699	1.680167
60	1	0	-4.616935	0.249836	1.616685
61	8	0	-5.448266	-0.072571	1.162537
62	1	0	-5.847773	0.712152	0.760954
63	6	0	0.227862	4.115941	-2.418266
64	6	0	-1.101764	4.343406	-2.779777
65	1	0	-2.583159	2.052290	-0.741269
66	1	0	-3.152852	3.764280	-2.447800
67	1	0	1.577652	2.972046	-1.194422
68	1	0	1.026415	4.686340	-2.887611
69	1	0	-1.345600	5.092438	-3.529757
70	1	0	1.720521	2.865998	3.576164
71	1	0	-0.009187	2.785962	3.980437
72	1	0	1.125183	1.611812	4.686197

SCF Done: E(RB+HF-LYP) = -1778.52641035   A.U. after   1 cycles
Convg =   0.4358D-08   -V/T = 2.0093
S**2   =   0.0000

Zero-point correction=   0.608558 (a.u.)
Thermal correction to Energy=   0.659034
Thermal correction to Enthalpy=   0.659978
Thermal correction to Gibbs Free Energy=   0.521684
Sum of electronic and zero-point Energies=   -1777.917853
Sum of electronic and thermal Energies=   -1777.867376
Sum of electronic and thermal Enthalpies=   -1777.866432
Sum of electronic and thermal Free Energies=   -1778.004727

n=24 TS

N-ethyl benzamide and HO-(H2O)24 TS1(am)

tuka02hh.chk   Stoichiometry C9H60NO26(1-)

Standard orientation:

Center Number	Atomic Number	Atomic Type	Coordinates (Angstroms) X	Y	Z
1	6	0	0.738258	-2.509405	-1.715082
2	6	0	0.594643	-1.699462	-0.426315
3	8	0	1.570108	-0.877410	-0.130417
4	7	0	0.103841	-2.494532	0.616265
5	6	0	-0.143069	-1.947900	1.950257
6	6	0	2.007121	-2.660926	-2.284112
7	6	0	2.181726	-3.427656	-3.437988
8	6	0	-0.355031	-3.144732	-2.321755
9	1	0	-0.613125	-3.145510	0.314979
10	6	0	-0.407308	-3.072920	2.947823
11	1	0	0.742337	-1.388943	2.258055
12	1	0	-0.988243	-1.240491	1.943996
13	8	0	-0.781220	-0.650915	-0.859048
14	1	0	-0.576717	-0.264679	-1.736238
15	1	0	-1.087316	0.620945	0.074125
16	8	0	-1.361981	1.442795	0.614372
17	1	0	-2.066870	1.135496	1.216455
18	1	0	-2.149021	2.399507	-0.557720
19	8	0	-2.659783	2.965456	-1.205951
20	1	0	-2.955561	2.351807	-1.898076
21	1	0	-4.163798	3.237811	-0.208607
22	8	0	-4.914690	3.072197	0.412580
23	1	0	-4.716337	3.565598	1.235706
24	1	0	-1.097528	3.562183	-2.261258
25	8	0	-0.400074	3.461938	-2.939667
26	1	0	0.458973	3.691662	-2.506759
27	1	0	-0.266921	1.801460	-3.296016
28	8	0	-0.130682	0.813414	-3.391166
29	1	0	-0.400437	0.576619	-4.290773
30	1	0	-4.392196	1.508496	1.126124
31	8	0	-3.984127	0.812970	1.701040
32	1	0	-4.741238	0.247948	1.987873
33	1	0	2.090146	0.178309	-1.396954
34	8	0	2.385434	0.777147	-2.138122
35	1	0	1.692183	0.665341	-2.822586
36	1	0	2.235303	2.578685	-1.938139
37	8	0	2.155527	3.562507	-1.883697
38	1	0	2.468600	3.754987	-0.974071
39	1	0	4.159941	1.175235	-1.693977
40	8	0	4.890883	1.571106	-1.172531
41	1	0	5.365097	0.810758	-0.759734

42	1	0	1.360421	0.502979	0.857924
43	8	0	1.316054	1.328690	1.417338
44	1	0	0.380592	1.612765	1.327253
45	1	0	2.290103	0.859139	2.906188
46	8	0	3.059008	0.511615	3.413806
47	1	0	3.677692	1.278609	3.469776
48	1	0	2.636211	2.436175	0.869083
49	8	0	3.383472	3.044130	0.647894
50	1	0	3.992890	2.522253	0.063980
51	1	0	-2.457100	-1.037366	-0.736288
52	8	0	-3.432660	-1.081385	-0.520621
53	1	0	-3.543076	-0.463984	0.230614
54	1	0	-4.803207	-2.429323	0.022137
55	8	0	-5.772283	-2.544561	0.117012
56	1	0	-6.093812	-2.079037	-0.695677
57	1	0	3.567158	-0.719878	2.218929
58	8	0	3.697086	-1.436974	1.544002
59	1	0	2.924033	-1.350156	0.926139
60	1	0	5.140353	-1.008059	0.634627
61	8	0	5.909995	-0.701307	0.086764
62	1	0	6.170616	-1.458297	-0.457241
63	6	0	-0.180496	-3.915172	-3.471377
64	6	0	1.089731	-4.058341	-4.034605
65	1	0	2.853572	-2.168139	-1.818501
66	1	0	3.174166	-3.530627	-3.869460
67	1	0	-1.351327	-3.014993	-1.908966
68	1	0	-1.040118	-4.395826	-3.931614
69	1	0	1.225097	-4.654483	-4.933617
70	1	0	-1.299388	-3.651537	2.670855
71	1	0	0.449876	-3.751639	2.993534
72	1	0	-0.566608	-2.658265	3.948104
73	1	0	-6.474175	1.996705	-0.051040
74	8	0	-7.047551	1.220321	-0.209643
75	1	0	-6.994931	0.684482	0.606675
76	1	0	-3.876564	2.363983	2.951024
77	8	0	-4.075970	3.311804	3.103375
78	1	0	-3.289205	3.692816	3.520553
79	1	0	3.962371	3.980391	-2.799560
80	8	0	4.914729	3.914056	-3.004513
81	1	0	5.213318	3.181548	-2.433777
82	1	0	2.527482	-0.761728	4.831879
83	8	0	2.251030	-1.556837	5.327895
84	1	0	2.546784	-2.299258	4.763522
85	1	0	4.207175	3.049694	2.212505
86	8	0	4.551482	2.853297	3.123880
87	1	0	4.356822	3.635756	3.659389

88	1	0	-6.168502	-1.448665	1.307970
89	8	0	-6.320697	-0.660580	1.919117
90	1	0	-6.798493	-0.989496	2.695009
91	1	0	-4.989381	-0.663008	-1.728971
92	8	0	-5.941282	-0.718363	-1.951647
93	1	0	-6.361277	0.040341	-1.484060
94	1	0	3.469268	-2.857466	2.661140
95	8	0	3.346017	-3.495274	3.405449
96	1	0	4.240946	-3.707518	3.708641

SCF Done: E(RB+HF-LYP) = -2390.06356594 A.U. after 1 cycles
Convg = 0.5806D-08 -V/T = 2.0093
S**2 = 0.0000

Zero-point correction= 0.816166 (a.u.)
Thermal correction to Energy= 0.885905
Thermal correction to Enthalpy= 0.886849
Thermal correction to Gibbs Free Energy= 0.706041
Sum of electronic and zero-point Energies= -2389.247400
Sum of electronic and thermal Energies= -2389.177661
Sum of electronic and thermal Enthalpies= -2389.176717
Sum of electronic and thermal Free Energies= -2389.357525

n=32 TS

N-ethyl benzamide and HO-(H2O)32 TS1(am)

tuka02ww.chk Stoichiometry: C9H76NO34(1-)

Standard orientation:

Center Number	Atomic Number	Atomic Type	Coordinates (Angstroms) X	Y	Z
1	6	0	-0.833024	-0.878535	2.940360
2	6	0	-0.976524	-1.087997	1.431556
3	8	0	-1.844492	-0.320763	0.810826
4	7	0	-1.027926	-2.458438	1.128574
5	6	0	-1.234583	-2.946187	-0.233602
6	6	0	-1.888653	-0.288664	3.643804
7	6	0	-1.816955	-0.127597	5.028610
8	6	0	0.293802	-1.314104	3.651610
9	1	0	-0.360418	-3.007762	1.658779
10	6	0	-1.432565	-4.460543	-0.229213
11	1	0	-2.128723	-2.462462	-0.632956
12	1	0	-0.395952	-2.678488	-0.898026
13	8	0	0.585530	-0.560251	0.857661
14	1	0	0.687228	0.366715	1.177956
15	1	0	0.781550	-0.396100	-0.716540

16	8	0	1.012333	-0.188019	-1.690256
17	1	0	1.488497	-0.962872	-2.031600
18	1	0	2.173747	1.076515	-1.357837
19	8	0	2.879813	1.757760	-1.215869
20	1	0	3.155433	1.711500	-0.261194
21	1	0	4.181737	1.274257	-2.085151
22	8	0	5.001999	0.856213	-2.491266
23	1	0	4.796508	0.643921	-3.427166
24	1	0	1.889755	3.353952	-0.937201
25	8	0	1.559155	4.060430	-0.343432
26	1	0	0.642448	4.275653	-0.637028
27	1	0	1.130128	2.895389	1.049311
28	8	0	1.010941	2.212414	1.752955
29	1	0	1.929729	2.085480	2.066869
30	1	0	4.237327	-0.868759	-2.032396
31	8	0	3.808102	-1.752528	-2.073510
32	1	0	4.544060	-2.379892	-1.882180
33	1	0	-1.791160	1.387047	1.166166
34	8	0	-1.763594	2.361171	1.378762
35	1	0	-0.962447	2.452428	1.932596
36	1	0	-1.380459	3.553434	0.024200
37	8	0	-1.201508	4.218928	-0.683957
38	1	0	-1.767892	3.916208	-1.422358
39	1	0	-3.511901	2.979703	1.123112
40	8	0	-4.274695	3.239775	0.561274
41	1	0	-5.031646	2.671021	0.829120
42	1	0	-1.739802	0.024083	-0.834330
43	8	0	-1.755243	0.297295	-1.799503
44	1	0	-0.818586	0.216757	-2.077436
45	1	0	-3.120965	-0.633193	-2.546260
46	8	0	-4.056248	-0.935281	-2.637458
47	1	0	-4.525561	-0.186380	-3.066451
48	1	0	-2.707263	1.852631	-1.853296
49	8	0	-3.288370	2.644615	-1.958950
50	1	0	-3.691915	2.824536	-1.068359
51	1	0	2.081331	-1.351721	0.897169
52	8	0	2.985675	-1.762596	0.736632
53	1	0	3.086982	-1.805028	-0.237245
54	1	0	4.229409	-3.008362	1.475174
55	8	0	5.183298	-3.193659	1.631927
56	1	0	5.508055	-2.328094	1.968573
57	1	0	-4.330662	-0.803808	-0.823854
58	8	0	-4.454849	-0.814606	0.155151
59	1	0	-3.538833	-0.761502	0.534096
60	1	0	-5.485750	0.501971	0.706425
61	8	0	-6.149928	1.213549	0.897622

62	1	0	-6.850617	0.767090	1.424156
63	6	0	0.364285	-1.157093	5.035979
64	6	0	-0.691085	-0.561002	5.729209
65	1	0	-2.765088	0.044819	3.099111
66	1	0	-2.644493	0.337546	5.557798
67	1	0	1.130467	-1.755920	3.118141
68	1	0	1.250126	-1.490841	5.570496
69	1	0	-0.633398	-0.431941	6.807043
70	1	0	-0.529197	-4.979778	0.119586
71	1	0	-2.261733	-4.734769	0.430912
72	1	0	-1.672556	-4.825538	-1.232502
73	1	0	6.255670	0.130455	-1.424705
74	8	0	6.840452	-0.264038	-0.735530
75	1	0	6.862922	-1.228007	-0.922534
76	1	0	3.816914	-1.304763	-4.024203
77	8	0	4.106392	-0.656216	-4.699529
78	1	0	3.355493	-0.522909	-5.296640
79	1	0	-2.539030	5.680379	-0.060437
80	8	0	-3.396398	6.052913	0.219823
81	1	0	-3.934537	5.257925	0.392915
82	1	0	-4.997285	-2.585313	-2.735552
83	8	0	-5.603348	-3.347395	-2.624337
84	1	0	-6.198690	-3.079335	-1.893847
85	1	0	-4.561407	2.038449	-2.909071
86	8	0	-5.251271	1.495495	-3.394293
87	1	0	-5.386335	1.916259	-4.256019
88	1	0	5.756673	-3.047089	-0.065667
89	8	0	6.087658	-2.981643	-0.997819
90	1	0	6.549369	-3.832248	-1.113752
91	1	0	4.407829	-0.689625	1.293108
92	8	0	5.274157	-0.379941	1.649285
93	1	0	5.852303	-0.253742	0.862897
94	1	0	-6.077827	-1.790753	-0.053728
95	8	0	-6.972609	-1.922988	-0.434951
96	1	0	-7.114508	-1.115153	-0.992178
97	1	0	6.168764	-4.784550	1.190766
98	8	0	6.701957	-5.308309	0.557642
99	1	0	6.882344	-6.160174	0.979228
100	1	0	4.477517	1.386155	1.687708
101	8	0	3.759602	1.987918	1.399755
102	1	0	4.134264	2.897303	1.234680
103	1	0	-6.984504	1.030619	-0.769107
104	8	0	-7.316293	0.593695	-1.586738
105	1	0	-6.728247	0.919735	-2.298257
106	1	0	5.841696	2.781386	-2.011199
107	8	0	6.198535	3.523438	-1.490234

108	1	0	6.993535	3.147901	-1.051096
109	1	0	3.507718	4.654927	0.148984
110	8	0	4.395095	4.446738	0.501990
111	1	0	4.978543	4.250048	-0.267001
112	1	0	8.022422	1.126947	-0.462587
113	8	0	8.382562	2.039939	-0.406176
114	1	0	9.186833	2.045694	-0.945147
115	1	0	-4.617525	-4.863445	-2.487079
116	8	0	-4.080019	-5.689465	-2.547463
117	1	0	-4.458454	-6.163991	-3.301517
118	1	0	-7.867566	-1.223989	1.032024
119	8	0	-8.153797	-0.610321	1.753285
120	1	0	-9.059864	-0.353283	1.527145

SCF Done: E(RB+HF-LYP) = -3001.59879182 A.U. after 1 cycles

Convg = 0.4028D-08 -V/T = 2.0093

S**2 = 0.0000

Zero-point correction= 1.023828 (a.u.)

Thermal correction to Energy= 1.112687

Thermal correction to Enthalpy= 1.113631

Thermal correction to Gibbs Free Energy= 0.891095

Sum of electronic and zero-point Energies= -3000.574964

Sum of electronic and thermal Energies= -3000.486105

Sum of electronic and thermal Enthalpies= -3000.485160

Sum of electronic and thermal Free Energies= -3000.707697

===

[3] Geometries of the n=16 ester hydrolysis in Figure 3

(3-1) reactant-like complex (precursor)
zuka02aa.for.g09.chk Stoichiometry C9H43O19(1-)
Standard orientation:

Center Number	Atomic Number	Atomic Type	Coordinates (Angstroms) X	Y	Z
1	6	0	-3.459320	-1.804560	0.269734
2	6	0	-2.283186	-1.463164	-0.575967
3	8	0	-1.787277	-0.333465	-0.624450
4	8	0	-1.825966	-2.489807	-1.284015
5	6	0	-0.640002	-2.277620	-2.118665
6	6	0	-3.927062	-0.854667	1.189895
7	6	0	-5.027542	-1.147187	1.992776
8	6	0	-4.101807	-3.047369	0.162822
9	6	0	-0.415874	-3.552816	-2.904888
10	1	0	-0.833317	-1.419907	-2.768374
11	1	0	0.202810	-2.057319	-1.456905
12	8	0	2.076165	-1.796150	-0.184243
13	1	0	1.681141	-1.360514	0.602432
14	1	0	2.268714	-1.038424	-0.827995
15	8	0	2.614487	0.294854	-1.742392
16	1	0	2.896190	0.073593	-2.642200
17	1	0	3.836438	0.681503	-0.817596
18	8	0	4.588474	0.797383	-0.126145
19	1	0	4.608672	-0.074901	0.335457
20	1	0	6.065693	0.619315	-0.936853
21	8	0	6.912396	0.304427	-1.377402
22	1	0	6.773283	0.435874	-2.326643
23	1	0	3.665902	1.567905	1.251537
24	8	0	3.135639	1.647849	2.081604
25	1	0	2.541874	2.433663	1.985131
26	1	0	1.921115	0.395666	2.055002
27	8	0	1.303490	-0.370551	2.218764
28	1	0	1.812276	-0.865812	2.895851
29	1	0	6.976068	-1.388456	-0.864211
30	8	0	6.918778	-2.303198	-0.482413
31	1	0	7.660959	-2.367700	0.136419
32	1	0	-1.130036	0.805365	1.006731
33	8	0	-0.979457	1.136625	1.910816
34	1	0	-0.273683	0.535819	2.254802
35	1	0	0.344328	2.767015	2.031655
36	8	0	1.044765	3.436797	1.905043
37	1	0	0.801114	3.838531	1.040065

38	1	0	-2.118464	2.779205	1.681139
39	8	0	-2.431985	3.559127	1.186870
40	1	0	-3.300531	3.322741	0.792255
41	1	0	-0.240093	0.748692	-1.446701
42	8	0	0.328011	1.461773	-1.785521
43	1	0	1.284443	1.055652	-1.798075
44	1	0	-0.618570	2.515831	-2.988439
45	8	0	-1.304715	3.191459	-3.202563
46	1	0	-1.131196	3.863182	-2.512426
47	1	0	-0.003139	3.038724	-0.849627
48	8	0	-0.260908	3.945570	-0.559642
49	1	0	-1.086414	3.845516	-0.027336
50	1	0	3.704684	-2.012179	0.503529
51	8	0	4.550041	-1.808395	0.987915
52	1	0	5.303203	-2.182728	0.478896
53	1	0	4.149656	-1.327511	2.777341
54	8	0	3.744257	-0.904473	3.565611
55	1	0	3.803764	0.051118	3.374802
56	1	0	-2.725928	2.260990	-2.665366
57	8	0	-3.381013	1.641465	-2.244724
58	1	0	-2.831540	1.019213	-1.734709
59	1	0	-4.312069	2.461639	-1.044899
60	8	0	-4.747661	2.891569	-0.258847
61	1	0	-5.491484	2.321255	-0.019267
62	6	0	-5.206572	-3.330135	0.962581
63	6	0	-5.670450	-2.381788	1.877857
64	1	0	-3.412622	0.094633	1.286780
65	1	0	-5.376935	-0.412724	2.712802
66	1	0	-3.733656	-3.779869	-0.546490
67	1	0	-5.704504	-4.291852	0.873961
68	1	0	-6.529595	-2.607112	2.504558
69	1	0	-0.243025	-4.399383	-2.232937
70	1	0	-1.268672	-3.782271	-3.553311
71	1	0	0.473850	-3.431009	-3.531782

SCF Done: E(RB3LYP) = -1798.42467109 A.U. after 1 cycles
Convg = 0.2884D-08 -V/T = 2.0093
Zero-point correction= 0.596344 (a.u.)
Thermal correction to Energy= 0.647470
Thermal correction to Enthalpy= 0.648414
Thermal correction to Gibbs Free Energy= 0.506284
Sum of electronic and zero-point Energies= -1797.828327
Sum of electronic and thermal Energies= -1797.777202
Sum of electronic and thermal Enthalpies= -1797.776257
Sum of electronic and thermal Free Energies= -1797.918387

(3-2) TS1(es) which is also shown in n=16 of Figure 1

ethyl benzoate + HO-(H2O)16 TS1(es)

zuka02aa.chk Stoichiometry C9H43O19(1-)

Standard orientation:

Center Number	Atomic Number	Atomic Type	Coordinates (Angstroms) X	Y	Z
1	6	0	-0.029063	2.670394	-0.007977
2	6	0	-0.333003	1.442850	0.812460
3	8	0	-1.376670	0.754484	0.605280
4	8	0	0.171988	1.585091	2.066932
5	6	0	-0.139275	0.572461	3.039007
6	6	0	-1.002168	3.171408	-0.877904
7	6	0	-0.749097	4.321731	-1.629854
8	6	0	1.198353	3.337491	0.111139
9	6	0	0.566257	0.950146	4.329589
10	1	0	-1.223399	0.524903	3.180571
11	1	0	0.203927	-0.397340	2.667420
12	8	0	1.112393	0.292708	0.071213
13	1	0	0.932669	0.320348	-0.890057
14	1	0	1.114067	-1.184518	0.519889
15	8	0	1.192987	-2.180322	0.801716
16	1	0	1.845657	-2.191722	1.527157
17	1	0	2.282543	-2.529205	-0.575781
18	8	0	3.048589	-2.485507	-1.207847
19	1	0	3.408064	-1.583854	-1.018809
20	1	0	4.170642	-3.491734	-0.329493
21	8	0	4.696286	-3.933476	0.394713
22	1	0	4.341013	-4.832240	0.461187
23	1	0	2.010705	-2.321772	-2.810826
24	8	0	1.402411	-2.060926	-3.535093
25	1	0	0.524611	-2.466652	-3.319601
26	1	0	1.016843	-0.447951	-3.233872
27	8	0	0.682451	0.445165	-2.919219
28	1	0	1.211674	1.125550	-3.359063
29	1	0	4.222380	-2.794697	1.660728
30	8	0	3.807768	-2.051158	2.172094
31	1	0	4.304338	-1.966378	2.999044
32	1	0	-1.780425	0.266555	-1.176166
33	8	0	-1.957116	0.073115	-2.124303
34	1	0	-1.149027	0.394229	-2.581711
35	1	0	-1.491720	-1.730337	-2.649809
36	8	0	-1.210496	-2.637471	-2.894836
37	1	0	-1.749771	-3.194595	-2.285945
38	1	0	-3.752133	-0.711160	-2.126512

39	8	0	-4.520913	-1.258597	-1.870622
40	1	0	-5.010333	-0.733016	-1.199075
41	1	0	-1.479635	-1.082881	0.892639
42	8	0	-1.537605	-2.041667	1.114426
43	1	0	-0.594979	-2.336076	1.095771
44	1	0	-2.722830	-2.085136	2.424479
45	8	0	-3.492898	-1.889416	3.015905
46	1	0	-4.215209	-2.436026	2.670742
47	1	0	-2.530443	-3.008100	-0.197370
48	8	0	-3.023201	-3.456601	-0.916608
49	1	0	-3.672187	-2.781392	-1.227886
50	1	0	2.757130	0.240645	0.083527
51	8	0	3.733753	-0.035893	0.041959
52	1	0	3.882110	-0.538171	0.868944
53	1	0	4.855338	1.390809	-0.064081
54	8	0	5.487433	2.140200	-0.180177
55	1	0	5.722263	2.112831	-1.118899
56	1	0	-3.796555	-0.141584	2.455045
57	8	0	-3.878579	0.723874	1.990145
58	1	0	-3.022635	0.840248	1.523462
59	1	0	-5.125077	0.487778	0.813224
60	8	0	-5.812308	0.299351	0.117364
61	1	0	-6.118306	1.164679	-0.190050
62	6	0	1.449119	4.483124	-0.640012
63	6	0	0.475828	4.978745	-1.514168
64	1	0	-1.953836	2.658323	-0.964538
65	1	0	-1.512870	4.701300	-2.304012
66	1	0	1.955900	2.940920	0.777266
67	1	0	2.408934	4.984545	-0.546915
68	1	0	0.674130	5.872185	-2.101826
69	1	0	1.650787	0.994343	4.183860
70	1	0	0.226743	1.928073	4.686954
71	1	0	0.347041	0.204541	5.102382

SCF Done: E(RB+HF-LYP) = -1798.39889531   A.U. after   1 cycles

Convg =   0.2155D-08   -V/T = 2.0093

S**2   =   0.0000

Zero-point correction=   0.595360 (a.u.)

Thermal correction to Energy=   0.646200

Thermal correction to Enthalpy=   0.647145

Thermal correction to Gibbs Free Energy=   0.506737

Sum of electronic and zero-point Energies=   -1797.803536

Sum of electronic and thermal Energies=   -1797.752695

Sum of electronic and thermal Enthalpies=   -1797.751751

Sum of electronic and thermal Free Energies=   -1797.892158

(3-3) Anionic tetrahedral intermediate, Ph-C(-O-)(OH)OEt and (H2O)16, Int1(es)

zukaphxx.rev.chk Stoichiometry C9H43O19(1-)

Standard orientation:

Center Number	Atomic Number	Atomic Type	Coordinates (Angstroms) X	Y	Z
1	6	0	-3.111915	-0.725212	-0.378531
2	6	0	-1.576014	-0.782626	-0.487489
3	8	0	-0.945002	0.206886	0.156154
4	8	0	-1.221740	-0.830325	-1.885831
5	1	0	-0.732841	-0.010069	-2.113484
6	6	0	-3.730511	0.009293	0.637318
7	6	0	-5.123175	0.034248	0.753237
8	6	0	-3.913865	-1.443990	-1.275576
9	8	0	-1.137670	-2.111359	-0.052893
10	6	0	-1.322683	-2.409897	1.328027
11	1	0	-2.391551	-2.375426	1.583352
12	6	0	-0.757158	-3.797001	1.594879
13	1	0	-0.805651	-1.665519	1.946612
14	1	0	0.675680	0.029075	0.058930
15	8	0	1.683714	-0.060530	0.020282
16	1	0	2.021972	0.654192	-0.572520
17	1	0	2.132218	-1.496278	-0.871309
18	8	0	2.558599	-2.267743	-1.332570
19	1	0	1.812192	-2.685543	-1.835752
20	1	0	3.998168	-1.181151	-1.971487
21	8	0	4.619630	-0.422791	-2.039773
22	1	0	5.035071	-0.356754	-1.150688
23	1	0	3.343172	-1.835876	1.807950
24	8	0	3.279182	-0.871825	2.023269
25	1	0	2.650025	-0.513596	1.335196
26	1	0	2.248112	-0.578918	3.432133
27	8	0	1.566646	-0.327865	4.108210
28	1	0	2.018655	0.267892	4.723496
29	1	0	3.490891	0.963351	-2.091341
30	8	0	2.752764	1.602867	-1.910900
31	1	0	2.032721	1.412043	-2.554532
32	1	0	-0.600645	0.678743	1.837811
33	8	0	-0.319804	1.179633	2.646924
34	1	0	0.265901	0.588558	3.174339
35	1	0	0.582157	2.629089	1.997402
36	8	0	0.718231	3.538923	1.630082
37	1	0	0.070180	4.065969	2.135594
38	1	0	-1.632247	2.653029	2.992915

39	8	0	-2.034693	3.511422	2.750960
40	1	0	-2.241088	3.392711	1.801807
41	1	0	-1.437233	1.885258	-0.094453
42	8	0	-1.513928	2.878780	-0.079418
43	1	0	-0.674611	3.154111	0.347674
44	1	0	-1.011030	3.495581	-1.734358
45	8	0	-0.415272	3.715019	-2.491448
46	1	0	0.410889	4.024272	-2.044068
47	1	0	4.797175	-0.320928	1.218549
48	8	0	5.569548	-0.081045	0.646423
49	1	0	5.705553	0.868634	0.781461
50	1	0	-0.166470	-3.576720	-1.595083
51	8	0	0.183463	-3.184297	-2.413004
52	1	0	-0.286059	-2.314198	-2.390678
53	1	0	3.144510	-3.142376	0.051664
54	8	0	3.491605	-3.433606	0.939375
55	1	0	4.421265	-3.661299	0.789917
56	1	0	0.109798	1.089196	-4.134283
57	8	0	0.296835	1.258880	-3.199005
58	1	0	-0.019940	2.199291	-3.010603
59	1	0	2.392829	3.327855	-1.243030
60	8	0	1.919347	4.143427	-0.971531
61	1	0	1.639781	3.991614	-0.045836
62	6	0	-5.303071	-1.421313	-1.161580
63	6	0	-5.914145	-0.680477	-0.145641
64	1	0	-3.116298	0.568798	1.334651
65	1	0	-5.585249	0.617008	1.546450
66	1	0	-3.438299	-2.010193	-2.069832
67	1	0	-5.911614	-1.978884	-1.870326
68	1	0	-6.998208	-0.659450	-0.059163
69	1	0	-0.887902	-4.059991	2.651217
70	1	0	-1.271941	-4.551369	0.987883
71	1	0	0.313544	-3.835493	1.365509

SCF Done: E(RB+HF-LYP) = -1798.42108073   A.U. after   1 cycles

Convg =   0.1330D-08   -V/T = 2.0093

S**2   =   0.0000

Zero-point correction=   0.600051 (a.u.)

Thermal correction to Energy=   0.649207

Thermal correction to Enthalpy=   0.650151

Thermal correction to Gibbs Free Energy=   0.515271

Sum of electronic and zero-point Energies=   -1797.821030

Sum of electronic and thermal Energies=   -1797.771874

Sum of electronic and thermal Enthalpies=   -1797.770929

Sum of electronic and thermal Free Energies=   -1797.905810

(3-4) TS2'(es),

formation of the neutral tetrahedral intermediate Int2'(es)

zuka07.g09.chk Stoichiometry C9H43O19(1-)

Standard orientation:

Center Number	Atomic Number	Atomic Type	Coordinates (Angstroms) X	Y	Z
1	6	0	2.555055	-0.970578	0.235205
2	6	0	1.227882	-0.783265	-0.515696
3	8	0	0.413977	0.062830	0.228759
4	8	0	0.533823	-2.024998	-0.638932
5	6	0	1.178608	-3.053926	-1.418704
6	6	0	2.533033	-1.351652	1.584195
7	6	0	3.722602	-1.547373	2.283201
8	6	0	3.787924	-0.805534	-0.401950
9	6	0	0.246492	-4.251973	-1.486423
10	1	0	1.386978	-2.665482	-2.419955
11	1	0	2.131314	-3.320039	-0.942933
12	8	0	1.411337	-0.316771	-1.829095
13	1	0	1.761716	0.612026	-1.835068
14	1	0	-1.777450	1.599943	-2.223302
15	8	0	-1.598654	1.834067	-3.161625
16	1	0	-1.308499	0.977582	-3.553117
17	1	0	-0.298336	3.020613	-3.147124
18	8	0	0.456511	3.648370	-2.987828
19	1	0	0.171581	4.188643	-2.205685
20	1	0	1.651193	2.803048	-2.421183
21	8	0	2.395890	2.249507	-1.979159
22	1	0	3.185936	2.370010	-2.526220
23	1	0	1.103317	1.807659	0.598097
24	8	0	1.542555	2.682886	0.681264
25	1	0	2.139342	2.694342	-0.095367
26	1	0	0.469941	4.204054	-0.052908
27	8	0	-0.126532	4.797058	-0.554367
28	1	0	-1.014932	4.514212	-0.232836
29	1	0	0.481260	3.298046	2.254706
30	8	0	-0.345120	3.408928	2.761229
31	1	0	-0.321438	2.736296	3.478812
32	1	0	-0.531131	0.223854	-0.215418
33	8	0	-1.945989	0.603759	-0.564615
34	1	0	-2.301052	-0.123247	-1.121016
35	1	0	-2.613063	0.518277	0.451015
36	8	0	-3.206903	0.419887	1.513819
37	1	0	-3.491425	1.319428	1.743899
38	1	0	-2.121959	2.568589	0.187768

39	8	0	-2.224944	3.403341	0.682048
40	1	0	-1.653992	3.322876	1.484156
41	1	0	0.020459	-0.700075	-3.102361
42	8	0	-0.721635	-0.789960	-3.734211
43	1	0	-1.427407	-1.245495	-3.228501
44	1	0	-1.907334	-0.118995	2.365362
45	8	0	-1.023220	-0.485633	2.694796
46	1	0	-0.410858	-0.269494	1.959577
47	1	0	-0.577764	0.579331	3.999679
48	8	0	-0.315440	1.298216	4.635315
49	1	0	0.551586	1.042681	4.981867
50	6	0	4.981095	-0.997827	0.300207
51	6	0	4.952093	-1.366669	1.644127
52	1	0	1.582287	-1.504318	2.084773
53	1	0	3.688965	-1.842870	3.328709
54	1	0	3.812189	-0.530975	-1.451080
55	1	0	5.932242	-0.860503	-0.208200
56	1	0	5.879714	-1.516168	2.191067
57	1	0	0.044213	-4.660405	-0.491034
58	1	0	-0.707030	-3.976438	-1.947911
59	1	0	0.708655	-5.039093	-2.093735
60	1	0	-1.235267	-2.283844	2.078360
61	8	0	-1.161430	-3.052670	1.470295
62	1	0	-0.569271	-2.719985	0.765066
63	1	0	-4.453370	-0.680269	1.440930
64	8	0	-5.125998	-1.426798	1.378582
65	1	0	-5.304238	-1.689564	2.293195
66	1	0	-3.778062	-1.408312	-2.532918
67	8	0	-2.927316	-1.637004	-2.129822
68	1	0	-3.140646	-2.276763	-1.382669
69	1	0	-4.172381	-2.662534	0.468176
70	8	0	-3.527711	-3.211886	-0.041134
71	1	0	-2.720278	-3.238879	0.527796

SCF Done: E(RB3LYP) = -1798.41015057   A.U. after   1 cycles
Convg =   0.2134D-08   -V/T = 2.0092
Zero-point correction=   0.594718 (a.u.)
Thermal correction to Energy=   0.642635
Thermal correction to Enthalpy=   0.643579
Thermal correction to Gibbs Free Energy=   0.512714
Sum of electronic and zero-point Energies=   -1797.815432
Sum of electronic and thermal Energies=   -1797.767516
Sum of electronic and thermal Enthalpies=   -1797.766572
Sum of electronic and thermal Free Energies=   -1797.897436

(3-5) The neutral tetrahedral intermediate Int2’(es)

zuka07.for.chk Stoichiometry C9H43O19(1-)

Standard orientation:

Center Number	Atomic Number	Atomic Type	Coordinates (Angstroms) X	Y	Z
1	6	0	-2.443018	-1.094886	-0.243629
2	6	0	-1.077415	-1.053117	0.453195
3	8	0	-0.374982	0.057453	-0.027965
4	8	0	-0.294947	-2.195187	0.122159
5	6	0	-0.788351	-3.474836	0.571680
6	6	0	-2.502676	-1.020639	-1.642185
7	6	0	-3.730119	-1.077252	-2.299410
8	6	0	-3.628291	-1.243256	0.481205
9	6	0	0.219723	-4.535372	0.163350
10	1	0	-0.910111	-3.447450	1.658583
11	1	0	-1.768642	-3.661814	0.114503
12	8	0	-1.175880	-1.032465	1.851313
13	1	0	-1.563196	-0.170638	2.165214
14	1	0	2.088190	0.934990	2.689447
15	8	0	1.924718	0.886810	3.658185
16	1	0	1.673226	-0.055781	3.783440
17	1	0	0.567238	2.004791	3.846448
18	8	0	-0.218085	2.611911	3.805177
19	1	0	-0.000717	3.282082	3.104701
20	1	0	-1.433279	1.822811	3.220924
21	8	0	-2.216884	1.319495	2.781246
22	1	0	-2.904359	1.248449	3.459913
23	1	0	-1.303083	1.752489	0.054376
24	8	0	-1.866034	2.535807	0.229681
25	1	0	-2.338759	2.276565	1.046745
26	1	0	-0.693388	3.803740	1.117817
27	8	0	0.012013	4.259796	1.622315
28	1	0	0.762828	4.242742	0.981708
29	1	0	-1.435197	3.662797	-1.387160
30	8	0	-0.874720	4.114505	-2.046001
31	1	0	-0.833619	3.524913	-2.831650
32	1	0	0.540583	0.122936	0.410667
33	8	0	2.094153	0.427400	0.879975
34	1	0	2.555845	-0.437213	0.975757
35	1	0	2.551339	0.914862	0.113337
36	8	0	3.047408	1.481682	-1.318678
37	1	0	3.590021	2.275409	-1.441314
38	1	0	1.922479	3.096545	-0.831240
39	8	0	1.585802	4.001423	-0.664399
40	1	0	0.794391	4.105553	-1.244504

41	1	0	0.363452	-1.631537	2.856697
42	8	0	1.167324	-1.853199	3.368910
43	1	0	1.832207	-2.097720	2.692766
44	1	0	1.828232	1.007480	-2.229356
45	8	0	0.979224	0.574039	-2.601012
46	1	0	0.405316	0.494441	-1.813556
47	1	0	0.132583	1.656696	-3.668588
48	8	0	-0.428422	2.310570	-4.166534
49	1	0	-1.129596	1.792259	-4.586250
50	6	0	-4.859199	-1.297287	-0.178448
51	6	0	-4.913585	-1.211683	-1.568752
52	1	0	-1.585616	-0.927932	-2.215206
53	1	0	-3.762501	-1.017839	-3.384188
54	1	0	-3.585400	-1.319346	1.562426
55	1	0	-5.774029	-1.407396	0.398338
56	1	0	-5.870792	-1.252024	-2.082454
57	1	0	0.334516	-4.580280	-0.924572
58	1	0	1.198873	-4.329585	0.606885
59	1	0	-0.119934	-5.516752	0.514405
60	1	0	1.252712	-1.293925	-2.630020
61	8	0	1.202038	-2.244142	-2.374988
62	1	0	0.665204	-2.233473	-1.557205
63	1	0	4.151669	0.346578	-1.692363
64	8	0	4.843114	-0.355436	-1.951272
65	1	0	4.937175	-0.277504	-2.911882
66	1	0	4.112443	-2.017726	1.751151
67	8	0	3.234208	-2.113016	1.352534
68	1	0	3.397224	-2.431135	0.407017
69	1	0	4.190635	-1.933715	-1.490003
70	8	0	3.693743	-2.738408	-1.191266
71	1	0	2.831088	-2.670670	-1.668841

SCF Done: E(RB3LYP) = -1798.41464479 A.U. after 1 cycles
Convg = 0.2877D-08 -V/T = 2.0093
Zero-point correction= 0.598737 (a.u.)
Thermal correction to Energy= 0.647229
Thermal correction to Enthalpy= 0.648173
Thermal correction to Gibbs Free Energy= 0.516669
Sum of electronic and zero-point Energies= -1797.815908
Sum of electronic and thermal Energies= -1797.767416
Sum of electronic and thermal Enthalpies= -1797.766471
Sum of electronic and thermal Free Energies= -1797.897975

(3-6) TS2(es), MeO- dissociation along with ethanol formation
zukaphxx.chk Stoichiometry C9H43O19(1-)
Standard orientation:

Center Number	Atomic Number	Atomic Type	Coordinates (Angstroms) X	Y	Z
1	6	0	-3.277696	-0.456962	-0.444548
2	6	0	-1.848302	-0.009070	-0.660236
3	8	0	-1.387972	0.974205	0.000929
4	8	0	-1.486841	-0.143223	-1.981988
5	1	0	-0.754402	0.480849	-2.192822
6	6	0	-4.008673	0.092464	0.613114
7	6	0	-5.335165	-0.287535	0.830182
8	6	0	-3.895718	-1.386162	-1.292627
9	8	0	-0.944167	-1.494976	-0.022935
10	6	0	-1.235077	-1.921964	1.298982
11	1	0	-1.881845	-1.182393	1.795255
12	6	0	-1.905215	-3.295308	1.336169
13	1	0	-0.301261	-1.957481	1.883135
14	1	0	0.562466	-0.889020	0.011992
15	8	0	1.563775	-0.733516	0.063702
16	1	0	1.818213	0.049217	-0.480187
17	1	0	2.110630	-2.109538	-0.800231
18	8	0	2.411976	-2.935418	-1.268746
19	1	0	1.571741	-3.233119	-1.697125
20	1	0	3.874878	-1.826116	-1.950778
21	8	0	4.488666	-1.061373	-1.979431
22	1	0	4.909436	-1.039451	-1.089883
23	1	0	3.194218	-2.402841	1.945885
24	8	0	3.145209	-1.427665	2.099746
25	1	0	2.527333	-1.110310	1.382316
26	1	0	2.054275	-0.825147	3.393721
27	8	0	1.376263	-0.374730	3.956937
28	1	0	1.878058	0.172097	4.578984
29	1	0	3.417345	0.378658	-1.912391
30	8	0	2.742970	1.066350	-1.673193
31	1	0	2.081139	1.104272	-2.399718
32	1	0	-0.586647	1.081181	1.617636
33	8	0	-0.156936	1.457469	2.425237
34	1	0	0.298786	0.728274	2.904816
35	1	0	0.916212	2.749155	1.848870
36	8	0	1.217557	3.642171	1.536486
37	1	0	0.739591	4.248458	2.132822
38	1	0	-1.198731	3.105038	2.922617
39	8	0	-1.427675	4.050077	2.810305
40	1	0	-1.696582	4.106884	1.872728
41	1	0	-1.344826	2.752728	-0.224953
42	8	0	-1.113869	3.712331	-0.165725

43	1	0	-0.247516	3.708440	0.297319
44	1	0	-0.299646	4.043655	-1.804467
45	8	0	0.379674	4.024431	-2.520625
46	1	0	1.239200	4.089830	-2.030638
47	1	0	4.679456	-0.968643	1.274360
48	8	0	5.464098	-0.793102	0.694886
49	1	0	5.668074	0.146563	0.814193
50	1	0	-0.584199	-2.622944	-1.275531
51	8	0	-0.256189	-3.148906	-2.061928
52	1	0	-0.451527	-2.556211	-2.804143
53	1	0	2.990066	-3.789738	0.176305
54	8	0	3.366068	-4.028144	1.065103
55	1	0	4.301268	-4.220406	0.900862
56	1	0	0.312211	1.384131	-4.170561
57	8	0	0.477949	1.478332	-3.220982
58	1	0	0.390251	2.465698	-3.012666
59	1	0	2.871819	2.853758	-1.141019
60	8	0	2.675882	3.796967	-0.945535
61	1	0	2.315408	3.803385	-0.035005
62	6	0	-5.220692	-1.761675	-1.078647
63	6	0	-5.944635	-1.215573	-0.014152
64	1	0	-3.529179	0.828341	1.250552
65	1	0	-5.891806	0.147444	1.656793
66	1	0	-3.330976	-1.810104	-2.115645
67	1	0	-5.690449	-2.482996	-1.743314
68	1	0	-6.978192	-1.510722	0.151940
69	1	0	-2.097479	-3.601033	2.373192
70	1	0	-2.857712	-3.276769	0.796687
71	1	0	-1.263548	-4.052613	0.870738

SCF Done: E(RB+HF-LYP) = -1798.40938611   A.U. after   1 cycles
Convg =   0.2706D-08   -V/T = 2.0093
S**2   =   0.0000
Zero-point correction=   0.597771 (a.u.)
Thermal correction to Energy=   0.646983
Thermal correction to Enthalpy=   0.647927
Thermal correction to Gibbs Free Energy=   0.512972
Sum of electronic and zero-point Energies=   -1797.811615
Sum of electronic and thermal Energies=   -1797.762403
Sum of electronic and thermal Enthalpies=   -1797.761459
Sum of electronic and thermal Free Energies=   -1797.896414

(3-7) Ph-COOH + Et-OH + OH-(H2O)15, Int2(es)
zukaphxx.for.chk   Stoichiometry C9H43O19(1-)
Standard orientation:

Center Number	Atomic Number	Atomic Type	Coordinates (Angstroms) X	Y	Z
1	6	0	-4.193976	0.341137	-0.306271
2	6	0	-3.073311	1.316272	-0.427954
3	8	0	-2.367550	1.660314	0.519539
4	8	0	-2.913768	1.789396	-1.663375
5	1	0	-2.087408	2.349659	-1.754490
6	6	0	-4.400550	-0.316598	0.915160
7	6	0	-5.442877	-1.233326	1.041538
8	6	0	-5.037490	0.070674	-1.394749
9	8	0	-0.355133	-2.736659	-0.195011
10	6	0	-0.786356	-3.295921	1.046540
11	1	0	-1.027749	-2.486302	1.747335
12	6	0	-2.012649	-4.163785	0.806592
13	1	0	0.021667	-3.897180	1.491356
14	1	0	0.384132	-2.067892	-0.020314
15	8	0	1.670034	-1.049276	0.159293
16	1	0	1.421906	-0.272406	-0.379055
17	1	0	2.387987	-1.929564	-1.069778
18	8	0	2.859456	-2.393765	-1.835464
19	1	0	2.212385	-3.067452	-2.149344
20	1	0	3.287948	-0.805725	-2.751490
21	8	0	3.443353	0.131511	-3.009112
22	1	0	4.070772	0.488171	-2.332972
23	1	0	4.661120	-1.624809	0.547541
24	8	0	4.205786	-0.856350	0.965702
25	1	0	3.244123	-0.914400	0.644553
26	1	0	3.992912	-0.985124	2.675642
27	8	0	3.674409	-0.969244	3.621144
28	1	0	4.210040	-0.292597	4.059560
29	1	0	2.008931	0.881112	-2.363909
30	8	0	1.341290	1.239841	-1.716759
31	1	0	0.645582	1.729680	-2.196823
32	1	0	1.312665	-0.519892	1.647904
33	8	0	1.169882	-0.135529	2.584532
34	1	0	1.918069	-0.490860	3.116732
35	1	0	1.426486	1.490519	2.468576
36	8	0	1.575440	2.487937	2.392446
37	1	0	1.955144	2.765645	3.239004
38	1	0	-0.683609	-0.087319	2.981430
39	8	0	-1.646155	0.101389	3.003684
40	1	0	-1.798969	0.663503	2.223831
41	1	0	-1.342745	3.180209	1.191539
42	8	0	-0.739964	3.870313	1.531790
43	1	0	0.001537	3.373345	1.944740

44	1	0	0.048753	4.593871	0.119716
45	8	0	0.542171	4.770171	-0.724381
46	1	0	1.419163	4.339843	-0.576553
47	1	0	4.732834	0.543463	-0.136967
48	8	0	4.897046	1.223386	-0.832146
49	1	0	4.319835	1.976186	-0.581746
50	1	0	0.211973	-3.674764	-1.512276
51	8	0	0.642931	-4.064269	-2.325603
52	1	0	0.115597	-3.731893	-3.066903
53	1	0	4.390763	-2.879496	-1.162742
54	8	0	5.248233	-2.915453	-0.658388
55	1	0	5.899645	-2.499937	-1.242951
56	1	0	-0.843289	3.597790	-3.210556
57	8	0	-0.702742	3.146102	-2.365120
58	1	0	-0.232912	3.814705	-1.756225
59	1	0	2.201448	2.355504	-0.708156
60	8	0	2.699354	3.030587	-0.174541
61	1	0	2.452978	2.841712	0.757515
62	6	0	-6.082383	-0.840299	-1.258633
63	6	0	-6.286355	-1.493750	-0.040722
64	1	0	-3.735679	-0.129278	1.751059
65	1	0	-5.591293	-1.746676	1.987341
66	1	0	-4.865744	0.574816	-2.338959
67	1	0	-6.734249	-1.044843	-2.103960
68	1	0	-7.098603	-2.209251	0.062506
69	1	0	-2.349600	-4.616812	1.747128
70	1	0	-2.833651	-3.564577	0.397947
71	1	0	-1.787806	-4.970313	0.098374

SCF Done: E(RB+HF-LYP) = -1798.44248409   A.U. after   1 cycles
Convg =   0.3169D-08   -V/T = 2.0093
S**2   =   0.0000
Zero-point correction=   0.598206 (a.u.)
Thermal correction to Energy=   0.648012
Thermal correction to Enthalpy=   0.648956
Thermal correction to Gibbs Free Energy=   0.509867
Sum of electronic and zero-point Energies=   -1797.844278
Sum of electronic and thermal Energies=   -1797.794472
Sum of electronic and thermal Enthalpies=   -1797.793528
Sum of electronic and thermal Free Energies=   -1797.932617

(3-8) TS3(es) toward the product, PhCOO-, EtOH and (H2O)16,
carboxylate formation TS, TS3(es),
zukaphqd.g09.chk   Stoichiometry C9H43O19(1-)
Standard orientation:

Center Number	Atomic Number	Atomic Type	Coordinates (Angstroms) X	Y	Z
1	6	0	2.036322	2.500396	0.716031
2	6	0	1.909812	1.150472	1.353015
3	8	0	2.914070	0.447730	1.555548
4	8	0	0.318486	-2.944046	-1.562960
5	6	0	-0.110992	-4.151317	-2.210893
6	6	0	3.198964	3.253240	0.944045
7	6	0	3.353802	4.501892	0.343613
8	6	0	1.036135	3.009249	-0.122835
9	1	0	-0.721390	-3.896559	-3.087719
10	1	0	-0.735378	-4.732893	-1.519061
11	6	0	1.112609	-4.950554	-2.627568
12	8	0	0.688578	0.806480	1.666504
13	1	0	-2.260745	-0.022289	1.454031
14	1	0	0.527563	-0.197389	2.081489
15	8	0	0.043161	-1.413979	2.541757
16	1	0	0.423671	-1.659501	3.419380
17	1	0	1.057858	-1.668522	-2.753803
18	8	0	1.223272	-0.956092	-3.407913
19	1	0	2.145784	-0.657610	-3.257490
20	8	0	-2.054632	0.699559	0.725116
21	1	0	-1.815200	-0.703556	-0.565800
22	1	0	-1.204383	1.074424	1.020041
23	8	0	-2.430225	-1.202052	2.380535
24	1	0	-1.040192	-1.385072	2.569535
25	1	0	-2.861512	-0.949293	3.210338
26	1	0	0.732609	-2.784371	1.521053
27	8	0	1.334862	-3.405062	1.049727
28	1	0	1.116148	-3.278938	0.100279
29	8	0	-1.902783	-1.421712	-1.233307
30	1	0	-0.492596	-2.406098	-1.341850
31	1	0	-1.843496	-0.945461	-2.110788
32	1	0	3.296770	-1.329875	2.230344
33	8	0	3.372939	-2.259713	2.524447
34	1	0	2.807964	-2.756651	1.881462
35	8	0	-2.158405	2.214390	-1.788640
36	1	0	-2.186583	1.718756	-0.945485
37	1	0	-3.108880	2.360930	-1.999252
38	8	0	-4.985421	2.262719	-1.940182
39	1	0	-5.077312	1.631593	-1.181975
40	1	0	-5.482973	3.049967	-1.674478
41	1	0	4.226611	0.536469	0.222657
42	8	0	4.811194	0.733534	-0.544868
43	1	0	4.719752	1.690848	-0.669896

44	1	0	4.256618	-0.019601	-2.071558
45	8	0	3.980121	-0.366981	-2.954242
46	1	0	4.479339	-1.187297	-3.077858
47	1	0	-3.623462	-1.823936	1.217544
48	8	0	-4.226169	-1.901614	0.427352
49	1	0	-3.602728	-1.971836	-0.324860
50	1	0	-3.992744	1.020436	0.628488
51	8	0	-4.890001	0.735260	0.356843
52	1	0	-4.792561	-0.260778	0.316529
53	8	0	-1.400483	0.049349	-3.454810
54	1	0	-0.428894	-0.095184	-3.523973
55	1	0	-1.544350	0.923306	-3.026704
56	8	0	1.661088	-2.251286	4.642235
57	1	0	1.505813	-3.170858	4.903652
58	1	0	2.389517	-2.289075	3.960020
59	8	0	-6.977170	1.580539	2.059644
60	1	0	-6.229476	1.301961	1.481970
61	1	0	-7.336579	0.751392	2.407048
62	6	0	1.203692	4.250024	-0.737908
63	6	0	2.358162	4.998642	-0.503059
64	1	0	3.962992	2.859267	1.608241
65	1	0	4.248349	5.088032	0.537806
66	1	0	0.140447	2.433601	-0.324746
67	1	0	0.427907	4.620485	-1.401192
68	1	0	2.484082	5.967915	-0.978714
69	1	0	1.729616	-4.375932	-3.327050
70	1	0	1.723879	-5.208388	-1.755604
71	1	0	0.807267	-5.880872	-3.120978

SCF Done: E(RB3LYP) = -1798.42252801 A.U. after 15 cycles
Convg = 0.2447D-08 -V/T = 2.0093
Zero-point correction= 0.590177 (a.u.)
Thermal correction to Energy= 0.641587
Thermal correction to Enthalpy= 0.642531
Thermal correction to Gibbs Free Energy= 0.497017
Sum of electronic and zero-point Energies= -1797.832351
Sum of electronic and thermal Energies= -1797.780941
Sum of electronic and thermal Enthalpies= -1797.779997
Sum of electronic and thermal Free Energies= -1797.925511

(3-9) Product, Ph-COO-, EtOH and OH-(H2O)16
zukaphqd.g09for.chk Stoichiometry C9H43O19(1-)
Standard orientation:

Center Number	Atomic Number	Atomic Type	Coordinates (Angstroms) X	Y	Z

1	6	0	2.219578	2.139236	0.842410
2	6	0	1.923938	0.731695	1.320712
3	8	0	2.918525	-0.024143	1.524212
4	8	0	-0.185925	-2.827564	-1.798361
5	6	0	-0.627622	-3.958382	-2.564240
6	6	0	3.368116	2.801805	1.301942
7	6	0	3.658344	4.097017	0.870804
8	6	0	1.376885	2.792605	-0.064693
9	1	0	-1.058588	-3.610937	-3.513216
10	1	0	-1.411547	-4.491939	-2.008992
11	6	0	0.559777	-4.871739	-2.819788
12	8	0	0.709770	0.407138	1.494778
13	1	0	-2.188560	0.162170	1.149848
14	1	0	0.053178	-1.008404	2.352242
15	8	0	-0.478975	-1.760878	2.723587
16	1	0	0.048229	-2.114184	3.481699
17	1	0	0.812747	-1.570209	-2.854656
18	8	0	1.080835	-0.868694	-3.484645
19	1	0	2.010309	-0.639534	-3.259418
20	8	0	-1.761933	0.809997	0.528895
21	1	0	-1.907845	-0.377443	-0.904654
22	1	0	-0.819097	0.855268	0.850324
23	8	0	-3.019880	-1.014296	2.174836
24	1	0	-2.191889	-1.375066	2.586868
25	1	0	-3.511393	-0.476141	2.832208
26	1	0	0.036492	-3.046286	1.431097
27	8	0	0.559913	-3.643216	0.850841
28	1	0	0.376440	-3.344465	-0.066150
29	8	0	-2.207248	-0.994241	-1.613231
30	1	0	-0.956380	-2.212033	-1.670144
31	1	0	-2.013562	-0.493549	-2.460441
32	1	0	2.889542	-1.785661	1.996860
33	8	0	2.840777	-2.744130	2.229916
34	1	0	2.176308	-3.119339	1.604682
35	8	0	-1.763474	2.641465	-1.871371
36	1	0	-1.772815	2.103032	-1.057856
37	1	0	-2.670333	3.017230	-1.921219
38	8	0	-4.460081	3.524067	-1.508504
39	1	0	-4.636050	2.886434	-0.773889
40	1	0	-4.523428	4.404544	-1.110315
41	1	0	4.189568	0.109361	0.298625
42	8	0	4.813458	0.304885	-0.450137
43	1	0	4.860064	1.273260	-0.463463
44	1	0	4.138867	-0.206654	-1.997860
45	8	0	3.824867	-0.478742	-2.898705

46	1	0	4.278548	-1.314039	-3.082664
47	1	0	-4.182598	-1.280129	0.627216
48	8	0	-4.630780	-0.959268	-0.183552
49	1	0	-3.944749	-1.065859	-0.883124
50	1	0	-3.490773	1.669166	0.622887
51	8	0	-4.470788	1.664031	0.558759
52	1	0	-4.654144	0.754274	0.185961
53	8	0	-1.349041	0.491579	-3.690083
54	1	0	-0.408952	0.189933	-3.700295
55	1	0	-1.375254	1.355328	-3.222302
56	8	0	1.366157	-2.869816	4.479592
57	1	0	1.223322	-3.812856	4.647460
58	1	0	2.021849	-2.835555	3.720585
59	8	0	-4.834060	0.862922	3.230160
60	1	0	-4.825465	1.269647	2.329815
61	1	0	-5.724283	0.498637	3.342652
62	6	0	1.678089	4.078641	-0.514149
63	6	0	2.816014	4.736362	-0.043056
64	1	0	4.019357	2.294013	2.007588
65	1	0	4.541929	4.607041	1.247672
66	1	0	0.494086	2.290927	-0.442657
67	1	0	1.019382	4.557697	-1.233086
68	1	0	3.047650	5.741529	-0.387324
69	1	0	1.336266	-4.345479	-3.385478
70	1	0	0.992363	-5.217080	-1.874665
71	1	0	0.245581	-5.748867	-3.397969

SCF Done: E(RB+HF-LYP) = -1798.45216901   A.U. after   1 cycles

Convg =   0.1812D-08   -V/T = 2.0093

S**2   =   0.0000

Zero-point correction=   0.598603 (a.u.)

Thermal correction to Energy=   0.649832

Thermal correction to Enthalpy=   0.650776

Thermal correction to Gibbs Free Energy=   0.506339

Sum of electronic and zero-point Energies=   -1797.853566

Sum of electronic and thermal Energies=   -1797.802337

Sum of electronic and thermal Enthalpies=   -1797.801393

Sum of electronic and thermal Free Energies=   -1797.945830

==

[4] Geometries of the n=16 amide hydrolysis in Figure 4

(4-1) N-ethyl benzamide(C6H5-C(=O)-NH-C2H5) + HO-(H2O)16,
Reactant-like complex (precursor)
tuka02aa.for.chk   Stoichiometry C9H44NO18(1-)
Standard orientation:

Center Number	Atomic Number	Atomic Type	Coordinates (Angstroms) X	Y	Z
1	6	0	-0.861514	2.709843	-0.501915
2	6	0	-0.690971	1.733148	0.623989
3	8	0	-1.420190	0.704973	0.673568
4	7	0	0.198295	2.031833	1.579504
5	6	0	0.502385	1.152724	2.711487
6	6	0	-2.117233	2.786377	-1.120274
7	6	0	-2.336311	3.684164	-2.163839
8	6	0	0.179791	3.532840	-0.955945
9	1	0	0.867083	2.785764	1.420775
10	6	0	0.927105	1.965886	3.932425
11	1	0	-0.391108	0.566469	2.930386
12	1	0	1.292101	0.450170	2.418390
13	8	0	1.800161	0.573021	-1.007530
14	1	0	1.415834	0.314744	-1.868968
15	1	0	1.820346	-0.262685	-0.444009
16	8	0	2.048665	-1.644330	0.456905
17	1	0	2.685518	-1.419381	1.154218
18	1	0	2.740073	-2.246120	-0.557112
19	8	0	3.320823	-2.682721	-1.389643
20	1	0	3.607329	-1.919098	-1.917349
21	1	0	4.675702	-3.026385	-0.452523
22	8	0	5.414589	-3.075584	0.228531
23	1	0	5.157318	-3.799741	0.818625
24	1	0	1.885758	-3.106436	-2.481622
25	8	0	1.152818	-3.050877	-3.140570
26	1	0	0.342078	-3.426739	-2.713636
27	1	0	0.735115	-1.473943	-3.304279
28	8	0	0.431496	-0.507791	-3.325079
29	1	0	0.483269	-0.224639	-4.249477
30	1	0	5.233637	-1.506476	1.020961
31	8	0	5.054676	-0.638899	1.472446
32	1	0	5.843688	-0.450428	2.001146
33	1	0	-1.765896	-0.256752	-1.013946
34	8	0	-1.976963	-0.715244	-1.854467
35	1	0	-1.221079	-0.513885	-2.451679
36	1	0	-1.625342	-2.651273	-2.056358

37	8	0	-1.355092	-3.588200	-2.154826
38	1	0	-1.640726	-3.984455	-1.303000
39	1	0	-3.710527	-1.546114	-1.314937
40	8	0	-4.306091	-2.108197	-0.783809
41	1	0	-4.814834	-1.506730	-0.194797
42	1	0	-0.703828	-1.041623	1.125764
43	8	0	-0.396350	-1.938459	1.363296
44	1	0	0.568963	-1.949541	1.047982
45	1	0	-1.444357	-2.417297	2.850279
46	8	0	-2.328893	-2.616090	3.233185
47	1	0	-2.693022	-3.228655	2.562453
48	1	0	-1.655209	-3.102923	0.673297
49	8	0	-2.397843	-3.728163	0.500124
50	1	0	-3.129147	-3.183637	0.119158
51	1	0	3.332473	1.267120	-0.539690
52	8	0	4.107862	1.629142	-0.036233
53	1	0	4.461253	0.863013	0.466471
54	1	0	3.328200	2.800314	0.916351
55	8	0	2.779249	3.491708	1.391457
56	1	0	3.215407	4.337144	1.213258
57	1	0	-3.091092	-0.994891	2.938067
58	8	0	-3.401774	-0.099038	2.648308
59	1	0	-2.727902	0.209064	2.008869
60	1	0	-4.840916	-0.315391	1.729737
61	8	0	-5.603076	-0.466255	1.103993
62	1	0	-5.944689	0.412480	0.886411
63	6	0	-0.041641	4.422326	-2.006988
64	6	0	-1.298655	4.505151	-2.609470
65	1	0	-2.912840	2.133405	-0.777830
66	1	0	-3.315344	3.736255	-2.632579
67	1	0	1.168678	3.455182	-0.517107
68	1	0	0.775669	5.044505	-2.362531
69	1	0	-1.465934	5.199529	-3.429295
70	1	0	1.807594	2.577053	3.709358
71	1	0	0.116881	2.625437	4.263823
72	1	0	1.175471	1.288048	4.756365

SCF Done: E(RB+HF-LYP) = -1778.55871751   A.U. after   1 cycles
Convg =   0.3275D-08   -V/T = 2.0093
S**2   =   0.0000
Zero-point correction=   0.606387 (a.u.)
Thermal correction to Energy=   0.657935
Thermal correction to Enthalpy=   0.658880
Thermal correction to Gibbs Free Energy=   0.516969
Sum of electronic and zero-point Energies=   -1777.952330
Sum of electronic and thermal Energies=   -1777.900782

Sum of electronic and thermal Enthalpies= -1777.899838
Sum of electronic and thermal Free Energies= -1778.041748

(4-2) N-ethyl benzamide + HO-(H2O)16 TS1(am)
tuka02aa.chk Stoichiometry C9H44NO18(1-)
Standard orientation:

Center Number	Atomic Number	Atomic Type	Coordinates (Angstroms) X	Y	Z
1	6	0	-0.470652	2.399800	-0.844394
2	6	0	-0.180315	1.366931	0.254620
3	8	0	-1.118661	0.470879	0.459457
4	7	0	0.342700	2.010664	1.397317
5	6	0	0.645579	1.245170	2.603188
6	6	0	-1.799864	2.635945	-1.211927
7	6	0	-2.113841	3.598584	-2.173143
8	6	0	0.540684	3.151935	-1.458527
9	1	0	1.076941	2.683243	1.182820
10	6	0	0.885925	2.186439	3.783055
11	1	0	-0.202787	0.591747	2.814586
12	1	0	1.528264	0.597069	2.466522
13	8	0	1.157664	0.509965	-0.411976
14	1	0	0.896889	0.227086	-1.314410
15	1	0	1.587771	-0.852260	0.345443
16	8	0	1.952299	-1.713037	0.748674
17	1	0	2.700652	-1.434461	1.313807
18	1	0	2.706877	-2.407177	-0.567744
19	8	0	3.249789	-2.785737	-1.325315
20	1	0	3.390337	-2.037032	-1.927494
21	1	0	4.767183	-3.037333	-0.478314
22	8	0	5.544084	-3.023980	0.142972
23	1	0	5.564892	-3.901335	0.552826
24	1	0	1.612379	-3.375996	-2.394466
25	8	0	0.918694	-3.204272	-3.058986
26	1	0	0.050873	-3.504101	-2.664685
27	1	0	0.661174	-1.532679	-3.126138
28	8	0	0.461399	-0.551175	-3.066459
29	1	0	0.752093	-0.153017	-3.899660
30	1	0	5.065508	-1.649753	1.162312
31	8	0	4.574746	-0.895313	1.578999
32	1	0	5.059210	-0.636911	2.376590
33	1	0	-1.776876	-0.345121	-0.967672
34	8	0	-2.108694	-0.773240	-1.803528
35	1	0	-1.432968	-0.526168	-2.465828
36	1	0	-1.815175	-2.628230	-2.039072

37	8	0	-1.576407	-3.579154	-2.121011
38	1	0	-1.869970	-3.937887	-1.248433
39	1	0	-3.831575	-1.515954	-1.181114
40	8	0	-4.464323	-2.010375	-0.624051
41	1	0	-4.860708	-1.346189	-0.014687
42	1	0	-0.847662	-1.064812	1.165701
43	8	0	-0.733012	-1.960657	1.595774
44	1	0	0.223355	-2.144736	1.488287
45	1	0	-1.656867	-1.836059	3.101483
46	8	0	-2.343048	-1.621517	3.782176
47	1	0	-3.046979	-2.270386	3.628954
48	1	0	-1.944807	-3.214478	0.852215
49	8	0	-2.601781	-3.826397	0.454936
50	1	0	-3.353363	-3.247451	0.179614
51	1	0	2.816465	1.024139	-0.349095
52	8	0	3.769296	1.284433	-0.214281
53	1	0	4.141875	0.590024	0.364135
54	1	0	3.527289	2.751943	0.736901
55	8	0	3.220396	3.527803	1.273584
56	1	0	3.423512	4.310456	0.741796
57	1	0	-2.912549	-0.008539	2.964230
58	8	0	-3.161896	0.718587	2.350330
59	1	0	-2.434337	0.745699	1.680167
60	1	0	-4.616935	0.249836	1.616685
61	8	0	-5.448266	-0.072571	1.162537
62	1	0	-5.847773	0.712152	0.760954
63	6	0	0.227862	4.115941	-2.418266
64	6	0	-1.101764	4.343406	-2.779777
65	1	0	-2.583159	2.052290	-0.741269
66	1	0	-3.152852	3.764280	-2.447800
67	1	0	1.577652	2.972046	-1.194422
68	1	0	1.026415	4.686340	-2.887611
69	1	0	-1.345600	5.092438	-3.529757
70	1	0	1.720521	2.865998	3.576164
71	1	0	-0.009187	2.785962	3.980437
72	1	0	1.125183	1.611812	4.686197

SCF Done: E(RB+HF-LYP) = -1778.52641035 A.U. after 1 cycles
Convg = 0.4358D-08 -V/T = 2.0093
S**2 = 0.0000
Zero-point correction= 0.608558 (a.u.)
Thermal correction to Energy= 0.659034
Thermal correction to Enthalpy= 0.659978
Thermal correction to Gibbs Free Energy= 0.521684
Sum of electronic and zero-point Energies= -1777.917853
Sum of electronic and thermal Energies= -1777.867376

Sum of electronic and thermal Enthalpies= -1777.866432
Sum of electronic and thermal Free Energies= -1778.004727

(4-3) Anionic tetrahedral intermediate, Ph-C(-O-)(OH)-NHEt.
and (H2O)16, Int1(am)
tuka02bz.for.chk Stoichiometry C9H44NO18(1-)
Standard orientation:

Center Number	Atomic Number	Atomic Type	Coordinates (Angstroms) X	Y	Z
1	6	0	-2.067584	1.034754	-1.070918
2	6	0	-2.457612	-0.414114	-0.686843
3	8	0	-2.751103	-0.572288	0.617198
4	7	0	-1.339821	-1.302903	-1.096324
5	6	0	-1.564909	-2.744696	-0.866782
6	6	0	-1.958955	2.030089	-0.094613
7	6	0	-1.587899	3.333692	-0.443703
8	6	0	-1.313346	3.657355	-1.774834
9	1	0	-1.158577	-1.155697	-2.089346
10	1	0	-1.673067	-2.894227	0.210747
11	1	0	-2.496669	-3.084170	-1.339183
12	6	0	-0.386642	-3.560354	-1.395960
13	8	0	-3.599778	-0.782632	-1.536658
14	1	0	-4.335859	-0.183612	-1.296683
15	1	0	-5.378808	-2.690503	0.302076
16	8	0	-5.720654	-2.549036	-0.617186
17	1	0	-4.911031	-2.289241	-1.103936
18	1	0	0.107994	-0.783608	-0.169393
19	8	0	0.835107	-0.456274	0.447240
20	1	0	0.371427	-0.359323	1.313565
21	1	0	2.526871	-1.109194	0.402407
22	8	0	3.497338	-1.101165	0.233204
23	1	0	3.952325	-1.864076	0.668396
24	1	0	-6.279761	-0.891325	-0.528003
25	8	0	-6.273330	0.106477	-0.530482
26	1	0	-7.025452	0.389068	-1.070454
27	1	0	-4.077507	0.236117	1.399659
28	8	0	-4.837317	0.505058	1.982080
29	1	0	-5.560743	0.655204	1.345331
30	1	0	4.178602	-0.957740	-1.530744
31	8	0	4.768583	-0.704682	-2.272788
32	1	0	4.684572	0.275613	-2.284350
33	1	0	4.310649	0.350994	0.782877
34	8	0	4.874743	1.136011	1.013423
35	1	0	4.292906	1.732146	1.540572

36	1	0	-3.673658	-1.917405	1.315724
37	8	0	-4.372549	-2.428948	1.802795
38	1	0	-4.831264	-1.710639	2.278351
39	1	0	6.335458	0.311650	1.476782
40	8	0	7.073155	-0.345797	1.586263
41	1	0	7.803916	0.129573	2.006959
42	1	0	6.287328	-1.409699	-1.522286
43	8	0	6.943310	-1.857370	-0.932353
44	1	0	7.203696	-1.183599	-0.274887
45	1	0	-1.087765	-1.005513	3.122527
46	8	0	-1.003326	-0.241127	2.532190
47	1	0	-1.675592	-0.395624	1.783945
48	1	0	-2.136801	1.222803	3.368597
49	8	0	-2.813242	1.884631	3.610345
50	1	0	-3.634275	1.560642	3.188606
51	1	0	4.674528	1.896816	-0.759697
52	8	0	4.278766	2.033619	-1.647049
53	1	0	3.321465	2.081946	-1.451292
54	1	0	0.815768	2.517386	-0.649988
55	8	0	1.618305	2.050728	-0.358979
56	1	0	1.313988	1.132913	-0.118258
57	6	0	-1.819063	1.377674	-2.409636
58	6	0	-1.435777	2.672038	-2.760064
59	1	0	-2.169131	1.795320	0.942208
60	1	0	-1.517779	4.092297	0.331583
61	1	0	-1.020490	4.668824	-2.045106
62	1	0	-1.944369	0.629505	-3.188135
63	1	0	-1.238319	2.914074	-3.801278
64	1	0	-0.262775	-3.428882	-2.478911
65	1	0	0.551677	-3.267257	-0.912802
66	1	0	-0.549644	-4.627742	-1.209502
67	1	0	2.344756	2.656711	1.166887
68	8	0	2.873671	2.809113	1.989543
69	1	0	2.291095	2.561152	2.722928
70	1	0	5.887994	-2.246807	1.778406
71	8	0	5.406533	-2.838816	1.172905
72	1	0	5.926085	-2.733198	0.335964

SCF Done: E(RB+HF-LYP) = -1778.53261788   A.U. after   1 cycles

Convg =   0.3824D-08   -V/T = 2.0093

S**2   =   0.0000

Zero-point correction=   0.609918 (a.u.)

Thermal correction to Energy=   0.660861

Thermal correction to Enthalpy=   0.661805

Thermal correction to Gibbs Free Energy=   0.520198

Sum of electronic and zero-point Energies=   -1777.922699

Sum of electronic and thermal Energies= -1777.871757
Sum of electronic and thermal Enthalpies= -1777.870813
Sum of electronic and thermal Free Energies= -1778.012420

(4-4) TS2(am) toward the zwitterion
tuka02bz.chk Stoichiometry C9H44NO18(1-)
Standard orientation:

Center Number	Atomic Number	Atomic Type	Coordinates (Angstroms) X	Y	Z
1	6	0	-1.426006	0.980577	-0.999213
2	6	0	-1.786729	-0.421078	-0.455536
3	8	0	-2.578588	-0.402326	0.624639
4	7	0	-0.478914	-1.140013	-0.117061
5	6	0	-0.683466	-2.536538	0.362438
6	6	0	-1.793698	2.134399	-0.299002
7	6	0	-1.486990	3.401563	-0.806915
8	6	0	-0.800916	3.527668	-2.017650
9	1	0	0.146908	-1.152380	-0.931889
10	1	0	-1.229858	-2.469040	1.305768
11	1	0	-1.318884	-3.057636	-0.358032
12	6	0	0.638982	-3.273683	0.554122
13	8	0	-2.330426	-1.200880	-1.535684
14	1	0	-3.236708	-0.863874	-1.702930
15	1	0	-5.100238	-2.891886	-0.082925
16	8	0	-5.417161	-3.143475	-0.999782
17	1	0	-4.687202	-3.655178	-1.380562
18	1	0	0.104738	-0.493688	0.726444
19	8	0	0.785877	0.223401	1.602588
20	1	0	0.183983	0.404241	2.348025
21	1	0	2.305833	-0.405076	1.595610
22	8	0	3.196585	-0.637142	1.184077
23	1	0	3.591935	-1.460900	1.547352
24	1	0	-5.366391	-1.536602	-1.685492
25	8	0	-5.151141	-0.597555	-1.936673
26	1	0	-5.473203	-0.469080	-2.840633
27	1	0	-4.132038	0.364554	0.633142
28	8	0	-5.085576	0.608137	0.784389
29	1	0	-5.468738	0.543553	-0.108618
30	1	0	2.633217	-1.020960	-0.508125
31	8	0	2.440339	-1.152233	-1.467662
32	1	0	2.620638	-0.252755	-1.835051
33	1	0	4.336556	0.538531	0.680169
34	8	0	5.009973	1.148281	0.273406
35	1	0	4.880657	2.000778	0.750307

36	1	0	-3.658976	-1.720010	1.057869
37	8	0	-4.488674	-2.189139	1.350947
38	1	0	-5.084270	-1.427532	1.496466
39	1	0	6.254229	-0.054466	0.013515
40	8	0	6.733955	-0.909165	-0.145561
41	1	0	7.609123	-0.669561	-0.482678
42	1	0	3.978844	-2.230211	-1.588311
43	8	0	4.800639	-2.731836	-1.372881
44	1	0	5.492576	-2.053099	-1.241742
45	1	0	-2.145580	-0.140940	3.660184
46	8	0	-1.856804	0.530201	3.023786
47	1	0	-2.086228	0.156068	2.120884
48	1	0	-3.310854	1.904746	3.021787
49	8	0	-4.097240	2.466401	2.867742
50	1	0	-4.612551	1.983963	2.192251
51	1	0	3.929226	1.470476	-1.318630
52	8	0	3.104753	1.538949	-1.845956
53	1	0	2.466792	1.898178	-1.189394
54	1	0	0.805915	2.919446	0.168080
55	8	0	1.600155	2.429492	0.438460
56	1	0	1.252221	1.590785	0.911587
57	6	0	-0.751043	1.117312	-2.220945
58	6	0	-0.432870	2.379110	-2.724060
59	1	0	-2.325836	2.048201	0.641008
60	1	0	-1.793901	4.285767	-0.253776
61	1	0	-0.558021	4.512018	-2.410395
62	1	0	-0.481563	0.230058	-2.786635
63	1	0	0.104183	2.464446	-3.664766
64	1	0	1.202412	-3.341508	-0.382170
65	1	0	1.271665	-2.778389	1.296602
66	1	0	0.438730	-4.292206	0.905971
67	1	0	3.026573	3.148059	1.171342
68	8	0	3.914585	3.386642	1.547680
69	1	0	3.812637	3.296255	2.506525
70	1	0	5.704698	-2.335997	1.422046
71	8	0	4.876556	-2.846946	1.428272
72	1	0	4.743094	-3.029493	0.465511

SCF Done: E(RB+HF-LYP) = -1778.52293385   A.U. after   1 cycles
Convg =   0.7811D-09   -V/T = 2.0093
S**2   =   0.0000

Zero-point correction=   0.605542 (a.u.)
Thermal correction to Energy=   0.655730
Thermal correction to Enthalpy=   0.656674
Thermal correction to Gibbs Free Energy=   0.517388
Sum of electronic and zero-point Energies=   -1777.917392

Sum of electronic and thermal Energies= -1777.867204
Sum of electronic and thermal Enthalpies= -1777.866260
Sum of electronic and thermal Free Energies= -1778.005546

(4-5) Zwitterion(Mulliken CT complex), Ph-C(-O-)(OH)NH2(+)Et, and OH-(H2O)15. Int2(am)
tukats3a.rev.chk Stoichiometry C9H44NO18(1-)
Standard orientation:

Center Number	Atomic Number	Atomic Type	Coordinates (Angstroms) X	Y	Z
1	6	0	-2.439399	1.804122	-0.099189
2	6	0	-1.805413	0.408481	-0.212268
3	8	0	-2.610018	-0.607732	-0.040179
4	7	0	-0.630464	0.343444	0.957058
5	6	0	-1.098495	0.232628	2.371488
6	6	0	-3.644497	1.958688	0.595504
7	6	0	-4.234641	3.217784	0.725993
8	6	0	-3.630560	4.339123	0.156600
9	1	0	-0.072267	-0.501527	0.734281
10	6	0	-1.047097	1.555605	3.130421
11	1	0	-2.112525	-0.173727	2.334463
12	1	0	-0.464471	-0.511673	2.862654
13	8	0	-1.006667	0.357775	-1.382329
14	1	0	-1.031312	-0.562879	-1.744583
15	1	0	-0.983017	-3.888192	-0.249569
16	8	0	-0.150609	-3.838334	-0.782471
17	1	0	0.437292	-3.257086	-0.243265
18	1	0	0.019543	1.143325	0.841618
19	8	0	1.394248	2.432852	0.601870
20	1	0	1.347030	3.349900	0.908154
21	1	0	2.361464	2.077250	0.814268
22	8	0	3.698809	1.471961	0.917096
23	1	0	3.957164	1.217365	1.815208
24	1	0	-0.629643	-2.839024	-2.015457
25	8	0	-0.965143	-2.143753	-2.666242
26	1	0	-0.343526	-2.142034	-3.409337
27	1	0	-3.493702	-1.155630	-1.601154
28	8	0	-3.803842	-1.702491	-2.360126
29	1	0	-2.960709	-2.029990	-2.730495
30	1	0	3.437160	0.068180	0.046822
31	8	0	3.341487	-0.741925	-0.546401
32	1	0	3.469619	-0.379954	-1.458860
33	1	0	4.868659	1.772490	0.020420
34	8	0	5.632218	1.843832	-0.687297

35	1	0	5.981229	2.745193	-0.629437
36	1	0	-2.370908	-2.404604	0.390872
37	8	0	-2.465558	-3.354095	0.637227
38	1	0	-3.299123	-3.628403	0.184618
39	1	0	6.706300	0.558896	-0.047308
40	8	0	7.236388	-0.196509	0.314579
41	1	0	7.598542	-0.640839	-0.466479
42	1	0	4.595053	-1.755855	0.207658
43	8	0	5.202075	-2.170996	0.870406
44	1	0	5.946586	-1.533775	0.923446
45	1	0	1.926312	-1.499256	0.084800
46	8	0	1.236974	-1.905278	0.676218
47	1	0	1.769251	-2.214986	1.447224
48	1	0	3.169771	-3.684279	2.687454
49	8	0	3.155179	-2.719537	2.602125
50	1	0	3.978807	-2.495571	2.089696
51	1	0	-4.270579	-2.144786	1.977319
52	8	0	-4.768049	-1.448459	1.517138
53	1	0	-4.074084	-1.011088	0.967491
54	1	0	-5.299009	-2.826525	0.046972
55	8	0	-5.004541	-3.503190	-0.595078
56	1	0	-4.696887	-2.972163	-1.369433
57	1	0	4.472357	1.337700	-2.231239
58	8	0	3.803508	0.847094	-2.752654
59	1	0	2.957713	1.346755	-2.656605
60	1	0	0.593392	1.406064	-2.162363
61	8	0	1.319685	2.059209	-2.171783
62	1	0	1.415886	2.280865	-1.215600
63	6	0	-1.843370	2.932940	-0.678640
64	6	0	-2.435384	4.190426	-0.549770
65	1	0	-4.128990	1.090792	1.027902
66	1	0	-5.172899	3.315855	1.266443
67	1	0	-4.091689	5.319233	0.253703
68	1	0	-0.924492	2.830670	-1.243650
69	1	0	-1.963338	5.053211	-1.013537
70	1	0	-0.021877	1.936282	3.188837
71	1	0	-1.674326	2.316054	2.657079
72	1	0	-1.412527	1.401489	4.151947

SCF Done: E(RB3LYP) = -1778.53687506 A.U. after 1 cycles

Convg = 0.1842D-08 -V/T = 2.0093

Zero-point correction= 0.610433 (a.u.)

Thermal correction to Energy= 0.660107

Thermal correction to Enthalpy= 0.661051

Thermal correction to Gibbs Free Energy= 0.525179

Sum of electronic and zero-point Energies= -1777.926443

Sum of electronic and thermal Energies= -1777.876768
Sum of electronic and thermal Enthalpies= -1777.875824
Sum of electronic and thermal Free Energies= -1778.011696

(4-6) TS3(am) separation TS to Ph-COOH, Et-NH2 and OH-
tukats3a.chk Stoichiometry C9H44NO18(1-)
Standard orientation:

Center Number	Atomic Number	Atomic Type	Coordinates (Angstroms) X	Y	Z
1	6	0	-2.513671	1.819091	-0.178684
2	6	0	-1.966925	0.418312	-0.360133
3	8	0	-2.720371	-0.582303	-0.123813
4	7	0	-0.629241	0.383448	1.084708
5	6	0	-1.153917	0.268281	2.461286
6	6	0	-3.683180	2.001384	0.570112
7	6	0	-4.215926	3.278310	0.752941
8	6	0	-3.590474	4.387951	0.182828
9	1	0	-0.074423	-0.453199	0.863533
10	6	0	-1.149938	1.598687	3.211385
11	1	0	-2.172414	-0.134145	2.389371
12	1	0	-0.558551	-0.471381	3.009646
13	8	0	-1.088145	0.339309	-1.415207
14	1	0	-1.002760	-0.606027	-1.713894
15	1	0	-0.969490	-3.864083	-0.055178
16	8	0	-0.123257	-3.832883	-0.565790
17	1	0	0.473401	-3.263800	-0.017171
18	1	0	0.004259	1.187539	0.979171
19	8	0	1.439013	2.546970	0.605852
20	1	0	1.436917	3.473525	0.885975
21	1	0	2.389355	2.169022	0.809865
22	8	0	3.741225	1.523162	0.907582
23	1	0	4.015212	1.287882	1.806255
24	1	0	-0.515298	-2.863368	-1.811753
25	8	0	-0.815801	-2.187074	-2.506587
26	1	0	-0.144656	-2.196333	-3.205691
27	1	0	-3.486853	-1.335607	-1.757061
28	8	0	-3.680806	-1.960538	-2.487041
29	1	0	-2.783286	-2.247979	-2.749569
30	1	0	3.434517	0.106323	0.085399
31	8	0	3.321311	-0.711812	-0.495623
32	1	0	3.429723	-0.361084	-1.414174
33	1	0	4.879883	1.796021	-0.009280
34	8	0	5.627796	1.860010	-0.742812
35	1	0	5.975612	2.762952	-0.707332

36	1	0	-2.443574	-2.395990	0.459412
37	8	0	-2.514216	-3.334071	0.736616
38	1	0	-3.315480	-3.658759	0.258736
39	1	0	6.733878	0.586757	-0.160375
40	8	0	7.289343	-0.169217	0.161965
41	1	0	7.610919	-0.599727	-0.644183
42	1	0	4.626476	-1.715883	0.172523
43	8	0	5.283023	-2.153942	0.770787
44	1	0	6.028244	-1.515833	0.797256
45	1	0	1.967282	-1.526632	0.226403
46	8	0	1.325827	-1.962898	0.845983
47	1	0	1.907124	-2.278322	1.576964
48	1	0	3.413686	-3.788784	2.644919
49	8	0	3.382971	-2.820986	2.622363
50	1	0	4.161546	-2.554759	2.062438
51	1	0	-4.586548	-2.005617	1.883658
52	8	0	-5.032543	-1.422184	1.248503
53	1	0	-4.286237	-0.990628	0.773155
54	1	0	-5.314818	-2.937894	-0.099118
55	8	0	-4.948318	-3.661463	-0.646953
56	1	0	-4.609827	-3.201788	-1.451038
57	1	0	4.438776	1.336036	-2.232233
58	8	0	3.751484	0.852229	-2.737070
59	1	0	2.917252	1.367254	-2.627638
60	1	0	0.574392	1.426560	-2.122290
61	8	0	1.283371	2.095564	-2.134774
62	1	0	1.385132	2.327703	-1.179144
63	6	0	-1.895039	2.935561	-0.758150
64	6	0	-2.431779	4.210483	-0.576008
65	1	0	-4.181768	1.142086	1.004018
66	1	0	-5.125847	3.400936	1.334943
67	1	0	-4.006903	5.382810	0.321817
68	1	0	-0.998598	2.812402	-1.353724
69	1	0	-1.943522	5.065690	-1.036497
70	1	0	-0.128430	1.978296	3.326563
71	1	0	-1.740279	2.355491	2.685784
72	1	0	-1.579493	1.466835	4.211249

SCF Done: E(RB+HF-LYP) = -1778.53528511 A.U. after 1 cycles

Convg = 0.3375D-08 -V/T = 2.0093

S**2 = 0.0000

Zero-point correction= 0.608607 (a.u.)

Thermal correction to Energy= 0.658437

Thermal correction to Enthalpy= 0.659381

Thermal correction to Gibbs Free Energy= 0.522666

Sum of electronic and zero-point Energies= -1777.926678

Sum of electronic and thermal Energies= -1777.876848
Sum of electronic and thermal Enthalpies= -1777.875904
Sum of electronic and thermal Free Energies= -1778.012620

(4-7) Ph-COOH, H2N-Et and OH-(H2O)15
after the separation TS, Int3(am)
tukats3a.for.chk Stoichiometry C9H44NO18(1-)
Standard orientation:

Center Number	Atomic Number	Atomic Type	Coordinates (Angstroms) X	Y	Z
1	6	0	3.924412	-1.742043	-0.374241
2	6	0	3.267864	-0.455164	-0.734334
3	8	0	3.688204	0.632796	-0.343246
4	7	0	-0.336771	1.284013	2.085206
5	6	0	-0.860941	1.672280	3.406744
6	6	0	5.037708	-1.694749	0.478207
7	6	0	5.696233	-2.868347	0.833525
8	6	0	5.248095	-4.096867	0.339740
9	1	0	-0.981298	1.612875	1.359543
10	6	0	0.060939	1.195532	4.527877
11	1	0	-0.931714	2.766911	3.429201
12	1	0	-1.878618	1.284969	3.576594
13	8	0	2.194494	-0.576538	-1.521526
14	1	0	1.840929	0.326904	-1.806814
15	1	0	-0.451264	4.032268	-1.542706
16	8	0	-0.737261	3.180852	-1.959029
17	1	0	-1.386547	2.773701	-1.327342
18	1	0	-0.330828	0.261600	2.013912
19	8	0	-0.626344	-1.891677	1.820171
20	1	0	-0.358941	-2.536210	2.491310
21	1	0	-1.658275	-1.937858	1.771885
22	8	0	-3.170471	-1.998656	1.510605
23	1	0	-3.719446	-1.582639	2.191129
24	1	0	0.487836	2.254846	-2.269917
25	8	0	1.293145	1.702913	-2.589924
26	1	0	1.116367	1.526786	-3.526950
27	1	0	3.931374	2.407320	-1.303127
28	8	0	3.646788	3.162338	-1.850509
29	1	0	2.819417	2.821185	-2.250833
30	1	0	-3.173111	-1.014196	0.165878
31	8	0	-3.181790	-0.538837	-0.724611
32	1	0	-2.889147	-1.234570	-1.363434
33	1	0	-3.877423	-3.043325	0.742392
34	8	0	-4.330733	-3.710513	0.064125

35	1	0	-4.353351	-4.574994	0.499400
36	1	0	-0.436796	5.700328	0.068011
37	8	0	0.118500	5.507836	-0.701655
38	1	0	1.015048	5.261702	-0.328107
39	1	0	-5.908713	-2.970262	-0.295293
40	8	0	-6.755150	-2.517785	-0.547271
41	1	0	-6.847236	-2.687712	-1.496715
42	1	0	-4.873340	-0.113651	-0.955290
43	8	0	-5.818435	0.186358	-0.926336
44	1	0	-6.306797	-0.612659	-0.634519
45	1	0	-2.585227	1.080088	-0.426619
46	8	0	-2.430988	2.018764	-0.147858
47	1	0	-3.341432	2.376718	-0.032485
48	1	0	-5.447152	3.229324	-0.750018
49	8	0	-5.208695	2.674353	0.007093
50	1	0	-5.559768	1.773120	-0.224159
51	1	0	1.299818	1.971300	1.806679
52	8	0	2.221753	2.361958	1.699313
53	1	0	2.702483	1.745805	1.119329
54	1	0	2.366062	3.945167	0.850669
55	8	0	2.514017	4.722933	0.254414
56	1	0	2.988207	4.335883	-0.516138
57	1	0	-3.038211	-3.373838	-1.369577
58	8	0	-2.444360	-2.899044	-1.989104
59	1	0	-1.544228	-2.937416	-1.588858
60	1	0	0.568295	-1.930691	-1.087328
61	8	0	0.070827	-2.685752	-0.729967
62	1	0	-0.150789	-2.420098	0.200004
63	6	0	3.476049	-2.975949	-0.867362
64	6	0	4.139910	-4.148202	-0.507924
65	1	0	5.368407	-0.731039	0.850625
66	1	0	6.556953	-2.826550	1.495544
67	1	0	5.761319	-5.013963	0.618562
68	1	0	2.606984	-3.021359	-1.511683
69	1	0	3.784737	-5.102204	-0.887095
70	1	0	0.143660	0.101354	4.523471
71	1	0	1.067065	1.610497	4.405693
72	1	0	-0.327535	1.499169	5.507872

SCF Done: E(RB+HF-LYP) = -1778.56315896 A.U. after 1 cycles

Convg = 0.7820D-08 -V/T = 2.0094

S**2 = 0.0000

Zero-point correction= 0.606326 (a.u.)

Thermal correction to Energy= 0.657939

Thermal correction to Enthalpy= 0.658883

Thermal correction to Gibbs Free Energy= 0.513262

Sum of electronic and zero-point Energies= -1777.956833
Sum of electronic and thermal Energies= -1777.905220
Sum of electronic and thermal Enthalpies= -1777.904276
Sum of electronic and thermal Free Energies= -1778.049897

(4-8) TS4(am), the last TS leading to the benzoate ion
by the proton transfer from HO-
tukats4a.chk Stoichiometry C9H44NO18(1-)
Standard orientation:

Center Number	Atomic Number	Atomic Type	Coordinates (Angstroms) X	Y	Z
1	6	0	2.345740	2.904003	0.073873
2	6	0	2.784754	1.571736	0.601881
3	8	0	3.427534	0.783201	-0.104275
4	7	0	0.205785	-2.618635	-1.359206
5	6	0	-0.272325	-3.851730	-2.012484
6	6	0	2.756199	3.293149	-1.210105
7	6	0	2.366539	4.529724	-1.723926
8	6	0	1.564620	5.382876	-0.959816
9	1	0	-0.174608	-2.553899	-0.397622
10	1	0	0.192092	-3.912038	-3.005745
11	1	0	0.111631	-4.705034	-1.440991
12	6	0	-1.795301	-3.970319	-2.144287
13	8	0	2.436005	1.347340	1.845363
14	1	0	2.696402	0.383167	2.206178
15	1	0	1.899067	-3.327056	1.756263
16	8	0	1.495008	-2.739260	2.448460
17	1	0	0.518276	-2.481606	2.055607
18	1	0	-0.184710	-1.812573	-1.855398
19	8	0	-1.444181	-0.126959	-2.736057
20	1	0	-1.658975	-0.169579	-3.678969
21	1	0	-2.293162	-0.288394	-2.244397
22	8	0	-3.625144	-0.312013	-1.122452
23	1	0	-4.344974	-0.988387	-1.069378
24	1	0	2.252092	-1.787777	2.629016
25	8	0	3.039462	-0.903327	2.810337
26	1	0	3.151201	-0.809447	3.768373
27	1	0	4.889341	-0.396560	0.613178
28	8	0	5.284294	-1.102119	1.160738
29	1	0	4.601874	-1.199663	1.865453
30	1	0	-3.112463	-0.427073	-0.250000
31	8	0	-2.452380	-0.432184	1.225911
32	1	0	-2.073417	0.471233	1.303596
33	1	0	-4.303867	1.193909	-0.785750

34	8	0	-4.625308	2.083444	-0.447026
35	1	0	-4.748410	2.651686	-1.221215
36	1	0	1.822566	-3.653953	-0.345315
37	8	0	2.383167	-4.168254	0.267959
38	1	0	3.307788	-3.934955	-0.006651
39	1	0	-5.676489	1.869844	1.011186
40	8	0	-6.119757	1.634215	1.859031
41	1	0	-5.689791	2.182797	2.532116
42	1	0	-4.080023	-0.823164	1.931829
43	8	0	-5.042944	-1.017553	2.059081
44	1	0	-5.474733	-0.136579	2.092485
45	1	0	-1.693782	-1.154835	1.352146
46	8	0	-0.746187	-2.298362	1.440836
47	1	0	-1.282795	-3.032793	1.777153
48	1	0	-6.580926	-1.839322	-0.562470
49	8	0	-5.654036	-2.045594	-0.374326
50	1	0	-5.501242	-1.756825	0.572048
51	1	0	1.983701	-1.870298	-1.728145
52	8	0	2.845070	-1.447474	-1.971965
53	1	0	2.940545	-0.680823	-1.375710
54	1	0	4.216656	-2.577344	-1.313203
55	8	0	4.756021	-3.149743	-0.720271
56	1	0	5.070716	-2.528859	-0.021780
57	1	0	-2.912495	2.453905	0.487681
58	8	0	-1.996414	2.368716	0.815051
59	1	0	-1.456345	2.416088	-0.006435
60	1	0	0.213377	2.523123	-1.693226
61	8	0	-0.747348	2.395341	-1.743774
62	1	0	-0.889808	1.496536	-2.123216
63	6	0	1.538199	3.760647	0.835114
64	6	0	1.150502	4.995987	0.316779
65	1	0	3.381584	2.617072	-1.784277
66	1	0	2.686903	4.827438	-2.718860
67	1	0	1.256595	6.344205	-1.363279
68	1	0	1.208734	3.443843	1.817828
69	1	0	0.513884	5.649983	0.905870
70	1	0	-2.278487	-3.929950	-1.161617
71	1	0	-2.203817	-3.150430	-2.747944
72	1	0	-2.076651	-4.916923	-2.625116

SCF Done: E(RB3LYP) = -1778.54304325   A.U. after   1 cycles
Convg =   0.3577D-08   -V/T = 2.0093
Zero-point correction=   0.600546 (a.u.)
Thermal correction to Energy=   0.651295
Thermal correction to Enthalpy=   0.652239
Thermal correction to Gibbs Free Energy=   0.509907

Sum of electronic and zero-point Energies= -1777.942497
Sum of electronic and thermal Energies= -1777.891748
Sum of electronic and thermal Enthalpies= -1777.890804
Sum of electronic and thermal Free Energies= -1778.033136

(4-9) The product, Ph-COO-, H2N-Et and (H2O)16
tukats4a.for.chk Stoichiometry C9H44NO18(1-)

Standard orientation:

Center Number	Atomic Number	Atomic Type	Coordinates (Angstroms) X	Y	Z
1	6	0	3.039785	2.600811	-0.048224
2	6	0	3.292980	1.256315	0.619339
3	8	0	3.808549	0.345323	-0.094641
4	7	0	-0.030889	-2.413096	-1.375682
5	6	0	-0.486541	-3.347048	-2.421459
6	6	0	3.337127	2.789305	-1.404370
7	6	0	3.108304	4.024142	-2.013126
8	6	0	2.581920	5.085979	-1.272902
9	1	0	-0.551716	-2.571170	-0.507088
10	1	0	0.109491	-3.150926	-3.321311
11	1	0	-0.228450	-4.361565	-2.095571
12	6	0	-1.980146	-3.276084	-2.759445
13	8	0	2.969910	1.160790	1.837406
14	1	0	2.992197	-0.298820	2.584297
15	1	0	1.452475	-3.579406	1.749444
16	8	0	1.055716	-3.099051	2.523420
17	1	0	-0.427122	-2.676877	1.961371
18	1	0	-0.253489	-1.452022	-1.649885
19	8	0	-1.048658	0.550988	-2.083741
20	1	0	-0.926074	0.816128	-3.006989
21	1	0	-2.020433	0.429581	-1.942534
22	8	0	-3.633209	0.311149	-1.184368
23	1	0	-4.399832	-0.249096	-1.466216
24	1	0	1.692468	-2.379502	2.741662
25	8	0	3.034854	-1.181315	3.078477
26	1	0	3.181432	-0.951287	4.007417
27	1	0	4.795384	-0.961940	0.728679
28	8	0	5.166378	-1.743819	1.205558
29	1	0	4.576214	-1.804156	1.982940
30	1	0	-3.359739	-0.077420	-0.302724
31	8	0	-3.082064	-0.495851	1.334816
32	1	0	-2.735981	0.371847	1.673955
33	1	0	-4.240241	1.810403	-0.616862
34	8	0	-4.566335	2.618891	-0.125600

35	1	0	-4.393819	3.382776	-0.695420
36	1	0	1.384181	-3.598572	-0.406353
37	8	0	1.840148	-4.254594	0.159489
38	1	0	2.797859	-4.153276	-0.088034
39	1	0	-5.993053	2.192160	0.917233
40	8	0	-6.653320	1.797257	1.532079
41	1	0	-6.433943	2.150925	2.407078
42	1	0	-4.906876	-0.859399	1.487967
43	8	0	-5.873707	-0.932137	1.313158
44	1	0	-6.226428	-0.027634	1.467803
45	1	0	-2.390618	-1.191821	1.483797
46	8	0	-1.342263	-2.596849	1.536871
47	1	0	-1.819752	-3.385504	1.834462
48	1	0	-6.712244	-0.980182	-1.749236
49	8	0	-5.870592	-1.282477	-1.378782
50	1	0	-5.975098	-1.237598	-0.385735
51	1	0	1.944658	-1.794996	-1.682031
52	8	0	2.831492	-1.512699	-1.990128
53	1	0	3.113582	-0.802864	-1.369762
54	1	0	3.961612	-2.915458	-1.341485
55	8	0	4.355517	-3.589462	-0.741862
56	1	0	4.747573	-3.045511	-0.015275
57	1	0	-3.176240	2.480261	1.282226
58	8	0	-2.421681	2.120631	1.788026
59	1	0	-1.648760	2.300093	1.198935
60	1	0	0.347210	2.816504	-0.051249
61	8	0	-0.591056	2.639604	-0.237756
62	1	0	-0.610730	1.892070	-0.876970
63	6	0	2.512027	3.670985	0.689912
64	6	0	2.284767	4.907542	0.080847
65	1	0	3.751584	1.955937	-1.962043
66	1	0	3.341952	4.159074	-3.066758
67	1	0	2.403284	6.047757	-1.748228
68	1	0	2.294492	3.512125	1.741369
69	1	0	1.876039	5.730541	0.662325
70	1	0	-2.595884	-3.490483	-1.877715
71	1	0	-2.257447	-2.278430	-3.121302
72	1	0	-2.242279	-4.003308	-3.539213

SCF Done: E(RB3LYP) = -1778.57194124   A.U. after   1 cycles
Convg =   0.5257D-08   -V/T = 2.0094
Zero-point correction=   0.608726 (a.u.)
Thermal correction to Energy=   0.660999
Thermal correction to Enthalpy=   0.661943
Thermal correction to Gibbs Free Energy=   0.515024
Sum of electronic and zero-point Energies=   -1777.963215

Sum of electronic and thermal Energies= -1777.910942
Sum of electronic and thermal Enthalpies= -1777.909998
Sum of electronic and thermal Free Energies= -1778.056918

==

[5] The result of ADMP dynamical calculations starting from the n = 16 TS1(es) in Figures 1 and 3. The geometry after 800 femtoseconds shown in Figure S3.

TRJ-TRJ-TRJ-TRJ-TRJ-TRJ-TRJ-TRJ-TRJ-TRJ-TRJ-TRJ-TRJ-TRJ

INPUT DATA FOR L121

General parameters:
Maximum Steps = 8000
Random Number Generator Seed = 398465
Time Step = 0.10000 femtosec
Ficticious electronic mass = 0.10000 amu
MW individual basis funct. = True
Initial nuclear kin. energy = 0.10000 hartree
Initial electr. kin. energy = 0.00000 hartree
Initial electr. KE scheme = 0
Multitime step - NDtrC = 1
Multitime step - NDtrP = 1
No Thermostats chosen to control nuclear temperature
Integration parameters:
Follow Rxn Path (DVV) = False
Constraint Scheme = 10
Projection of angular mom. = True
Rotate density with nuclei = True

TRJ-TRJ-TRJ-TRJ-TRJ-TRJ-TRJ-TRJ-TRJ-TRJ-TRJ-TRJ-TRJ-TRJ-TRJ-TRJ-TRJ-TRJ
Symmetry turned off by external request.
Stoichiometry C9H43O19(1-)
{the geometry after 800 femtoseconds (1 femtosecond = 10**-15 seconds)}
Z-Matrix orientation:

Center Number	Atomic Number	Atomic Type	Coordinates (Angstroms) X	Y	Z
1	6	0	0.155426	3.218905	0.002278
2	6	0	-0.646047	2.589342	1.101693
3	8	0	-1.871292	2.518345	1.088164
4	8	0	0.075124	2.073706	2.088168
5	6	0	-0.595911	1.507661	3.252822
6	6	0	-0.416272	3.291273	-1.276570
7	6	0	0.296954	3.903214	-2.307088
8	6	0	1.409210	3.766710	0.267399
9	6	0	0.406709	1.663979	4.391734
10	1	0	-1.563735	2.059907	3.379593
11	1	0	-0.762703	0.498800	3.004392
12	8	0	2.816336	-0.173554	1.683389

13	1	0	2.209735	0.559851	1.484686
14	1	0	2.167136	-1.027442	1.661518
15	8	0	1.313067	-2.213730	1.261694
16	1	0	1.507436	-2.869930	1.937406
17	1	0	1.920583	-2.650400	-0.098705
18	8	0	2.439874	-2.905666	-0.967106
19	1	0	3.013158	-2.124998	-1.078767
20	1	0	3.727949	-3.304084	0.343191
21	8	0	4.309682	-3.064242	1.097955
22	1	0	3.913903	-2.251324	1.388929
23	1	0	1.312373	-2.568487	-2.210448
24	8	0	0.632085	-2.143044	-2.803458
25	1	0	-0.173855	-2.705488	-2.761394
26	1	0	0.109565	-0.874167	-1.940661
27	8	0	-0.210573	-0.036392	-1.430478
28	1	0	0.470751	0.657633	-1.384100
29	1	0	5.691134	-2.451894	0.228626
30	8	0	6.357180	-1.975578	-0.341479
31	1	0	6.497649	-2.609818	-1.066708
32	1	0	-3.039242	-0.008823	-1.052541
33	8	0	-2.815206	0.019488	-1.983876
34	1	0	-1.832790	0.103786	-1.960300
35	1	0	-2.281365	-1.871016	-2.637962
36	8	0	-2.020125	-2.793245	-2.738273
37	1	0	-2.413503	-3.154669	-1.939234
38	1	0	-4.747538	-0.696026	-2.192923
39	8	0	-5.479214	-1.087296	-1.653605
40	1	0	-5.400819	-0.661292	-0.783208
41	1	0	-0.998480	-1.167647	0.054654
42	8	0	-1.175027	-1.864633	0.691197
43	1	0	-0.247486	-2.147829	0.916200
44	1	0	-4.068531	-4.443679	2.165563
45	8	0	-4.775902	-3.791957	1.920986
46	1	0	-4.475020	-3.579291	0.950969
47	1	0	-2.883988	-2.484765	-0.003515
48	8	0	-3.634781	-2.971167	-0.386722
49	1	0	-4.236954	-2.286467	-0.756061
50	1	0	3.641435	-0.270137	0.024448
51	8	0	3.972294	-0.498072	-0.845719
52	1	0	4.949851	-0.703501	-0.755248
53	1	0	3.890378	1.131399	-1.687917
54	8	0	3.778614	1.999372	-2.050654
55	1	0	3.104196	1.854822	-2.686036
56	1	0	-3.577743	-2.350941	2.442126
57	8	0	-3.066596	-1.514112	2.539212
58	1	0	-2.252961	-1.618986	1.943870

59	1	0	-3.594694	-0.510124	1.263144
60	8	0	-4.033255	0.024472	0.527858
61	1	0	-3.800716	0.961021	0.653188
62	6	0	2.108074	4.428121	-0.721073
63	6	0	1.520000	4.509117	-1.990104
64	1	0	-1.397772	2.856362	-1.356030
65	1	0	-0.113251	3.985188	-3.340080
66	1	0	1.833899	3.759543	1.257922
67	1	0	3.075326	4.821603	-0.543185
68	1	0	2.137321	4.975422	-2.711986
69	1	0	1.385433	1.240245	4.061821
70	1	0	0.531687	2.707257	4.694864
71	1	0	0.074879	1.091183	5.236612

==

[6] Figure S2. The geometry of the Mulliken CT complex composed of benzoic acid, ethylamine and $(H_2O)_5$.

Stoichiometry C9H23NO7

(6-1) B3LYP/6-31(+)G(d) SCRF=(PCM, solvent=water)

Standard orientation:

Center Number	Atomic Number	Atomic Type	Coordinates (Angstroms) X	Y	Z
1	6	0	1.378914	0.023134	-0.547205
2	6	0	-0.145270	-0.023195	-0.400133
3	8	0	-0.787734	1.100493	-0.555506
4	7	0	-0.439871	-0.573994	1.147628
5	6	0	0.080117	0.269889	2.272742
6	6	0	2.045308	1.251447	-0.474904
7	6	0	3.435485	1.312912	-0.597999
8	6	0	4.175513	0.146236	-0.797221
9	1	0	-1.473870	-0.616798	1.216332
10	6	0	1.351675	-0.291678	2.898318
11	1	0	0.236945	1.273218	1.869617
12	1	0	-0.715836	0.336486	3.018729
13	8	0	-0.643004	-1.120753	-1.164171
14	1	0	-1.514256	-0.880986	-1.571619
15	1	0	-0.114161	-1.545282	1.225145
16	6	0	2.128244	-1.144734	-0.752334
17	6	0	3.516920	-1.083710	-0.875761
18	1	0	1.474530	2.161402	-0.328416
19	1	0	3.937852	2.274701	-0.541068
20	1	0	5.256616	0.194044	-0.894080
21	1	0	1.628406	-2.104661	-0.825676
22	1	0	4.082944	-1.996822	-1.037927
23	1	0	1.176803	-1.286889	3.323072
24	1	0	2.165715	-0.360204	2.171661
25	1	0	1.674517	0.366978	3.710757
26	8	0	-0.639103	-3.472836	0.355528
27	1	0	-0.693621	-2.839077	-0.392720
28	1	0	-0.109820	-4.220585	0.036973
29	8	0	-3.317812	-0.469181	1.641665
30	1	0	-3.586124	0.140010	0.913339
31	1	0	-3.813604	-1.290534	1.496896
32	8	0	-3.113129	-0.755855	-2.397756
33	1	0	-3.019803	-0.467445	-3.319746
34	1	0	-3.466816	0.021561	-1.905037
35	1	0	-2.433125	1.258785	-0.531835
36	8	0	-3.440495	1.210291	-0.536000
37	1	0	-3.778883	2.119564	-0.548447
38	1	0	-0.615256	2.777225	-0.008805

39	8	0	-0.624720	3.720150	0.288050
40	1	0	-0.493506	4.242986	-0.518044

(6-2) B3PW91/ 6-311+G(d,p) SCRF=(PCM, solvent=water)

Standard orientation:

Center Number	Atomic Number	Atomic Type	Coordinates (Angstroms) X	Y	Z
1	6	0	-1.338509	0.306952	0.190768
2	6	0	0.183042	0.158319	0.113469
3	8	0	0.784193	1.018905	-0.657224
4	7	0	0.474155	-1.334316	-0.465400
5	6	0	-0.084800	-1.656097	-1.809858
6	6	0	-2.000907	0.989736	-0.829173
7	6	0	-3.384981	1.140032	-0.798325
8	6	0	-4.121122	0.609987	0.257722
9	1	0	1.507998	-1.403054	-0.517435
10	6	0	-1.338660	-2.507845	-1.740585
11	1	0	-0.264616	-0.704249	-2.312586
12	1	0	0.698456	-2.174652	-2.365584
13	8	0	0.697632	0.046286	1.431667
14	1	0	1.566339	0.509760	1.480366
15	1	0	0.184038	-2.022740	0.238246
16	6	0	-2.081588	-0.218367	1.249885
17	6	0	-3.464937	-0.067080	1.283369
18	1	0	-1.419957	1.409998	-1.642249
19	1	0	-3.887355	1.674795	-1.597983
20	1	0	-5.199488	0.728127	0.285319
21	1	0	-1.580689	-0.736316	2.058857
22	1	0	-4.030688	-0.475016	2.114792
23	1	0	-1.135127	-3.469867	-1.261576
24	1	0	-2.138949	-2.006726	-1.192693
25	1	0	-1.695168	-2.708484	-2.753563
26	8	0	0.635159	-2.639725	2.200611
27	1	0	0.711661	-1.670230	2.251357
28	1	0	0.064927	-2.896984	2.930625
29	8	0	3.296670	-1.625392	-0.973289
30	1	0	3.555047	-0.681992	-1.031224
31	1	0	3.862874	-2.011523	-0.298444
32	8	0	3.179348	1.209358	1.766342
33	1	0	3.171878	2.085538	2.163430
34	1	0	3.473627	1.339285	0.841859
35	1	0	2.358614	1.104036	-0.921567
36	8	0	3.365910	1.098059	-0.952616

37	1	0	3.644562	1.718944	-1.631720
38	1	0	0.491739	2.735312	-0.442923
39	8	0	0.423128	3.713549	-0.351394
40	1	0	-0.520355	3.894112	-0.353902

----

[7] The Na^{+}-containing ester hydrolysis shown in Figure S3.

(7-1) Reactant-like complex (precursor) composed of ethyl benzoate and NaOH(H2O)16

zuka02aana.rev.chk Stoichiometry C9H43NaO19

Standard orientation:

Center Number	Atomic Number	Atomic Type	Coordinates (Angstroms) X	Y	Z
1	6	0	-3.388983	1.084531	-0.238450
2	6	0	-2.311023	0.885381	0.765159
3	8	0	-1.851612	-0.221371	1.075184
4	8	0	-1.892283	2.025436	1.294757
5	6	0	-0.750531	2.022834	2.198127
6	6	0	-3.490066	2.300178	-0.935378
7	6	0	-4.286689	0.038594	-0.507493
8	6	0	-5.282757	0.206205	-1.466944
9	6	0	-0.630785	3.423268	2.764910
10	1	0	-0.922276	1.271010	2.974200
11	1	0	0.132814	1.745474	1.615759
12	8	0	1.894450	0.641843	-1.302515
13	1	0	2.652481	0.617919	-1.923905
14	1	0	2.261343	0.465369	-0.356275
15	8	0	2.717642	0.205981	1.118014
16	1	0	2.735848	1.085475	1.536479
17	1	0	4.136370	-0.155804	0.923675
18	8	0	5.155663	-0.301630	0.690940
19	1	0	5.555201	-0.773370	1.437455
20	1	0	5.622590	1.376874	0.639269
21	8	0	5.711780	2.367914	0.649429
22	1	0	6.012940	2.620669	-0.235738
23	1	0	5.220295	-1.250663	-0.855889
24	8	0	5.164896	-1.736680	-1.713131
25	1	0	4.617928	-2.531860	-1.532928
26	1	0	4.227152	-0.874556	-2.810240
27	8	0	3.545948	-0.381622	-3.359519
28	1	0	4.018766	-0.020852	-4.124023
29	1	0	4.149565	2.967704	1.142789
30	8	0	3.231127	3.183582	1.456394
31	1	0	3.333079	3.883093	2.118603
32	1	0	1.305247	-0.787126	-1.902009
33	8	0	1.094471	-1.636323	-2.405239
34	1	0	1.638389	-1.524606	-3.209234
35	1	0	2.429778	-3.020543	-1.830332
36	8	0	3.109382	-3.652396	-1.521197

37	1	0	2.704866	-4.083840	-0.751471
38	1	0	-0.747735	-1.596664	-2.440227
39	8	0	-1.686111	-1.664373	-2.146513
40	1	0	-2.137682	-0.876517	-2.483578
41	1	0	0.017849	-0.744587	1.779461
42	8	0	0.823049	-1.274294	1.933697
43	1	0	1.632187	-0.659962	1.674424
44	1	0	0.462388	-2.397574	3.200096
45	8	0	0.007611	-3.079032	3.764885
46	1	0	0.610833	-3.833971	3.827595
47	1	0	0.646923	-2.487220	0.680153
48	8	0	0.290957	-3.109437	-0.007775
49	1	0	0.594249	-2.751007	-0.861667
50	1	0	1.490867	2.425855	-1.056097
51	8	0	1.426309	3.363237	-0.762349
52	1	0	2.031011	3.423942	0.007184
53	1	0	-0.164626	4.134656	-0.754499
54	8	0	-1.040330	4.596148	-0.769166
55	1	0	-0.909698	5.353431	-1.357957
56	1	0	-1.492751	-3.285656	2.935292
57	8	0	-2.352253	-3.174738	2.444087
58	1	0	-2.853914	-2.539089	2.976786
59	1	0	-3.449711	-4.032890	1.138784
60	8	0	-3.742020	-3.804454	0.232035
61	1	0	-3.972780	-4.628959	-0.218871
62	11	0	-1.905854	-2.370958	0.061921
63	6	0	-4.482974	2.450465	-1.903970
64	6	0	-5.378314	1.411853	-2.168867
65	1	0	-2.780829	3.101757	-0.743116
66	1	0	-4.225267	-0.892731	0.048120
67	1	0	-5.983564	-0.600090	-1.663333
68	1	0	-4.553886	3.384508	-2.453841
69	1	0	-6.152222	1.539701	-2.921085
70	1	0	-0.524323	4.153528	1.957870
71	1	0	-1.512827	3.683008	3.359218
72	1	0	0.250861	3.474529	3.413269

SCF Done: E(RB+HF-LYP) = -1960.70791006   A.U. after   1 cycles
Convg =   0.2288D-08   -V/T = 2.0090
S**2   =   0.0000

Zero-point correction=   0.590376 (a.u.)
Thermal correction to Energy=   0.645867
Thermal correction to Enthalpy=   0.646812
Thermal correction to Gibbs Free Energy=   0.493684
Sum of electronic and zero-point Energies=   -1960.117534
Sum of electronic and thermal Energies=   -1960.062043

Sum of electronic and thermal Enthalpies= -1960.061098
Sum of electronic and thermal Free Energies= -1960.214226

(7-2) TS1(es), OH- nucleophilic addition
zuka02aana.chk Stoichiometry C9H43NaO19
Standard orientation:

Center Number	Atomic Number	Atomic Type	Coordinates (Angstroms) X	Y	Z
1	6	0	0.307003	2.434064	-0.649981
2	6	0	-0.039039	1.416979	0.399734
3	8	0	-1.111310	0.726106	0.327400
4	8	0	0.465870	1.770505	1.597975
5	6	0	0.190790	0.947973	2.746176
6	6	0	1.450880	3.236588	-0.525369
7	6	0	-0.533882	2.604280	-1.757718
8	6	0	-0.238067	3.560779	-2.730258
9	6	0	0.813526	1.628865	3.951502
10	1	0	-0.893538	0.837768	2.860261
11	1	0	0.623702	-0.042796	2.578692
12	8	0	1.364728	0.042574	-0.066948
13	1	0	1.459602	-0.001125	-1.037381
14	1	0	1.352780	-1.422791	0.529554
15	8	0	1.335465	-2.344875	0.986139
16	1	0	1.951336	-2.266667	1.749352
17	1	0	2.211709	-3.342379	-0.088741
18	8	0	2.850575	-3.783706	-0.719985
19	1	0	2.878676	-4.721486	-0.476102
20	1	0	4.376333	-2.764187	-0.091233
21	8	0	4.940931	-2.218541	0.496471
22	1	0	4.707135	-1.281697	0.307410
23	1	0	2.029979	-3.539780	-2.358036
24	8	0	1.493255	-3.308313	-3.148191
25	1	0	0.579394	-3.608661	-2.944265
26	1	0	1.439290	-1.637557	-3.219106
27	8	0	1.271327	-0.648180	-3.152109
28	1	0	1.668727	-0.234600	-3.932385
29	1	0	4.159936	-2.202203	2.028810
30	8	0	3.504399	-1.961756	2.745050
31	1	0	3.800435	-2.381609	3.565841
32	1	0	-0.962030	-0.332835	-1.257498
33	8	0	-1.203846	-0.711448	-2.132589
34	1	0	-0.396857	-0.572487	-2.695143
35	1	0	-1.272146	-2.562024	-2.286126
36	8	0	-1.236488	-3.523409	-2.479851

37	1	0	-1.881299	-3.910900	-1.861914
38	1	0	-2.890572	-0.482181	-2.079582
39	8	0	-3.755887	-0.393960	-1.594094
40	1	0	-4.455372	-0.296553	-2.257172
41	1	0	-1.191842	-0.959464	1.090397
42	8	0	-1.315889	-1.897152	1.366054
43	1	0	-0.394586	-2.265387	1.338513
44	1	0	-2.887247	-2.205785	2.374631
45	8	0	-3.868369	-2.258479	2.423286
46	1	0	-4.082209	-2.729958	1.590193
47	1	0	-2.464321	-2.690492	0.167151
48	8	0	-3.274739	-3.038129	-0.274740
49	1	0	-3.605156	-2.284894	-0.800448
50	1	0	2.899772	0.325278	0.483661
51	8	0	3.832964	0.362917	0.875358
52	1	0	3.728315	0.020834	1.781537
53	1	0	4.339507	2.103914	0.943801
54	8	0	4.546532	3.067550	0.971050
55	1	0	5.464678	3.140876	0.673329
56	1	0	-4.321219	-0.663536	2.339607
57	8	0	-4.420716	0.334917	2.241025
58	1	0	-4.230066	0.696234	3.120000
59	1	0	-5.587926	1.577745	1.195836
60	8	0	-5.340789	2.122154	0.422273
61	1	0	-6.095212	2.682442	0.192184
62	11	0	-3.341114	0.999930	0.229325
63	6	0	1.743256	4.189217	-1.499166
64	6	0	0.901875	4.355883	-2.602297
65	1	0	2.120224	3.112790	0.317296
66	1	0	-1.413730	1.978947	-1.868271
67	1	0	-0.897065	3.681710	-3.586146
68	1	0	2.637084	4.796284	-1.390464
69	1	0	1.134562	5.100015	-3.359589
70	1	0	1.893080	1.746093	3.816955
71	1	0	0.378470	2.621146	4.109119
72	1	0	0.637763	1.024755	4.848561

SCF Done: E(RB+HF-LYP) = -1960.67759746 A.U. after 1 cycles

Convg = 0.3766D-08 -V/T = 2.0090

S**2 = 0.0000

Zero-point correction= 0.594015 (a.u.)

Thermal correction to Energy= 0.647392

Thermal correction to Enthalpy= 0.648336

Thermal correction to Gibbs Free Energy= 0.503230

Sum of electronic and zero-point Energies= -1960.083582

Sum of electronic and thermal Energies= -1960.030206

Sum of electronic and thermal Enthalpies= -1960.029262
Sum of electronic and thermal Free Energies= -1960.174368

(7-3) The OH- adduct, Int1(es)(anionic tetrahedral intermediate)
Ph-C(-O-)(OH)-OEt + Na+ + (H2O)16
zuka02bbbna.rev.chk Stoichiometry C9H43NaO19
Standard orientation:

Center Number	Atomic Number	Atomic Type	Coordinates (Angstroms) X	Y	Z
1	6	0	-0.939830	2.605122	-0.499605
2	6	0	-1.493055	1.172981	-0.608753
3	8	0	-1.272958	0.463764	0.526088
4	8	0	-0.794882	0.427194	-1.681365
5	6	0	-1.021186	0.895391	-3.033739
6	6	0	-1.748580	3.604562	0.058031
7	6	0	0.384281	2.919901	-0.830617
8	6	0	0.894123	4.199960	-0.600066
9	6	0	-0.053883	0.182859	-3.964982
10	1	0	-2.061355	0.689763	-3.304049
11	1	0	-0.869663	1.979919	-3.067645
12	8	0	-2.852544	1.261293	-0.947596
13	1	0	-3.343232	0.508789	-0.553115
14	1	0	-4.872912	-3.430731	-1.276191
15	8	0	-4.732802	-2.514010	-1.554578
16	1	0	-3.762347	-2.433469	-1.766643
17	1	0	0.922775	-0.126772	-1.155684
18	8	0	2.676310	1.029841	1.486470
19	1	0	1.824663	1.347740	1.875396
20	1	0	2.887379	0.232203	2.014796
21	8	0	2.507348	-1.535128	2.793493
22	1	0	3.157680	-1.883959	3.422000
23	1	0	-4.909224	-1.286519	-0.391838
24	8	0	-4.849650	-0.493422	0.210116
25	1	0	-5.615682	0.062279	0.001041
26	1	0	-2.552232	0.289945	1.749155
27	8	0	-3.204957	0.004744	2.439971
28	1	0	-3.966154	-0.302217	1.906129
29	1	0	2.690517	-2.003081	1.917151
30	8	0	2.880282	-2.538894	0.359649
31	1	0	3.826475	-2.522328	0.052095
32	1	0	5.378856	2.619285	0.420472
33	8	0	4.581850	2.348101	-0.057485
34	1	0	3.956146	1.996602	0.615917
35	1	0	-0.957159	-1.175078	0.726215

36	8	0	-0.745815	-2.027212	1.218041
37	1	0	-1.614661	-2.427100	1.380485
38	1	0	5.825087	-2.664788	-1.208474
39	8	0	5.393963	-2.075362	-0.572158
40	1	0	5.179014	-1.236233	-1.070529
41	1	0	1.573641	-3.869709	0.068755
42	8	0	0.649709	-4.185529	-0.022179
43	1	0	0.124080	-3.450350	0.363941
44	1	0	-1.625558	-1.324940	-1.788027
45	8	0	-2.083952	-2.187755	-1.861477
46	1	0	-1.431650	-2.841401	-2.211608
47	1	0	-0.457362	-4.916626	-2.933193
48	8	0	-0.190627	-4.051905	-2.589330
49	1	0	0.181727	-4.209129	-1.678966
50	1	0	-0.236603	1.889657	3.004690
51	8	0	0.170765	1.158183	2.516752
52	1	0	-0.400361	1.028908	1.667945
53	1	0	-2.098757	-1.299918	5.135041
54	8	0	-1.735837	-1.332600	4.239125
55	1	0	-2.398646	-0.875947	3.644121
56	1	0	4.672354	0.967129	-1.303807
57	8	0	4.506713	0.148645	-1.824371
58	1	0	3.536789	0.019669	-1.766585
59	1	0	2.434046	-1.837945	-0.182216
60	8	0	1.848894	-0.305621	-0.866324
61	1	0	2.015440	0.268775	-0.084034
62	11	0	0.222065	-1.112551	3.110585
63	6	0	-1.239777	4.883045	0.292252
64	6	0	0.084322	5.185185	-0.032984
65	1	0	-2.780381	3.371456	0.298288
66	1	0	1.024894	2.170963	-1.283624
67	1	0	1.924102	4.420084	-0.867825
68	1	0	-1.882946	5.646487	0.723023
69	1	0	0.479200	6.181788	0.146401
70	1	0	-0.181111	-0.904099	-3.918771
71	1	0	-0.239546	0.500678	-4.997227
72	1	0	0.987006	0.419632	-3.719404

SCF Done: E(RB+HF-LYP) = -1960.70487010 A.U. after 1 cycles

Convg = 0.1911D-08 -V/T = 2.0090

S**2 = 0.0000

Zero-point correction= 0.596904 (a.u.)

Thermal correction to Energy= 0.648209

Thermal correction to Enthalpy= 0.649154

Thermal correction to Gibbs Free Energy= 0.510905

Sum of electronic and zero-point Energies= -1960.107966

Sum of electronic and thermal Energies= -1960.056661
Sum of electronic and thermal Enthalpies= -1960.055717
Sum of electronic and thermal Free Energies= -1960.193966

(7-4) TS2(es), formation of EtOH, PhCOOH + NaOH(H2O)15
zuka02bbbna.chk Stoichiometry C9H43NaO19
Standard orientation:

Center Number	Atomic Number	Atomic Type	Coordinates (Angstroms) X	Y	Z
1	6	0	0.264441	2.916402	-0.232370
2	6	0	1.257926	1.797084	-0.243932
3	8	0	1.159125	0.825579	-1.073173
4	8	0	0.635771	0.830893	1.431269
5	6	0	0.526279	1.645891	2.587959
6	6	0	0.589225	4.159294	0.329195
7	6	0	-1.000322	2.740334	-0.807394
8	6	0	-1.932016	3.776178	-0.812193
9	6	0	0.046353	0.856105	3.808637
10	1	0	1.503749	2.105209	2.808844
11	1	0	-0.180909	2.468332	2.401239
12	8	0	2.479006	2.185882	0.162073
13	1	0	3.165742	1.511820	-0.064035
14	1	0	5.482759	-1.570803	1.725637
15	8	0	5.076656	-0.691776	1.743120
16	1	0	4.105769	-0.837641	1.931041
17	1	0	-0.817335	0.172993	1.074750
18	8	0	-2.692156	-0.401145	-1.982327
19	1	0	-1.950520	-0.168428	-2.582546
20	1	0	-2.680122	-1.380400	-1.978241
21	8	0	-1.820411	-3.147246	-1.840365
22	1	0	-2.309793	-3.896902	-2.212250
23	1	0	4.986019	0.144147	0.289754
24	8	0	4.759444	0.692146	-0.516380
25	1	0	5.420904	1.399863	-0.557453
26	1	0	2.520684	0.293141	-2.204987
27	8	0	3.237797	-0.079116	-2.767764
28	1	0	4.012895	-0.017598	-2.173387
29	1	0	-2.031436	-3.144281	-0.850013
30	8	0	-2.303888	-2.880519	0.747938
31	1	0	-3.260141	-2.888675	1.021968
32	1	0	-5.693636	0.942603	-1.624331
33	8	0	-4.860014	1.154656	-1.179600
34	1	0	-4.169902	0.595600	-1.602791
35	1	0	1.063858	-0.892108	-0.472574

36	8	0	1.236812	-1.859165	-0.366078
37	1	0	1.866000	-1.827916	0.398432
38	1	0	-5.286512	-2.761571	2.236269
39	8	0	-4.917775	-2.472563	1.388530
40	1	0	-4.853511	-1.474935	1.439483
41	1	0	-0.833811	-3.802958	1.364416
42	8	0	0.129664	-3.998296	1.381131
43	1	0	0.487027	-3.342186	0.750956
44	1	0	1.772745	-0.327180	1.820711
45	8	0	2.432104	-1.092440	1.936770
46	1	0	2.083560	-1.676339	2.653869
47	1	0	1.841405	-3.659512	4.097692
48	8	0	1.330838	-2.993786	3.614923
49	1	0	0.840544	-3.474665	2.897372
50	1	0	0.209302	0.043461	-3.934952
51	8	0	-0.185653	-0.218469	-3.088714
52	1	0	0.242186	0.353543	-2.388821
53	1	0	2.767422	-2.907516	-4.255614
54	8	0	2.158542	-2.359524	-3.741568
55	1	0	2.683989	-1.576840	-3.409965
56	1	0	-4.714001	0.616143	0.617708
57	8	0	-4.452106	0.181811	1.459790
58	1	0	-3.469115	0.197192	1.448271
59	1	0	-2.011540	-1.935291	0.890467
60	8	0	-1.719650	-0.243227	0.840172
61	1	0	-1.884701	-0.067001	-0.106162
62	11	0	0.363636	-2.451241	-2.361502
63	6	0	-0.339724	5.199915	0.316461
64	6	0	-1.601747	5.011304	-0.249161
65	1	0	1.569363	4.304924	0.768095
66	1	0	-1.259096	1.786044	-1.249338
67	1	0	-2.913527	3.612502	-1.248004
68	1	0	-0.076053	6.159609	0.752821
69	1	0	-2.326092	5.821373	-0.250511
70	1	0	-0.935428	0.406276	3.622159
71	1	0	0.741682	0.052042	4.073875
72	1	0	-0.045775	1.522974	4.675485

SCF Done: E(RB+HF-LYP) = -1960.69501668   A.U. after   1 cycles
Convg =   0.2003D-08   -V/T = 2.0090
S**2   =   0.0000
Zero-point correction=   0.595022 (a.u.)
Thermal correction to Energy=   0.646997
Thermal correction to Enthalpy=   0.647941
Thermal correction to Gibbs Free Energy=   0.507690
Sum of electronic and zero-point Energies=   -1960.099995

Sum of electronic and thermal Energies= -1960.048019
Sum of electronic and thermal Enthalpies= -1960.047075
Sum of electronic and thermal Free Energies= -1960.187326

(7-5) Ph-COOH + EtOH + NaOH(H2O)15 Int2(es)
zuka02bbbna.for.chk Stoichiometry C9H43NaO19
Standard orientation:

Center Number	Atomic Number	Atomic Type	Coordinates (Angstroms) X	Y	Z
1	6	0	-0.854018	2.991336	-0.585219
2	6	0	-2.030787	2.312112	-0.003303
3	8	0	-2.091434	1.929194	1.178325
4	8	0	-0.033532	-0.328770	-2.745544
5	6	0	0.745953	-0.419190	-3.941071
6	6	0	-0.805551	3.290197	-1.956456
7	6	0	0.245747	3.297046	0.233326
8	6	0	1.386759	3.878497	-0.312404
9	6	0	1.604723	-1.679355	-4.000044
10	1	0	0.030323	-0.401059	-4.770692
11	1	0	1.376461	0.476789	-4.029809
12	8	0	-3.035321	2.131844	-0.848206
13	1	0	-3.745550	1.534300	-0.475447
14	1	0	-4.961317	-2.460160	-1.719194
15	8	0	-4.539827	-1.603578	-1.882161
16	1	0	-3.561506	-1.793544	-1.978529
17	1	0	0.574809	-0.278849	-1.944496
18	8	0	2.436430	0.984222	3.297074
19	1	0	1.488677	1.222526	3.286624
20	1	0	2.422123	0.004945	3.244550
21	8	0	1.813071	-1.809457	3.081044
22	1	0	2.196096	-2.395305	3.751272
23	1	0	-4.851505	-0.385158	-0.801194
24	8	0	-5.007699	0.381915	-0.169563
25	1	0	-5.870498	0.754324	-0.409497
26	1	0	-3.388605	0.933521	2.087491
27	8	0	-4.013876	0.295415	2.493538
28	1	0	-4.576034	0.047871	1.732884
29	1	0	2.244581	-2.071681	2.198170
30	8	0	2.739698	-2.326412	0.684084
31	1	0	3.709304	-2.215542	0.514757
32	1	0	4.814537	2.452636	1.785608
33	8	0	3.943721	2.296703	1.390787
34	1	0	3.423464	1.813563	2.082233
35	1	0	0.162251	-1.158793	0.081002

36	8	0	-0.596834	-1.699517	0.507724
37	1	0	-1.249021	-1.813023	-0.221526
38	1	0	5.792861	-2.055912	-0.669067
39	8	0	5.314287	-1.598409	0.037491
40	1	0	4.968839	-0.748790	-0.361962
41	1	0	1.592838	-3.828632	0.181441
42	8	0	0.676032	-4.175354	0.138715
43	1	0	0.151790	-3.371850	0.367815
44	1	0	-1.266415	-1.445652	-2.365801
45	8	0	-1.903051	-2.090657	-1.940235
46	1	0	-1.539055	-2.989037	-2.140117
47	1	0	-0.949770	-5.386981	-2.307042
48	8	0	-0.575862	-4.496364	-2.244569
49	1	0	-0.016392	-4.483617	-1.419515
50	1	0	-0.934611	1.171466	3.954845
51	8	0	-0.433695	0.992782	3.143173
52	1	0	-0.940475	1.432094	2.416956
53	1	0	-2.912554	-2.058034	4.394003
54	8	0	-2.421859	-1.446301	3.827647
55	1	0	-3.099060	-0.884940	3.358917
56	1	0	4.051506	1.238247	-0.158570
57	8	0	4.043918	0.585047	-0.894002
58	1	0	3.112662	0.249019	-0.919069
59	1	0	2.294276	-1.556252	0.202008
60	8	0	1.406313	-0.342454	-0.492924
61	1	0	1.301202	0.503448	-0.028468
62	11	0	-0.445083	-1.345807	2.700060
63	6	0	0.331573	3.886535	-2.495505
64	6	0	1.427792	4.173588	-1.678673
65	1	0	-1.647318	3.034933	-2.588937
66	1	0	0.205588	3.077386	1.294815
67	1	0	2.246220	4.075752	0.320012
68	1	0	0.368684	4.111896	-3.557251
69	1	0	2.321195	4.619433	-2.106704
70	1	0	2.333971	-1.688499	-3.182123
71	1	0	0.987406	-2.580632	-3.916646
72	1	0	2.156947	-1.721714	-4.947492

SCF Done: E(RB+HF-LYP) = -1960.72805199 A.U. after 1 cycles
Convg = 0.6559D-08 -V/T = 2.0091
S**2 = 0.0000

Zero-point correction= 0.595573 (a.u.)
Thermal correction to Energy= 0.648765
Thermal correction to Enthalpy= 0.649710
Thermal correction to Gibbs Free Energy= 0.503345
Sum of electronic and zero-point Energies= -1960.132479

Sum of electronic and thermal Energies= -1960.079287
Sum of electronic and thermal Enthalpies= -1960.078342
Sum of electronic and thermal Free Energies= -1960.224707

(7-6) The electrolytic dissociation of Ph-COOH, TS3(es),
the last step formation of the benzoate anion
zukaphxnr.chk Stoichiometry C9H43NaO19
Standard orientation:

Center Number	Atomic Number	Atomic Type	Coordinates (Angstroms) X	Y	Z
1	6	0	-3.966833	1.204400	-0.337315
2	6	0	-3.128389	0.276158	-1.148590
3	8	0	-3.659693	-0.511334	-1.965825
4	8	0	0.712398	-1.722139	2.029541
5	6	0	1.284255	-2.242737	3.241788
6	6	0	-5.178539	1.672897	-0.867483
7	6	0	-5.972250	2.545033	-0.125863
8	6	0	-5.571759	2.933098	1.156071
9	1	0	1.193355	-1.490323	4.035969
10	1	0	2.353349	-2.445997	3.084032
11	6	0	0.548026	-3.514042	3.627848
12	8	0	-1.844980	0.348396	-0.950741
13	1	0	0.790563	1.526236	-1.457635
14	1	0	-1.198817	-0.436070	-1.503114
15	8	0	-0.336813	-1.195064	-2.093413
16	1	0	-0.819893	-1.584231	-2.862457
17	1	0	-1.123473	-1.143374	2.360783
18	8	0	-1.977530	-0.770595	2.651136
19	1	0	-2.659800	-1.402439	2.343316
20	8	0	0.460353	2.014927	-0.632655
21	1	0	1.316011	1.145833	0.679574
22	1	0	-0.483841	1.765250	-0.566799
23	8	0	1.347418	0.485332	-2.590009
24	1	0	0.463177	-0.454584	-2.442779
25	1	0	1.396917	0.797466	-3.505204
26	1	0	0.481253	-2.444986	-1.052273
27	8	0	0.950716	-3.050890	-0.428980
28	1	0	0.821964	-2.657150	0.465661
29	8	0	1.950003	0.589423	1.200152
30	1	0	1.158408	-0.869028	1.800606
31	1	0	2.403214	1.193629	1.839259
32	1	0	-2.869523	-1.464471	-3.284748
33	8	0	-2.266186	-1.944924	-3.901460
34	1	0	-2.590971	-2.856780	-3.936525

35	8	0	3.332936	2.351976	2.803377
36	1	0	2.815500	2.893390	3.417854
37	1	0	3.725290	2.972608	2.145000
38	8	0	4.253881	3.887959	0.673613
39	1	0	3.400272	4.091015	0.196908
40	1	0	4.696466	4.739038	0.812000
41	1	0	-4.857796	-1.628458	-1.185969
42	8	0	-5.356830	-2.296525	-0.661589
43	1	0	-6.222931	-1.900426	-0.484513
44	1	0	-4.435882	-2.566436	0.881547
45	8	0	-3.928615	-2.703946	1.715909
46	1	0	-3.613073	-3.618528	1.679738
47	1	0	2.707348	0.182231	-1.769383
48	8	0	3.435080	0.049933	-1.077702
49	1	0	2.973084	0.207585	-0.216785
50	1	0	1.253075	3.561069	-0.513407
51	8	0	1.840338	4.362123	-0.470282
52	1	0	1.779401	4.778520	-1.342688
53	11	0	4.751515	-1.736215	-0.948532
54	8	0	6.636007	-0.578739	-0.540176
55	1	0	7.597916	-0.643514	-0.465460
56	1	0	6.410009	0.392511	-0.602031
57	8	0	3.505974	-3.557284	-0.885139
58	1	0	2.523302	-3.368386	-0.741173
59	1	0	3.582244	-4.518222	-0.964312
60	8	0	5.522432	1.826847	-0.961152
61	1	0	4.677431	1.337732	-1.118850
62	1	0	5.277289	2.553500	-0.353497
63	6	0	-3.565805	1.591968	0.949960
64	6	0	-4.377641	2.448463	1.693902
65	1	0	-5.477565	1.360849	-1.863710
66	1	0	-6.901647	2.921289	-0.544906
67	1	0	-6.196339	3.606046	1.738018
68	1	0	-2.666211	1.168205	1.386717
69	1	0	-4.079047	2.727261	2.700390
70	1	0	-0.510690	-3.303583	3.810567
71	1	0	0.623437	-4.264417	2.832934
72	1	0	0.979055	-3.936735	4.542467

SCF Done: E(RB+HF-LYP) = -1960.71330345   A.U. after   2 cycles
Convg =   0.7316D-08   -V/T = 2.0090
S**2   =   0.0000

Zero-point correction=   0.587591 (a.u.)
Thermal correction to Energy=   0.641530
Thermal correction to Enthalpy=   0.642475
Thermal correction to Gibbs Free Energy=   0.491106

Sum of electronic and zero-point Energies= -1960.125713
Sum of electronic and thermal Energies= -1960.071773
Sum of electronic and thermal Enthalpies= -1960.070829
Sum of electronic and thermal Free Energies= -1960.222198

(7-7) Product of Ph-COO-, EtOH, Na+ and (H2O)16, i.e., after the last step formation of the benzoate anion
zukaphxnr.for.chk Stoichiometry C9H43NaO19

Standard orientation:

Center Number	Atomic Number	Atomic Type	Coordinates (Angstroms) X	Y	Z
1	6	0	-3.827094	1.060530	-0.440231
2	6	0	-3.032509	-0.006675	-1.142231
3	8	0	-3.667233	-0.905291	-1.774267
4	8	0	0.818185	-1.845972	1.845287
5	6	0	1.393965	-2.218747	3.110160
6	6	0	-5.038568	1.503655	-0.990081
7	6	0	-5.772768	2.505019	-0.355368
8	6	0	-5.314879	3.050969	0.846845
9	1	0	1.203282	-1.422408	3.840613
10	1	0	2.482573	-2.331295	2.999080
11	6	0	0.764276	-3.522254	3.568671
12	8	0	-1.763168	0.049983	-1.065950
13	1	0	0.598967	1.436134	-1.743014
14	1	0	-0.777784	-1.148457	-1.730344
15	8	0	-0.156153	-1.729727	-2.263921
16	1	0	-0.790605	-2.197928	-2.865725
17	1	0	-0.990865	-1.133964	2.325539
18	8	0	-1.685347	-0.620469	2.777104
19	1	0	-2.519786	-1.111168	2.621939
20	8	0	0.209193	1.783478	-0.909661
21	1	0	1.166366	1.002401	0.380674
22	1	0	-0.671588	1.307948	-0.850935
23	8	0	1.365279	0.281846	-3.052643
24	1	0	0.796129	-0.537663	-2.898384
25	1	0	1.309661	0.494617	-3.996303
26	1	0	0.819899	-2.803630	-1.161286
27	8	0	1.366131	-3.275359	-0.487077
28	1	0	1.121783	-2.866144	0.377242
29	8	0	1.879595	0.525117	0.877813
30	1	0	1.193310	-0.977534	1.561273
31	1	0	2.213108	1.153720	1.568520
32	1	0	-2.917238	-1.961840	-2.939868
33	8	0	-2.399218	-2.545138	-3.561035

34	1	0	-2.771299	-3.433432	-3.458531
35	8	0	2.864102	2.345595	2.676449
36	1	0	2.191196	2.721611	3.263923
37	1	0	3.175982	3.080041	2.098850
38	8	0	3.620864	4.133009	0.679069
39	1	0	2.744318	4.254688	0.220764
40	1	0	3.969678	5.021988	0.844391
41	1	0	-4.867511	-1.711868	-0.758660
42	8	0	-5.451899	-2.153093	-0.091111
43	1	0	-6.223794	-1.573694	-0.007582
44	1	0	-4.560804	-2.154988	1.467144
45	8	0	-4.084062	-2.151124	2.333390
46	1	0	-4.079987	-3.071502	2.633029
47	1	0	2.844413	0.188550	-2.044855
48	8	0	3.486943	0.208443	-1.296142
49	1	0	2.923800	0.325422	-0.482747
50	1	0	0.686249	3.416805	-0.573836
51	8	0	1.121254	4.284173	-0.359739
52	1	0	0.732827	4.942988	-0.952947
53	11	0	4.956758	-1.400259	-0.750542
54	8	0	6.531922	0.029121	-0.051781
55	1	0	7.424538	0.089187	0.315370
56	1	0	6.215897	0.962291	-0.215170
57	8	0	3.983689	-3.375337	-0.773505
58	1	0	2.974564	-3.346804	-0.698288
59	1	0	4.219977	-4.312846	-0.804065
60	8	0	5.280166	2.279807	-0.821593
61	1	0	4.557120	1.709954	-1.169219
62	1	0	4.829244	2.953645	-0.271432
63	6	0	-3.369736	1.610352	0.766059
64	6	0	-4.120722	2.594955	1.409395
65	1	0	-5.386724	1.065788	-1.921028
66	1	0	-6.701931	2.857765	-0.795869
67	1	0	-5.892980	3.823518	1.347785
68	1	0	-2.463269	1.224183	1.223651
69	1	0	-3.775039	2.998610	2.357199
70	1	0	-0.315189	-3.398656	3.701120
71	1	0	0.937081	-4.319031	2.836681
72	1	0	1.196351	-3.833460	4.526447

SCF Done: E(RB+HF-LYP) = -1960.72927973 A.U. after 1 cycles

Convg = 0.3955D-08 -V/T = 2.0090

S**2 = 0.0000

Zero-point correction= 0.594166 (a.u)

Thermal correction to Energy= 0.649193

Thermal correction to Enthalpy= 0.650137

Thermal correction to Gibbs Free Energy= 0.495482
Sum of electronic and zero-point Energies= -1960.135114
Sum of electronic and thermal Energies= -1960.080087
Sum of electronic and thermal Enthalpies= -1960.079143
Sum of electronic and thermal Free Energies= -1960.233798

==

[8] The Na^+-containing amide hydrolysis shown in Figure S4.

(8-1) Reactant-like complex (precursor) composed of
N-ethyl benzamide and NaOH(H2O)16
tuka02aana.rev.chk Stoichiometry C9H44NNaO18
standard orientation:

1	6	0	2.204289	1.953649	0.663871
2	6	0	1.636890	1.521096	-0.655192
3	8	0	1.973942	0.405934	-1.142297
4	7	0	0.799702	2.360766	-1.263101
5	6	0	0.138148	2.099224	-2.544111
6	6	0	1.383618	2.446986	1.687239
7	6	0	3.582425	1.812315	0.886744
8	6	0	4.136364	2.174988	2.113843
9	1	0	0.540199	3.241249	-0.801229
10	6	0	0.387100	3.233031	-3.539564
11	1	0	0.517379	1.151044	-2.927638
12	1	0	-0.935674	1.981507	-2.357706
13	8	0	-1.738903	0.609911	1.144823
14	1	0	-2.382778	0.349146	1.838150
15	1	0	-2.031675	0.146298	0.269314
16	8	0	-2.393217	-0.525219	-1.087587
17	1	0	-2.707315	0.195540	-1.660897
18	1	0	-3.592077	-1.315022	-0.779296
19	8	0	-4.500763	-1.782953	-0.501510
20	1	0	-4.641810	-2.515702	-1.120353
21	1	0	-5.577011	-0.465841	-0.768433
22	8	0	-6.054796	0.388786	-0.957963
23	1	0	-6.476791	0.649516	-0.126004
24	1	0	-4.231667	-2.407506	1.188931
25	8	0	-3.975714	-2.717921	2.089857
26	1	0	-3.190253	-3.297519	1.946276
27	1	0	-3.346278	-1.457859	2.971287
28	8	0	-2.858922	-0.692565	3.411497
29	1	0	-3.418044	-0.398972	4.145869
30	1	0	-4.828108	1.461152	-1.548474
31	8	0	-4.056128	1.995423	-1.879706
32	1	0	-4.389514	2.508993	-2.630283
33	1	0	-0.629180	-0.555808	1.818938
34	8	0	-0.242687	-1.259386	2.413908
35	1	0	-0.789694	-1.165892	3.218391
36	1	0	-1.035491	-2.975042	1.998939
37	8	0	-1.450795	-3.844658	1.822037
38	1	0	-1.034575	-4.118758	0.978832
39	1	0	1.290207	-1.871653	1.964238

40	8	0	2.079119	-2.293369	1.523281
41	1	0	2.564210	-2.751614	2.225824
42	1	0	0.490271	-0.821434	-1.657110
43	8	0	-0.146752	-1.548633	-1.801281
44	1	0	-1.079142	-1.148278	-1.584089
45	1	0	0.645121	-2.826686	-2.928414
46	8	0	1.270547	-3.576995	-3.067192
47	1	0	1.085258	-4.134437	-2.283993
48	1	0	0.106065	-2.955638	-0.745093
49	8	0	0.397635	-3.819869	-0.351899
50	1	0	1.048488	-3.565059	0.329834
51	1	0	-2.314783	2.277040	0.677967
52	8	0	-2.684065	3.121216	0.321576
53	1	0	-3.213983	2.844058	-0.459024
54	1	0	-1.425873	4.175166	-0.093917
55	8	0	-0.598799	4.664752	-0.371051
56	1	0	-0.499165	5.408591	0.239793
57	1	0	2.739310	-2.872481	-2.757816
58	8	0	3.545348	-2.339599	-2.468406
59	1	0	3.722164	-1.726105	-3.197715
60	1	0	4.984998	-2.659375	-1.249859
61	8	0	5.194804	-2.308867	-0.358518
62	1	0	5.956795	-2.795975	-0.015218
63	11	0	3.084697	-1.344755	-0.318836
64	6	0	1.941459	2.792206	2.919668
65	6	0	3.314598	2.661927	3.133880
66	1	0	0.312653	2.524881	1.531384
67	1	0	4.221354	1.447922	0.085514
68	1	0	5.207683	2.084245	2.271465
69	1	0	1.298056	3.158362	3.714620
70	1	0	3.744743	2.938724	4.092523
71	1	0	0.030603	4.187966	-3.140467
72	1	0	1.454727	3.328015	-3.766772
73	1	0	-0.147182	3.029733	-4.474206

SCF Done: E(RB+HF-LYP) = -1940.85272273   A.U. after   1 cycles
Convg =   0.4247D-08   -V/T = 2.0091
S**2   =   0.0000
Zero-point correction=   0.606447 (a.u.)
Thermal correction to Energy=   0.660246
Thermal correction to Enthalpy=   0.661190
Thermal correction to Gibbs Free Energy=   0.513787
Sum of electronic and zero-point Energies=   -1940.246275
Sum of electronic and thermal Energies=   -1940.192477
Sum of electronic and thermal Enthalpies=   -1940.191532
Sum of electronic and thermal Free Energies=   -1940.338935

(8-2) TS1(am), OH- nucleophilic addition to the amide

tuka02aana.chk Stoichiometry C9H44NNaO18

Standard orientation:

Center Number	Atomic Number	Atomic Type	Coordinates (Angstroms) X	Y	Z
1	6	0	0.403379	2.335923	1.008445
2	6	0	0.372184	1.347645	-0.151082
3	8	0	1.144645	0.290813	-0.067036
4	7	0	0.284650	1.971109	-1.384197
5	6	0	0.235892	1.204183	-2.627991
6	6	0	-0.548705	3.358721	1.136122
7	6	0	1.425911	2.253064	1.960551
8	6	0	1.501630	3.165908	3.015343
9	1	0	-0.323783	2.788791	-1.413459
10	6	0	0.509204	2.109377	-3.828406
11	1	0	0.987948	0.412987	-2.571393
12	1	0	-0.740864	0.711558	-2.747194
13	8	0	-1.338097	0.648278	0.123234
14	1	0	-1.423954	0.465546	1.080531
15	1	0	-1.842981	-0.661631	-0.615053
16	8	0	-2.176464	-1.480843	-1.138284
17	1	0	-2.828981	-1.125414	-1.779541
18	1	0	-3.072689	-2.507410	-0.182803
19	8	0	-3.719759	-3.051370	0.363704
20	1	0	-3.645117	-3.965332	0.047700
21	1	0	-5.252842	-2.262051	-0.121865
22	8	0	-5.963265	-1.723486	-0.551028
23	1	0	-6.368815	-1.198873	0.155633
24	1	0	-2.927729	-2.957397	2.068388
25	8	0	-2.394005	-2.855231	2.884469
26	1	0	-1.566032	-3.365680	2.721939
27	1	0	-1.905005	-1.240893	3.029138
28	8	0	-1.480499	-0.332100	3.037976
29	1	0	-1.865489	0.153691	3.782156
30	1	0	-5.119247	-0.789351	-1.751103
31	8	0	-4.468675	-0.319251	-2.338564
32	1	0	-4.847916	-0.307425	-3.229932
33	1	0	0.926230	-0.599304	1.397003
34	8	0	1.016331	-1.097416	2.256593
35	1	0	0.280801	-0.745766	2.811764
36	1	0	0.527606	-2.870642	2.312846
37	8	0	0.194724	-3.792773	2.392964
38	1	0	0.662411	-4.266078	1.679762

39	1	0	2.698092	-1.425989	2.030105
40	8	0	3.469709	-1.586561	1.424391
41	1	0	4.247167	-1.750511	1.978541
42	1	0	0.687778	-1.157738	-0.955686
43	8	0	0.498330	-2.040663	-1.372258
44	1	0	-0.482944	-2.042407	-1.472808
45	1	0	1.821867	-2.693038	-2.473403
46	8	0	2.721585	-3.055190	-2.653722
47	1	0	2.850670	-3.657713	-1.893126
48	1	0	1.362191	-3.263783	-0.315437
49	8	0	2.048486	-3.860496	0.066861
50	1	0	2.633082	-3.264360	0.573424
51	1	0	-2.740627	1.524095	-0.423363
52	8	0	-3.503929	1.995292	-0.860857
53	1	0	-3.923515	1.320886	-1.430371
54	1	0	-2.671709	3.223569	-1.748664
55	8	0	-1.999739	3.840008	-2.148158
56	1	0	-2.350517	4.735020	-2.034813
57	1	0	3.665398	-1.688044	-2.566921
58	8	0	4.068046	-0.773156	-2.443554
59	1	0	3.886551	-0.301751	-3.270802
60	1	0	5.586630	-0.043505	-1.417909
61	8	0	5.603335	0.420594	-0.556678
62	1	0	6.455618	0.870333	-0.472403
63	11	0	3.342831	0.004586	-0.301964
64	6	0	-0.472637	4.271781	2.186664
65	6	0	0.552685	4.180460	3.131350
66	1	0	-1.361550	3.434866	0.422380
67	1	0	2.159398	1.456930	1.890233
68	1	0	2.301095	3.078163	3.746712
69	1	0	-1.222980	5.053460	2.270633
70	1	0	0.607470	4.891484	3.951419
71	1	0	-0.231925	2.912876	-3.884154
72	1	0	1.503995	2.565037	-3.755393
73	1	0	0.458197	1.530627	-4.758123

SCF Done: E(RB+HF-LYP) = -1940.81514661   A.U. after   1 cycles

Convg =   0.4804D-08   -V/T = 2.0091

S**2   =   0.0000

Zero-point correction=   0.607715(a.u.)

Thermal correction to Energy=   0.660660

Thermal correction to Enthalpy=   0.661604

Thermal correction to Gibbs Free Energy=   0.518342

Sum of electronic and zero-point Energies=   -1940.207432

Sum of electronic and thermal Energies=   -1940.154487

Sum of electronic and thermal Enthalpies=   -1940.153543

Sum of electronic and thermal Free Energies= -1940.296805

(8-3) Anionic tetrahedral intermediate, Int1(am),
Ph-C(-O-)(OH)-NH-Et, Na+ and (H2O)16
tuka02bbna.rev.chk Stoichiometry C9H44NNaO18
Standard orientation:

Center Number	Atomic Number	Atomic Type	Coordinates (Angstroms) X	Y	Z
1	6	0	-1.791021	2.297395	-0.638961
2	6	0	-1.357299	0.823551	-0.780714
3	8	0	-0.874477	0.298467	0.382438
4	7	0	-0.342096	0.773146	-1.865077
5	6	0	-0.196663	-0.503224	-2.602118
6	6	0	-2.900504	2.796507	-1.334809
7	6	0	-1.041992	3.179545	0.150352
8	6	0	-1.402817	4.522968	0.258028
9	1	0	-0.561207	1.506253	-2.538813
10	6	0	0.816068	-0.353251	-3.736218
11	1	0	0.143684	-1.266721	-1.897497
12	1	0	-1.156063	-0.836871	-3.014085
13	8	0	-2.499129	0.034043	-1.229856
14	1	0	-3.214471	0.113322	-0.561898
15	1	0	-5.039611	-3.492255	0.795528
16	8	0	-5.007347	-2.873888	0.050888
17	1	0	-4.119822	-3.004205	-0.377563
18	1	0	1.340654	0.928848	-1.162992
19	8	0	2.978583	1.267308	1.888880
20	1	0	2.086444	1.375804	2.292953
21	1	0	3.196819	0.329869	2.075065
22	8	0	2.923098	-1.600259	2.197529
23	1	0	3.631136	-2.090476	2.642837
24	1	0	-4.932385	-1.200359	0.461623
25	8	0	-4.705302	-0.246432	0.641379
26	1	0	-5.508941	0.269450	0.478650
27	1	0	-2.038238	0.140211	1.685544
28	8	0	-2.598348	0.033691	2.504191
29	1	0	-3.503320	-0.059505	2.144168
30	1	0	3.086900	-1.728202	1.208362
31	8	0	3.287169	-1.699956	-0.442474
32	1	0	4.245976	-1.566959	-0.668721
33	1	0	5.760349	3.006359	1.601294
34	8	0	4.988140	2.989756	1.017279
35	1	0	4.303466	2.450523	1.473858
36	1	0	-0.482649	-1.254429	0.473914

37	8	0	-0.306716	-2.210368	0.773288
38	1	0	-1.126425	-2.660891	0.476324
39	1	0	6.346104	-1.244974	-1.741517
40	8	0	5.846979	-0.922710	-0.976678
41	1	0	5.634500	0.036776	-1.165689
42	1	0	2.158057	-3.176497	-1.014152
43	8	0	1.341865	-3.707223	-0.908738
44	1	0	0.822267	-3.187050	-0.251181
45	1	0	-2.425022	-1.836365	-1.183006
46	8	0	-2.485910	-2.788997	-0.930670
47	1	0	-1.980457	-3.296930	-1.614516
48	1	0	-0.820632	-5.032320	-2.835289
49	8	0	-0.733224	-4.093222	-2.617559
50	1	0	0.118260	-4.000625	-2.111419
51	1	0	-0.271812	1.162553	3.173701
52	8	0	0.398963	0.816532	2.563469
53	1	0	-0.056222	0.801195	1.654877
54	1	0	-1.539654	-1.721632	4.973594
55	8	0	-1.210980	-1.663613	4.066094
56	1	0	-1.860818	-1.101518	3.555133
57	1	0	5.117166	2.168687	-0.650529
58	8	0	4.985663	1.589048	-1.434779
59	1	0	4.014308	1.456671	-1.477653
60	1	0	2.855685	-0.843419	-0.706487
61	8	0	2.298863	0.824921	-0.878517
62	1	0	2.372907	1.118978	0.054409
63	11	0	0.629578	-1.568228	2.734333
64	6	0	-3.266680	4.139995	-1.222749
65	6	0	-2.520470	5.007128	-0.424531
66	1	0	-3.475866	2.126549	-1.966012
67	1	0	-0.168595	2.814991	0.680721
68	1	0	-0.808124	5.191762	0.874750
69	1	0	-4.134173	4.507957	-1.764869
70	1	0	-2.803330	6.052923	-0.338661
71	1	0	0.488161	0.402858	-4.461645
72	1	0	1.802152	-0.058820	-3.363328
73	1	0	0.917294	-1.303504	-4.270887

SCF Done: E(RB+HF-LYP) = -1940.83625089   A.U. after   1 cycles
Convg =   0.4394D-08   -V/T = 2.0090
S**2   =   0.0000
Zero-point correction=   0.611283 (a.u.)
Thermal correction to Energy=   0.662859
Thermal correction to Enthalpy=   0.663803
Thermal correction to Gibbs Free Energy=   0.525100
Sum of electronic and zero-point Energies=   -1940.224968

Sum of electronic and thermal Energies= -1940.173392
Sum of electronic and thermal Enthalpies= -1940.172448
Sum of electronic and thermal Free Energies= -1940.311151

(8-4) TS2(am) toward the zwitterion(i.e., Mulliken CT complex)
tuka02bbna.chk Stoichiometry C9H44NNaO18
Standard orientation:

Center Number	Atomic Number	Atomic Type	Coordinates (Angstroms) X	Y	Z
1	6	0	-1.551473	2.424483	-0.531199
2	6	0	-1.313474	0.907550	-0.654933
3	8	0	-0.910598	0.312711	0.462612
4	7	0	-0.208375	0.755758	-1.765704
5	6	0	-0.184352	-0.499500	-2.587075
6	6	0	-2.507446	3.073556	-1.324915
7	6	0	-0.769676	3.184858	0.347811
8	6	0	-0.949813	4.564911	0.443181
9	1	0	-0.281057	1.557712	-2.396633
10	6	0	0.887956	-0.407543	-3.668581
11	1	0	0.029495	-1.322909	-1.905696
12	1	0	-1.173143	-0.642183	-3.022472
13	8	0	-2.452059	0.264234	-1.246000
14	1	0	-3.204514	0.337994	-0.611699
15	1	0	-5.483005	-3.154979	0.369861
16	8	0	-5.263057	-2.479843	-0.288813
17	1	0	-4.366788	-2.717394	-0.645024
18	1	0	0.763353	0.812427	-1.270196
19	8	0	3.059707	1.030320	2.108027
20	1	0	2.199966	1.182437	2.549087
21	1	0	3.156742	0.049702	2.131364
22	8	0	2.817821	-1.788451	2.051135
23	1	0	3.559836	-2.314165	2.386023
24	1	0	-5.001909	-0.875531	0.265461
25	8	0	-4.672675	0.032173	0.516745
26	1	0	-5.418002	0.640248	0.398025
27	1	0	-2.102390	0.115640	1.779962
28	8	0	-2.673419	-0.030946	2.576732
29	1	0	-3.572978	-0.047326	2.196516
30	1	0	2.910733	-1.807996	0.984656
31	8	0	2.943421	-1.684056	-0.462276
32	1	0	3.883579	-1.695034	-0.749720
33	1	0	5.875683	2.493941	1.457430
34	8	0	5.036523	2.674591	1.009653
35	1	0	4.360301	2.142547	1.488186

36	1	0	-0.661745	-1.340847	0.558695
37	8	0	-0.572793	-2.294001	0.854012
38	1	0	-1.401600	-2.693217	0.521032
39	1	0	6.086842	-1.466537	-1.983401
40	8	0	5.652046	-1.148471	-1.179020
41	1	0	5.448630	-0.185417	-1.340005
42	1	0	1.908369	-2.972223	-0.992803
43	8	0	1.124574	-3.590290	-1.009707
44	1	0	0.582081	-3.246652	-0.268082
45	1	0	-2.527882	-1.688582	-1.258360
46	8	0	-2.675545	-2.639193	-1.060281
47	1	0	-2.146411	-3.141818	-1.734155
48	1	0	-0.940882	-4.712257	-3.111974
49	8	0	-0.875820	-3.811329	-2.763668
50	1	0	-0.037244	-3.778045	-2.214506
51	1	0	-0.297457	0.866091	3.372290
52	8	0	0.397187	0.599928	2.748994
53	1	0	-0.021209	0.681243	1.845274
54	1	0	-1.575838	-1.918780	4.972407
55	8	0	-1.256000	-1.840503	4.062959
56	1	0	-1.911944	-1.274587	3.575751
57	1	0	5.008107	1.950281	-0.746299
58	8	0	4.788791	1.408313	-1.535879
59	1	0	3.826935	1.211116	-1.414717
60	1	0	2.563296	-0.510519	-0.645636
61	8	0	2.229269	0.632403	-0.754891
62	1	0	2.367477	0.984589	0.145662
63	11	0	0.630167	-1.798902	2.732814
64	6	0	-2.691321	4.454371	-1.223705
65	6	0	-1.913951	5.202972	-0.339166
66	1	0	-3.109967	2.492871	-2.016642
67	1	0	-0.015211	2.698236	0.956173
68	1	0	-0.333468	5.140458	1.128149
69	1	0	-3.441594	4.943649	-1.839098
70	1	0	-2.054609	6.277668	-0.263034
71	1	0	0.681278	0.409679	-4.371633
72	1	0	1.880449	-0.255420	-3.236513
73	1	0	0.896789	-1.343166	-4.235911

SCF Done: E(RB+HF-LYP) = -1940.81873868 A.U. after 1 cycles

Convg = 0.3382D-08 -V/T = 2.0090

S**2 = 0.0000

Zero-point correction= 0.605882 (a.u.)

Thermal correction to Energy= 0.656922

Thermal correction to Enthalpy= 0.657866

Thermal correction to Gibbs Free Energy= 0.519940

Sum of electronic and zero-point Energies= -1940.212857
Sum of electronic and thermal Energies= -1940.161817
Sum of electronic and thermal Enthalpies= -1940.160873
Sum of electronic and thermal Free Energies= -1940.298799

(8-5) The zwitterion (i.e., Mulliken CT complex), Int2(am),
Ph-C(-O-)(OH)-N(H2+)-Et, NaOH and (H2O)15
tuka02ccna.for.chk Stoichiometry C9H44NNaO18
Standard orientation:

Center Number	Atomic Number	Atomic Type	Coordinates (Angstroms) X	Y	Z
1	6	0	0.172378	3.227014	-0.461961
2	6	0	-0.671871	1.982132	-0.759646
3	8	0	-0.856427	1.155739	0.254020
4	7	0	0.226528	1.189659	-1.903986
5	6	0	-0.489572	0.182972	-2.744242
6	6	0	-0.268232	4.504579	-0.824314
7	6	0	1.409428	3.087325	0.185285
8	6	0	2.188609	4.208165	0.470508
9	1	0	0.639382	1.902951	-2.510499
10	6	0	0.402615	-0.329204	-3.872008
11	1	0	-0.781340	-0.635931	-2.085192
12	1	0	-1.388954	0.667878	-3.127739
13	8	0	-1.841312	2.330378	-1.447723
14	1	0	-2.606889	1.915643	-0.993056
15	1	0	-6.367238	-0.793157	0.678381
16	8	0	-5.907482	-0.600627	-0.152117
17	1	0	-5.330145	-1.379769	-0.338729
18	1	0	1.013468	0.702137	-1.411534
19	8	0	2.820948	-0.506062	1.897592
20	1	0	2.217680	0.107034	2.360062
21	1	0	2.361495	-1.432806	2.017639
22	8	0	1.450981	-2.673889	2.036052
23	1	0	1.840303	-3.372411	2.581357
24	1	0	-4.953358	0.854356	-0.209689
25	8	0	-4.374046	1.661298	-0.178090
26	1	0	-4.929340	2.401835	-0.466456
27	1	0	-2.108523	1.434263	1.533559
28	8	0	-2.830405	1.418081	2.206997
29	1	0	-3.619714	1.580721	1.654395
30	1	0	1.742508	-3.055862	0.476528
31	8	0	1.873639	-3.109183	-0.533149
32	1	0	2.777301	-3.469862	-0.698085
33	1	0	6.018880	-0.868368	2.073861

34	8	0	5.520398	-0.245923	1.524661
35	1	0	4.572878	-0.337523	1.799309
36	1	0	-1.426388	-0.440571	0.170506
37	8	0	-1.607502	-1.405785	0.339109
38	1	0	-2.534108	-1.583991	0.076268
39	1	0	4.862587	-4.122520	-1.830384
40	8	0	4.554694	-3.675943	-1.028571
41	1	0	4.860229	-2.729848	-1.101195
42	1	0	0.291141	-3.525493	-1.244832
43	8	0	-0.670682	-3.377061	-1.423584
44	1	0	-0.893891	-2.646780	-0.804286
45	1	0	-3.891541	-2.223011	-1.846927
46	8	0	-3.997877	-2.411440	-0.900858
47	1	0	-3.723252	-3.370554	-0.816814
48	1	0	-2.784193	-5.474718	-0.263751
49	8	0	-2.860201	-4.829661	-0.981552
50	1	0	-1.940250	-4.473656	-1.152512
51	1	0	-0.047622	1.219579	3.255021
52	8	0	0.349103	0.638616	2.588401
53	1	0	0.030180	0.981569	1.708428
54	1	0	-2.668841	-0.903165	4.415783
55	8	0	-2.417794	-0.979521	3.484323
56	1	0	-2.677081	-0.120057	3.056423
57	1	0	5.412331	-0.722949	-0.264372
58	8	0	5.105935	-1.012488	-1.155744
59	1	0	4.186229	-0.674962	-1.209208
60	1	0	2.073056	-1.383406	-0.852364
61	8	0	2.294767	-0.412054	-0.801266
62	1	0	2.486218	-0.308637	0.169405
63	11	0	-0.469578	-1.632506	2.371546
64	6	0	0.511261	5.626385	-0.531572
65	6	0	1.739172	5.481996	0.114349
66	1	0	-1.223713	4.612156	-1.324802
67	1	0	1.766982	2.102905	0.471714
68	1	0	3.145066	4.083543	0.970264
69	1	0	0.155335	6.614670	-0.810401
70	1	0	2.344993	6.355759	0.338420
71	1	0	0.685238	0.477597	-4.559969
72	1	0	1.314669	-0.791623	-3.483048
73	1	0	-0.138720	-1.086983	-4.446996

SCF Done: E(RB+HF-LYP) = -1940.82661398   A.U. after   1 cycles

Convg =   0.2289D-08   -V/T = 2.0091

S**2   =   0.0000

Zero-point correction=   0.610399 (a.u.)

Thermal correction to Energy=   0.662633

Thermal correction to Enthalpy= 0.663577
Thermal correction to Gibbs Free Energy= 0.521463
Sum of electronic and zero-point Energies= -1940.216215
Sum of electronic and thermal Energies= -1940.163981
Sum of electronic and thermal Enthalpies= -1940.163037
Sum of electronic and thermal Free Energies= -1940.305151

(8-6) TS3(am), C(2)----N(7) scission TS
tuka02ccna.chk Stoichiometry C9H44NNaO18
Standard orientation:

Center Number	Atomic Number	Atomic Type	Coordinates (Angstroms) X	Y	Z
1	6	0	0.341215	3.340841	-0.237571
2	6	0	-0.688319	2.253572	-0.426717
3	8	0	-0.834394	1.348779	0.474597
4	7	0	0.213532	1.326514	-1.902647
5	6	0	-0.675002	0.578657	-2.812584
6	6	0	0.095049	4.651156	-0.666318
7	6	0	1.563342	3.035026	0.378351
8	6	0	2.523606	4.027441	0.567184
9	1	0	0.727669	2.041673	-2.418898
10	6	0	0.063411	-0.052753	-3.994641
11	1	0	-1.163135	-0.198727	-2.217423
12	1	0	-1.447475	1.272454	-3.159770
13	8	0	-1.812830	2.697591	-1.060183
14	1	0	-2.591914	2.177503	-0.754611
15	1	0	-6.612371	-0.582273	0.091119
16	8	0	-5.918137	-0.329761	-0.535132
17	1	0	-5.329513	-1.117564	-0.638997
18	1	0	0.911386	0.692996	-1.471032
19	8	0	2.787862	-0.784736	1.873488
20	1	0	2.235732	-0.175358	2.397406
21	1	0	2.301984	-1.700737	1.965780
22	8	0	1.355166	-2.913995	1.954594
23	1	0	1.716799	-3.634609	2.489832
24	1	0	-4.946897	1.063625	-0.228862
25	8	0	-4.338876	1.828047	-0.038256
26	1	0	-4.855911	2.630664	-0.207986
27	1	0	-2.163119	1.397518	1.777133
28	8	0	-2.928581	1.275172	2.380968
29	1	0	-3.680957	1.477219	1.789942
30	1	0	1.640567	-3.278158	0.375265
31	8	0	1.752497	-3.308148	-0.634308
32	1	0	2.655137	-3.654462	-0.831672

33	1	0	5.972845	-1.249573	1.871487
34	8	0	5.477768	-0.563032	1.401096
35	1	0	4.537747	-0.653255	1.700427
36	1	0	-1.386021	-0.297705	0.183041
37	8	0	-1.563635	-1.268074	0.246991
38	1	0	-2.496379	-1.415059	-0.016428
39	1	0	4.684218	-4.257200	-2.063305
40	8	0	4.420661	-3.837946	-1.231450
41	1	0	4.714502	-2.886862	-1.294162
42	1	0	0.161091	-3.556124	-1.365646
43	8	0	-0.786848	-3.326532	-1.537396
44	1	0	-0.920618	-2.540301	-0.966175
45	1	0	-3.774775	-2.054748	-1.949700
46	8	0	-3.970777	-2.175939	-1.006448
47	1	0	-3.770317	-3.144901	-0.847117
48	1	0	-2.918803	-5.226470	-0.115371
49	8	0	-3.000182	-4.655883	-0.893165
50	1	0	-2.076017	-4.346021	-1.126488
51	1	0	0.021608	0.944551	3.474618
52	8	0	0.367966	0.440788	2.722626
53	1	0	0.068812	0.924331	1.910426
54	1	0	-2.785846	-1.299391	4.308511
55	8	0	-2.471981	-1.239895	3.395125
56	1	0	-2.752146	-0.347103	3.061738
57	1	0	5.295443	-0.922445	-0.416471
58	8	0	4.957785	-1.170857	-1.308402
59	1	0	4.034720	-0.833913	-1.312746
60	1	0	1.950829	-1.545085	-0.914562
61	8	0	2.182980	-0.582171	-0.832282
62	1	0	2.391653	-0.511262	0.133818
63	11	0	-0.505799	-1.784496	2.275435
64	6	0	1.057340	5.643636	-0.470236
65	6	0	2.271211	5.335278	0.145140
66	1	0	-0.850910	4.887995	-1.140103
67	1	0	1.767719	2.019103	0.700970
68	1	0	3.468653	3.776716	1.040271
69	1	0	0.855425	6.659590	-0.798668
70	1	0	3.019820	6.108726	0.293233
71	1	0	0.557332	0.711276	-4.607535
72	1	0	0.820886	-0.764727	-3.652830
73	1	0	-0.643057	-0.592438	-4.635091

SCF Done: E(RB+HF-LYP) = -1940.82510814 A.U. after 1 cycles

Convg = 0.1902D-08 -V/T = 2.0091

S**2 = 0.0000

Zero-point correction= 0.608575 (a.u.)

Thermal correction to Energy= 0.661088
Thermal correction to Enthalpy= 0.662033
Thermal correction to Gibbs Free Energy= 0.518382
Sum of electronic and zero-point Energies= -1940.216533
Sum of electronic and thermal Energies= -1940.164020
Sum of electronic and thermal Enthalpies= -1940.163076
Sum of electronic and thermal Free Energies= -1940.306726

(8-7) Ph-COOH, Et-NH2 and NaOH(H2O)15, Int3(am)
tuka02ccna.rev.chk Stoichiometry C9H44NNaO18
Standard orientation:

Center Number	Atomic Number	Atomic Type	Coordinates (Angstroms) X	Y	Z
1	6	0	2.826328	2.482038	-0.369214
2	6	0	3.336563	1.174327	-0.848041
3	8	0	2.913825	0.618036	-1.872172
4	7	0	-0.568908	1.573708	1.698476
5	6	0	-0.821301	1.686200	3.150100
6	6	0	3.254453	3.020308	0.854745
7	6	0	1.889640	3.179165	-1.147857
8	6	0	1.381860	4.398028	-0.704570
9	1	0	-0.064728	2.395455	1.364235
10	6	0	-1.620924	2.924516	3.561571
11	1	0	-1.352463	0.777553	3.454216
12	1	0	0.148662	1.673892	3.663873
13	8	0	4.286898	0.648805	-0.088564
14	1	0	4.553914	-0.273882	-0.379679
15	1	0	2.584163	-3.556360	-0.136786
16	8	0	3.310273	-3.504204	0.522702
17	1	0	2.894071	-3.177909	1.355208
18	1	0	-1.467918	1.545185	1.195649
19	8	0	-2.589387	0.345022	-2.393845
20	1	0	-1.687962	0.566376	-2.683566
21	1	0	-2.552685	-0.671875	-2.158665
22	8	0	-2.338387	-2.073810	-1.606828
23	1	0	-2.756896	-2.743253	-2.165639
24	1	0	4.440323	-2.512416	-0.108547
25	8	0	5.049814	-1.889061	-0.622994
26	1	0	5.960286	-2.109529	-0.372945
27	1	0	3.270286	-1.036855	-2.686504
28	8	0	3.418013	-1.977961	-2.920289
29	1	0	4.215159	-2.193790	-2.395965
30	1	0	-3.264037	-1.913585	-0.241429
31	8	0	-3.694815	-1.646133	0.635079

32	1	0	-4.672149	-1.641651	0.498499
33	1	0	-5.457405	0.748478	-3.778029
34	8	0	-4.998507	1.394572	-3.221606
35	1	0	-4.104393	1.010154	-3.043475
36	1	0	0.106882	0.080105	1.253595
37	8	0	0.334309	-0.874754	0.969509
38	1	0	1.012746	-1.219161	1.586406
39	1	0	-7.063391	-1.217566	0.796981
40	8	0	-6.390808	-1.184193	0.101413
41	1	0	-6.297770	-0.221577	-0.145444
42	1	0	-2.607442	-2.070444	1.977590
43	8	0	-1.773482	-2.071578	2.510667
44	1	0	-1.122804	-1.613646	1.936289
45	1	0	2.476048	-2.130222	3.495460
46	8	0	1.983669	-2.447574	2.723461
47	1	0	1.245372	-3.025739	3.083973
48	1	0	-0.273896	-4.734804	3.345353
49	8	0	-0.121189	-3.819907	3.622674
50	1	0	-0.878701	-3.277096	3.253780
51	1	0	0.462028	-0.410160	-3.621708
52	8	0	0.348220	-0.082734	-2.715981
53	1	0	1.205746	0.329549	-2.471729
54	1	0	1.182333	-4.103277	-2.081065
55	8	0	1.453818	-3.291236	-1.625903
56	1	0	2.145582	-2.857015	-2.204976
57	1	0	-5.715070	1.486136	-1.488885
58	8	0	-5.871942	1.412354	-0.519797
59	1	0	-4.970543	1.480450	-0.130919
60	1	0	-3.337763	0.128983	0.536035
61	8	0	-3.148776	1.061604	0.257050
62	1	0	-2.892415	0.950794	-0.690105
63	11	0	-0.250299	-1.731991	-1.066629
64	6	0	2.743856	4.240296	1.293916
65	6	0	1.807294	4.928648	0.517084
66	1	0	3.978090	2.478393	1.452784
67	1	0	1.568113	2.764743	-2.097224
68	1	0	0.656757	4.933656	-1.309808
69	1	0	3.076386	4.655160	2.240948
70	1	0	1.410671	5.879667	0.861585
71	1	0	-1.091903	3.846489	3.287487
72	1	0	-2.598927	2.936021	3.067893
73	1	0	-1.785690	2.941381	4.645814

SCF Done: E(RB+HF-LYP) = -1940.86175587   A.U. after   1 cycles

Convg =   0.3267D-08   -V/T = 2.0091

S**2   =   0.0000

Zero-point correction= 0.609265 (a.u.)
Thermal correction to Energy= 0.662209
Thermal correction to Enthalpy= 0.663154
Thermal correction to Gibbs Free Energy= 0.516907
Sum of electronic and zero-point Energies= -1940.252491
Sum of electronic and thermal Energies= -1940.199546
Sum of electronic and thermal Enthalpies= -1940.198602
Sum of electronic and thermal Free Energies= -1940.344849

(8-8) TS4(am) leading to the product
tuka02d1na.chk Stoichiometry C9H44NNaO18
standard orientation:

Center Number	Atomic Number	Atomic Type	Coordinates (Angstroms) X	Y	Z
1	6	0	-3.860491	1.431750	-0.164245
2	6	0	-3.029012	0.591816	-1.086420
3	8	0	-3.618275	-0.102505	-1.970087
4	7	0	0.488601	-1.994185	1.951954
5	6	0	1.085603	-2.770489	3.054300
6	6	0	-5.140265	1.851105	-0.553490
7	6	0	-5.905779	2.646213	0.298036
8	6	0	-5.403400	3.013954	1.549022
9	1	0	1.016195	-2.238240	4.016295
10	1	0	2.154952	-2.893270	2.838703
11	6	0	0.423456	-4.140462	3.184241
12	8	0	-1.763980	0.623440	-0.930314
13	1	0	0.939378	1.690490	-1.394268
14	1	0	-0.971497	-0.327957	-1.698092
15	8	0	-0.313844	-0.950633	-2.249761
16	1	0	-0.856245	-1.196169	-3.054691
17	8	0	-2.508375	-1.483868	2.504951
18	1	0	-3.103060	-2.071697	1.978168
19	1	0	-2.970295	-1.341844	3.343750
20	8	0	0.586076	2.177442	-0.586725
21	1	0	1.389765	1.264163	0.800637
22	1	0	-0.362256	1.925555	-0.550525
23	8	0	1.503318	0.619762	-2.546073
24	1	0	0.581205	-0.241184	-2.507945
25	1	0	1.647983	0.950968	-3.444386
26	1	0	0.436637	-2.445168	-1.158654
27	8	0	0.831471	-3.089504	-0.539272
28	1	0	0.665558	-2.710322	0.391477
29	8	0	1.978088	0.666988	1.320205
30	1	0	0.962862	-1.090744	1.867214

31	1	0	2.479509	1.238343	1.947583
32	1	0	-2.849481	-0.852959	-3.333691
33	8	0	-2.254590	-1.265015	-4.019875
34	1	0	-2.637192	-2.130410	-4.227290
35	8	0	3.524987	2.360446	2.916114
36	1	0	3.062030	2.931319	3.546754
37	1	0	3.928800	2.958229	2.244851
38	8	0	4.462658	3.849413	0.756689
39	1	0	3.607051	4.093891	0.303840
40	1	0	4.952110	4.677271	0.875814
41	1	0	-4.754232	-1.344454	-1.495584
42	8	0	-5.311936	-2.101224	-1.183973
43	1	0	-6.201633	-1.740871	-1.057356
44	1	0	-4.605921	-2.815337	0.273750
45	8	0	-4.180047	-3.208742	1.076010
46	1	0	-3.715539	-3.999139	0.763039
47	1	0	2.742720	0.189284	-1.624507
48	8	0	3.430271	0.003697	-0.908822
49	1	0	2.957872	0.192409	-0.054693
50	1	0	1.427836	3.685178	-0.404805
51	8	0	2.044012	4.461704	-0.319635
52	1	0	1.995398	4.925377	-1.168660
53	11	0	4.655898	-1.844704	-0.812225
54	8	0	6.604719	-0.770917	-0.470758
55	1	0	7.558673	-0.878232	-0.355369
56	1	0	6.422594	0.209837	-0.526289
57	8	0	3.371374	-3.632474	-0.932766
58	1	0	2.384571	-3.428887	-0.800745
59	1	0	3.429338	-4.589631	-1.058174
60	8	0	5.586438	1.685418	-0.856063
61	1	0	4.717042	1.224927	-0.964455
62	1	0	5.397012	2.426810	-0.246620
63	6	0	-3.361391	1.800481	1.092054
64	6	0	-4.134998	2.584229	1.947000
65	1	0	-5.515522	1.563318	-1.531067
66	1	0	-6.891752	2.981313	-0.013652
67	1	0	-6.001033	3.631689	2.214597
68	1	0	-2.385264	1.436990	1.395829
69	1	0	-3.749632	2.858711	2.925320
70	1	0	-0.644970	-4.033873	3.407160
71	1	0	0.517267	-4.707230	2.251732
72	1	0	0.881950	-4.721408	3.993559
73	1	0	-0.497130	-1.805011	2.160492

SCF Done: E(RB3LYP) = -1940.84877758 A.U. after 13 cycles
Convg = 0.3257D-08 -V/T = 2.0090

Zero-point correction= 0.600769 (a.u.)
Thermal correction to Energy= 0.655273
Thermal correction to Enthalpy= 0.656217
Thermal correction to Gibbs Free Energy= 0.501791
Sum of electronic and zero-point Energies= -1940.248009
Sum of electronic and thermal Energies= -1940.193504
Sum of electronic and thermal Enthalpies= -1940.192560
Sum of electronic and thermal Free Energies= -1940.346986

(8-9) Product, Ph-COONa + Et-NH2 + (H2O)16
tuka02d1na.for.chk Stoichiometry C9H44NNaO18
Standard orientation:

Center Number	Atomic Number	Atomic Type	Coordinates (Angstroms) X	Y	Z
1	6	0	-3.777219	1.546187	0.016509
2	6	0	-3.033283	0.803052	-1.064867
3	8	0	-3.706599	0.077296	-1.855905
4	7	0	0.319939	-1.423436	2.070731
5	6	0	0.972902	-2.010179	3.256207
6	6	0	-5.171959	1.672506	-0.048555
7	6	0	-5.865958	2.346251	0.955239
8	6	0	-5.175151	2.887256	2.042635
9	1	0	0.953480	-1.320388	4.114519
10	1	0	2.030078	-2.179195	3.010658
11	6	0	0.313839	-3.330177	3.651094
12	8	0	-1.766550	0.927134	-1.113166
13	1	0	0.891791	1.767196	-1.919826
14	1	0	-0.966141	-0.398911	-1.804425
15	8	0	-0.446723	-1.125641	-2.260586
16	1	0	-1.105339	-1.480475	-2.916379
17	8	0	-2.664059	-1.358445	2.444086
18	1	0	-2.750497	-2.092507	1.784443
19	1	0	-3.178626	-0.617329	2.090336
20	8	0	0.567353	2.293513	-1.153266
21	1	0	1.377778	1.494793	0.286168
22	1	0	-0.382048	2.021500	-1.055862
23	8	0	1.494893	0.429711	-3.074469
24	1	0	0.752220	-0.237124	-2.908896
25	1	0	1.566710	0.553896	-4.032768
26	1	0	-0.023940	-2.323033	-0.922338
27	8	0	0.159919	-2.941456	-0.175330
28	1	0	0.233191	-2.362179	0.669072
29	8	0	1.949852	0.922329	0.849543
30	1	0	0.776643	-0.546719	1.808243

31	1	0	2.465227	1.517848	1.445185
32	1	0	-3.023530	-0.857199	-3.306890
33	8	0	-2.623424	-1.608481	-3.809009
34	1	0	-3.131847	-2.372274	-3.483082
35	8	0	3.527759	2.603698	2.394495
36	1	0	3.087123	3.260909	2.953018
37	1	0	4.022886	3.105645	1.707352
38	8	0	4.676723	3.714374	0.121062
39	1	0	3.847364	3.988587	-0.354537
40	1	0	5.269532	4.480692	0.100004
41	1	0	-4.261787	-1.577386	-1.548182
42	8	0	-4.334724	-2.563820	-1.578223
43	1	0	-5.265043	-2.779000	-1.415501
44	1	0	-3.182407	-3.192787	-0.229994
45	8	0	-2.619295	-3.390722	0.548263
46	1	0	-1.690900	-3.386118	0.229621
47	1	0	2.796394	-0.072286	-1.946586
48	8	0	3.354352	-0.228309	-1.148910
49	1	0	2.855899	0.204166	-0.397864
50	1	0	1.559838	3.738914	-1.011263
51	8	0	2.258768	4.437690	-0.936862
52	1	0	2.101868	5.055307	-1.665759
53	11	0	4.062780	-2.185965	-0.307848
54	8	0	5.986802	-1.279728	0.423242
55	1	0	6.778262	-1.472058	0.944670
56	1	0	6.060674	-0.333885	0.117054
57	8	0	2.628172	-3.864122	-0.377350
58	1	0	1.658061	-3.574699	-0.336042
59	1	0	2.622624	-4.831343	-0.360966
60	8	0	5.737701	1.125525	-0.785589
61	1	0	4.877754	0.778684	-1.118223
62	1	0	5.522854	2.007808	-0.421872
63	6	0	-3.089455	2.097960	1.107143
64	6	0	-3.786266	2.759089	2.119062
65	1	0	-5.694618	1.242277	-0.896428
66	1	0	-6.946315	2.446729	0.892793
67	1	0	-5.717784	3.404897	2.829337
68	1	0	-2.010601	1.991961	1.156384
69	1	0	-3.246448	3.173117	2.966434
70	1	0	-0.744985	-3.178819	3.888439
71	1	0	0.373719	-4.058515	2.834453
72	1	0	0.803505	-3.758738	4.533336
73	1	0	-0.664818	-1.218325	2.280361

SCF Done: E(RB+HF-LYP) = -1940.86925259 A.U. after 1 cycles

Convg = 0.2598D-08 -V/T = 2.0090

S**2 = 0.0000
Zero-point correction= 0.608875 (a.u.)
Thermal correction to Energy= 0.663116
Thermal correction to Enthalpy= 0.664060
Thermal correction to Gibbs Free Energy= 0.513287
Sum of electronic and zero-point Energies= -1940.260378
Sum of electronic and thermal Energies= -1940.206137
Sum of electronic and thermal Enthalpies= -1940.205192
Sum of electronic and thermal Free Energies= -1940.355966

==

[9] B3LYP/6-311+G(d,p) geometries of the reactant-like complex (precursor), TS1(am) and T2(am)

(9-1) precursor composed of Ph-C(=O)-NH-Et + HO-(H2O)16

Stoichiometry C9H44NO18(1-)

tuka02aaA.for.log Standard orientation:

Center Number	Atomic Number	Atomic Type	Coordinates (Angstroms) X	Y	Z
1	6	0	-0.499203	2.783375	-0.571371
2	6	0	-0.563601	1.822573	0.581891
3	8	0	-1.436696	0.923726	0.591115
4	7	0	0.288228	2.006528	1.596346
5	6	0	0.347439	1.114923	2.757416
6	6	0	-1.659104	2.960325	-1.334224
7	6	0	-1.670162	3.844240	-2.408862
8	6	0	0.659105	3.491095	-0.913722
9	1	0	1.075540	2.641784	1.486252
10	6	0	0.930000	1.835791	3.969091
11	1	0	-0.663937	0.767788	2.965835
12	1	0	0.945263	0.234130	2.502918
13	8	0	1.993131	0.066980	-0.704809
14	1	0	1.509488	-0.125856	-1.524185
15	1	0	1.797081	-0.670779	-0.058179
16	8	0	1.616962	-2.027926	0.916532
17	1	0	2.139710	-1.995038	1.723520
18	1	0	2.382696	-2.686338	-0.111899
19	8	0	2.970959	-3.041976	-0.922012
20	1	0	3.190156	-2.228318	-1.393267
21	1	0	4.448113	-3.356543	-0.139911
22	8	0	5.314639	-3.381441	0.351728
23	1	0	5.176650	-3.984340	1.087597
24	1	0	1.564848	-3.487153	-2.147377
25	8	0	0.906618	-3.369448	-2.860526
26	1	0	0.033077	-3.637931	-2.499690
27	1	0	0.698553	-1.749733	-3.085895
28	8	0	0.525596	-0.761310	-3.109130
29	1	0	0.766548	-0.460814	-3.990038
30	1	0	5.827765	-1.699501	0.670593
31	8	0	6.145613	-0.771523	0.747579
32	1	0	6.919548	-0.732537	0.178390
33	1	0	-1.841434	-0.103503	-1.078641
34	8	0	-2.033456	-0.575727	-1.907067
35	1	0	-1.208350	-0.516302	-2.425955
36	1	0	-1.901697	-2.617156	-2.026373
37	8	0	-1.723681	-3.571888	-2.062985

38	1	0	-2.133766	-3.895922	-1.239788
39	1	0	-3.905841	-1.226164	-1.516707
40	8	0	-4.610180	-1.671153	-1.020936
41	1	0	-5.081313	-0.970691	-0.531714
42	1	0	-1.063967	-0.963393	1.128015
43	8	0	-0.927002	-1.876642	1.424373
44	1	0	0.060017	-2.042088	1.256273
45	1	0	-2.113373	-2.095439	2.817379
46	8	0	-3.006608	-2.128746	3.219791
47	1	0	-3.454406	-2.769657	2.647780
48	1	0	-2.275817	-2.964499	0.725137
49	8	0	-3.049473	-3.512371	0.482712
50	1	0	-3.671803	-2.903675	0.034060
51	1	0	3.512323	0.848359	-0.348028
52	8	0	4.294916	1.315487	0.026204
53	1	0	4.900148	0.616294	0.335252
54	1	0	3.589365	2.459625	1.047183
55	8	0	3.050855	3.134302	1.542943
56	1	0	3.599937	3.920325	1.600558
57	1	0	-3.546792	-0.427349	2.755902
58	8	0	-3.719761	0.461568	2.373986
59	1	0	-2.945266	0.649910	1.817980
60	1	0	-5.094614	0.387309	1.319521
61	8	0	-5.806707	0.293799	0.639446
62	1	0	-6.065554	1.189219	0.406994
63	6	0	0.647266	4.367100	-1.996397
64	6	0	-0.515789	4.550835	-2.743058
65	1	0	-2.544843	2.393836	-1.078168
66	1	0	-2.576739	3.973660	-2.989441
67	1	0	1.577457	3.349942	-0.357645
68	1	0	1.554275	4.898622	-2.262484
69	1	0	-0.519599	5.233576	-3.586054
70	1	0	1.934352	2.208652	3.757056
71	1	0	0.300028	2.680310	4.262979
72	1	0	0.987069	1.144435	4.813701

SCF Done: E(RB+HF-LYP) = -1779.27977846   A.U. after   2 cycles

Convg =   0.2767D-08   -V/T = 2.0045

Zero-point correction=   0.603199 (a.u.)

Thermal correction to Energy=   0.656616

Thermal correction to Enthalpy=   0.657561

Thermal correction to Gibbs Free Energy=   0.509767

Sum of electronic and zero-point Energies=   -1778.676579

Sum of electronic and thermal Energies=   -1778.623162

Sum of electronic and thermal Enthalpies=   -1778.622218

Sum of electronic and thermal Free Energies=   -1778.770011

(9-2) TS1(am), OH- addition step

Stoichiometry C9H44NO18(1-)

tuka02aaA.chk Stoichiometry C9H44NO18(1-)

Standard orientation:

Center Number	Atomic Number	Atomic Type	Coordinates (Angstroms) X	Y	Z
1	6	0	-0.546702	2.408401	-0.831962
2	6	0	-0.209949	1.375026	0.257308
3	8	0	-1.125726	0.463377	0.478868
4	7	0	0.301276	2.043319	1.396386
5	6	0	0.634211	1.289941	2.600510
6	6	0	-1.881039	2.592709	-1.197450
7	6	0	-2.233385	3.546036	-2.151956
8	6	0	0.431856	3.201553	-1.440976
9	1	0	1.010526	2.734234	1.173419
10	6	0	0.858024	2.240735	3.775115
11	1	0	-0.192119	0.614631	2.818619
12	1	0	1.531606	0.666739	2.461492
13	8	0	1.121639	0.568736	-0.417193
14	1	0	0.860888	0.294566	-1.315446
15	1	0	1.607333	-0.819542	0.319802
16	8	0	1.998575	-1.670110	0.688231
17	1	0	2.728946	-1.390079	1.264102
18	1	0	2.793948	-2.337676	-0.641024
19	8	0	3.365370	-2.673046	-1.387501
20	1	0	3.513611	-1.901171	-1.946388
21	1	0	4.852117	-3.010882	-0.475095
22	8	0	5.587670	-3.031905	0.181550
23	1	0	5.598775	-3.928715	0.528516
24	1	0	1.757882	-3.264009	-2.567282
25	8	0	1.032144	-3.086498	-3.183682
26	1	0	0.210423	-3.437763	-2.757331
27	1	0	0.683750	-1.409044	-3.216648
28	8	0	0.423586	-0.454962	-3.119057
29	1	0	0.665185	-0.013971	-3.938400
30	1	0	5.107086	-1.635362	1.206116
31	8	0	4.622429	-0.874253	1.596727
32	1	0	5.065706	-0.643693	2.418170
33	1	0	-1.778238	-0.392603	-0.923320
34	8	0	-2.097343	-0.851482	-1.740641
35	1	0	-1.475221	-0.556005	-2.424074
36	1	0	-1.673092	-2.688066	-1.979117
37	8	0	-1.374879	-3.612286	-2.086363

38	1	0	-1.663946	-4.019150	-1.244492
39	1	0	-3.804403	-1.690584	-1.155424
40	8	0	-4.423265	-2.200765	-0.608196
41	1	0	-4.854338	-1.542844	-0.029421
42	1	0	-0.837649	-1.074203	1.177763
43	8	0	-0.708531	-1.964996	1.601204
44	1	0	0.237441	-2.144265	1.470175
45	1	0	-1.647388	-1.876565	3.120108
46	8	0	-2.330903	-1.686780	3.799909
47	1	0	-2.996484	-2.369190	3.665412
48	1	0	-1.878575	-3.319194	0.883835
49	8	0	-2.480781	-3.968026	0.476440
50	1	0	-3.255652	-3.438323	0.196266
51	1	0	2.796548	1.095316	-0.306049
52	8	0	3.738024	1.356855	-0.155809
53	1	0	4.116631	0.651618	0.392087
54	1	0	3.511585	2.861746	0.771118
55	8	0	3.215843	3.642466	1.291451
56	1	0	3.454365	4.407839	0.761732
57	1	0	-2.953921	-0.053862	2.955328
58	8	0	-3.219356	0.640946	2.324632
59	1	0	-2.474904	0.696379	1.684448
60	1	0	-4.666479	0.137711	1.572729
61	8	0	-5.476559	-0.209855	1.116665
62	1	0	-5.923894	0.562893	0.761917
63	6	0	0.082329	4.155879	-2.394399
64	6	0	-1.253637	4.332345	-2.754799
65	1	0	-2.637856	1.975520	-0.731725
66	1	0	-3.275703	3.671106	-2.426061
67	1	0	1.473342	3.062727	-1.179526
68	1	0	0.855204	4.758811	-2.860362
69	1	0	-1.526431	5.072735	-3.499576
70	1	0	1.675998	2.934771	3.562019
71	1	0	-0.046519	2.821763	3.971965
72	1	0	1.111236	1.677335	4.678692

SCF Done: E(RB+HF-LYP) = -1779.24427796 A.U. after 1 cycles
Convg = 0.5611D-08 -V/T = 2.0045
Zero-point correction= 0.604806 (a.u.)
Thermal correction to Energy= 0.656899
Thermal correction to Enthalpy= 0.657843
Thermal correction to Gibbs Free Energy= 0.515283
Sum of electronic and zero-point Energies= -1778.639472
Sum of electronic and thermal Energies= -1778.587379
Sum of electronic and thermal Enthalpies= -1778.586435
Sum of electronic and thermal Free Energies= -1778.728995

(9-3) TS2(am) toward the Mulliken CT complex
stoichiometry C9H44NO18(1-)

tuka02bzA.chk Standard orientation:

Center Number	Atomic Number	Atomic Type	Coordinates (Angstroms) X	Y	Z
1	6	0	-1.443656	0.990498	-0.999210
2	6	0	-1.791453	-0.411947	-0.447736
3	8	0	-2.584070	-0.398194	0.624447
4	7	0	-0.470538	-1.114289	-0.103050
5	6	0	-0.669353	-2.502998	0.399191
6	6	0	-1.828564	2.139827	-0.307919
7	6	0	-1.540028	3.405764	-0.822096
8	6	0	-0.852669	3.534761	-2.028604
9	1	0	0.145875	-1.138798	-0.920861
10	1	0	-1.208909	-2.422750	1.342605
11	1	0	-1.309554	-3.031360	-0.308311
12	6	0	0.651852	-3.238935	0.591269
13	8	0	-2.312465	-1.205766	-1.528189
14	1	0	-3.218896	-0.894619	-1.699995
15	1	0	-5.117214	-2.941092	-0.067859
16	8	0	-5.469911	-3.203265	-0.959763
17	1	0	-4.811419	-3.806810	-1.317445
18	1	0	0.108696	-0.455394	0.719120
19	8	0	0.814315	0.257266	1.589525
20	1	0	0.250461	0.422462	2.356999
21	1	0	2.326631	-0.367400	1.560439
22	8	0	3.213875	-0.622913	1.168182
23	1	0	3.601832	-1.417014	1.576851
24	1	0	-5.390228	-1.599809	-1.704926
25	8	0	-5.173813	-0.672625	-1.963034
26	1	0	-5.525008	-0.545474	-2.848888
27	1	0	-4.157340	0.355077	0.641001
28	8	0	-5.103201	0.605779	0.783094
29	1	0	-5.479731	0.530321	-0.103874
30	1	0	2.691415	-1.032795	-0.553052
31	8	0	2.442011	-1.151552	-1.493693
32	1	0	2.614915	-0.260466	-1.862471
33	1	0	4.372986	0.542163	0.665524
34	8	0	5.053236	1.138073	0.264900
35	1	0	4.939936	1.984273	0.739591
36	1	0	-3.658827	-1.723308	1.053009
37	8	0	-4.468830	-2.214279	1.346311
38	1	0	-5.071408	-1.475843	1.526009

39	1	0	6.285004	-0.077804	0.014015
40	8	0	6.744118	-0.939674	-0.126338
41	1	0	7.650624	-0.726389	-0.363065
42	1	0	3.993645	-2.310323	-1.635425
43	8	0	4.798664	-2.814112	-1.405079
44	1	0	5.486305	-2.142847	-1.273310
45	1	0	-2.230074	-0.185115	3.665892
46	8	0	-1.929972	0.503325	3.064547
47	1	0	-2.120134	0.150431	2.152881
48	1	0	-3.361699	1.949393	3.028162
49	8	0	-4.121165	2.524217	2.838823
50	1	0	-4.627122	2.032616	2.173134
51	1	0	3.946429	1.490369	-1.361438
52	8	0	3.121761	1.560063	-1.873405
53	1	0	2.501311	1.932843	-1.218734
54	1	0	0.835104	2.964259	0.207811
55	8	0	1.622229	2.462171	0.450445
56	1	0	1.276656	1.626957	0.920326
57	6	0	-0.767392	1.128796	-2.216768
58	6	0	-0.464659	2.389462	-2.724794
59	1	0	-2.361890	2.050475	0.628394
60	1	0	-1.860218	4.286999	-0.276375
61	1	0	-0.621584	4.517988	-2.424493
62	1	0	-0.483813	0.244445	-2.775389
63	1	0	0.078681	2.477408	-3.658864
64	1	0	1.207456	-3.320466	-0.345496
65	1	0	1.288395	-2.736532	1.321116
66	1	0	0.451637	-4.249970	0.956985
67	1	0	3.073120	3.165430	1.159617
68	8	0	3.958605	3.404437	1.523716
69	1	0	3.840266	3.411894	2.477877
70	1	0	5.762698	-2.274896	1.325989
71	8	0	4.974374	-2.823117	1.450224
72	1	0	4.776221	-3.088526	0.530558

SCF Done: E(RB+HF-LYP) = -1779.23815810 A.U. after 3 cycles
Convg = 0.1606D-08 -V/T = 2.0045

Zero-point correction= 0.602525 (a.u.)
Thermal correction to Energy= 0.654001
Thermal correction to Enthalpy= 0.654945
Thermal correction to Gibbs Free Energy= 0.513845
Sum of electronic and zero-point Energies= -1778.635633
Sum of electronic and thermal Energies= -1778.584157
Sum of electronic and thermal Enthalpies= -1778.583213
Sum of electronic and thermal Free Energies= -1778.724313

==

[10] para-Y-substituted N-ethylbenzamide + $OH^{-}(H_2O)_{16}$, Y = MeO and NO_2. precursor, TS1(am) and TS2(am)

(10-1) Y= MeO(CH3O), precursor
tuka02aaome.for.chk Stoichiometry C10H46NO19(1-)

Largest concise Abelian subgroup C1   NOp   1
Standard orientation:

Center Number	Atomic Number	Atomic Type	Coordinates (Angstroms) X	Y	Z
1	6	0	-2.121205	-1.104406	0.561960
2	6	0	-0.858666	-0.836162	1.316489
3	8	0	0.198429	-1.454504	1.008143
4	7	0	-0.906565	0.021432	2.346483
5	6	0	0.269557	0.407911	3.130060
6	6	0	-2.248104	-2.332274	-0.110292
7	6	0	-3.395233	-2.638525	-0.827170
8	6	0	-3.173580	-0.185155	0.480778
9	1	0	-1.727718	0.614137	2.467119
10	6	0	-0.136295	0.855323	4.532484
11	1	0	0.937957	-0.453527	3.180197
12	1	0	0.806351	1.208956	2.607015
13	8	0	-0.594346	1.825607	-0.552651
14	1	0	-0.605331	1.389401	-1.427901
15	1	0	0.373937	1.939839	-0.295468
16	8	0	1.956832	2.301001	0.073792
17	1	0	1.971109	2.910850	0.826735
18	1	0	2.005118	3.059483	-1.135009
19	8	0	1.934498	3.629905	-2.043114
20	1	0	0.978889	3.667837	-2.214814
21	1	0	2.207290	5.154184	-1.339487
22	8	0	2.270420	5.988585	-0.786614
23	1	0	3.114301	5.918002	-0.316293
24	1	0	2.225437	2.214550	-3.245660
25	8	0	2.051800	1.448420	-3.840387
26	1	0	2.663539	0.717080	-3.570213
27	1	0	0.587467	0.830694	-3.424762
28	8	0	-0.271728	0.420457	-3.081049
29	1	0	-0.886024	0.400455	-3.829257
30	1	0	0.868253	5.967973	0.296873
31	8	0	0.075713	5.994197	0.893560
32	1	0	-0.354788	6.840111	0.702322
33	1	0	0.604790	-1.730384	-0.894551
34	8	0	0.765543	-1.898588	-1.846936
35	1	0	0.264850	-1.192192	-2.314512

36	1	0	2.443601	-1.300192	-2.693732
37	8	0	3.243931	-0.909600	-3.103282
38	1	0	3.942571	-1.112848	-2.443743
39	1	0	1.951495	-3.498974	-1.674756
40	8	0	2.731992	-4.000252	-1.371301
41	1	0	2.433064	-4.559694	-0.619157
42	1	0	1.899366	-0.501092	0.854495
43	8	0	2.779854	-0.085258	0.773131
44	1	0	2.573679	0.869623	0.489714
45	1	0	3.868413	-1.018090	1.990280
46	8	0	4.301093	-1.857311	2.268127
47	1	0	4.693223	-2.157155	1.422944
48	1	0	3.793749	-1.199837	-0.289845
49	8	0	4.417210	-1.856387	-0.681074
50	1	0	3.879829	-2.664527	-0.867243
51	1	0	-1.461468	3.194538	0.073681
52	8	0	-1.883669	3.916115	0.610343
53	1	0	-1.187059	4.597619	0.737046
54	1	0	-2.371112	3.039357	1.975774
55	8	0	-2.683796	2.405947	2.687382
56	1	0	-3.560620	2.719673	2.950326
57	1	0	2.783950	-2.825873	2.528141
58	8	0	1.887829	-3.248968	2.553964
59	1	0	1.300729	-2.631290	2.072375
60	1	0	1.957535	-4.673945	1.590327
61	8	0	1.984657	-5.428645	0.938584
62	1	0	1.126889	-5.870758	1.010864
63	6	0	-4.327885	-0.478526	-0.246282
64	6	0	-4.442719	-1.710318	-0.900161
65	1	0	-1.430749	-3.043589	-0.067284
66	1	0	-3.497477	-3.586894	-1.345219
67	1	0	-3.096148	0.785570	0.958805
68	1	0	-5.116348	0.262836	-0.302714
69	8	0	-5.534090	-2.095642	-1.636651
70	1	0	-0.828031	1.702829	4.490303
71	1	0	-0.619028	0.037276	5.079587
72	1	0	0.754187	1.161742	5.092037
73	6	0	-6.612404	-1.180835	-1.767982
74	1	0	-7.057469	-0.943758	-0.792926
75	1	0	-6.291289	-0.251401	-2.255209
76	1	0	-7.353119	-1.681455	-2.393620

SCF Done: E(RB+HF-LYP) = -1893.08683444 A.U. after 1 cycles

Convg = 0.7549D-08 -V/T = 2.0093

Zero-point correction= 0.639390 (a.u.)

Thermal correction to Energy= 0.693874

Thermal correction to Enthalpy= 0.694819
Thermal correction to Gibbs Free Energy= 0.544924
Sum of electronic and zero-point Energies= -1892.447444
Sum of electronic and thermal Energies= -1892.392960
Sum of electronic and thermal Enthalpies= -1892.392016
Sum of electronic and thermal Free Energies= -1892.541911

(10-2) TS1(am), Y=MeO
tuka02aaome.chk Stoichiometry C10H46NO19(1-)
Standard orientation:

Center Number	Atomic Number	Atomic Type	Coordinates (Angstroms) X	Y	Z
1	6	0	2.172861	0.058594	0.234528
2	6	0	0.812803	0.124968	0.929476
3	8	0	0.091952	1.188496	0.696395
4	7	0	0.890573	-0.333708	2.246646
5	6	0	-0.283766	-0.323108	3.116851
6	6	0	2.787366	1.254191	-0.165048
7	6	0	4.042373	1.254738	-0.764537
8	6	0	2.862398	-1.138233	0.020446
9	1	0	1.441978	-1.182993	2.357910
10	6	0	0.126205	-0.595898	4.563394
11	1	0	-0.754516	0.658896	3.044809
12	1	0	-1.029223	-1.065950	2.793046
13	8	0	-0.059621	-1.213592	0.059029
14	1	0	0.056722	-1.025648	-0.893422
15	1	0	-1.673030	-1.386550	0.301184
16	8	0	-2.662542	-1.525711	0.477696
17	1	0	-2.710533	-2.366829	0.973108
18	1	0	-3.464060	-1.962792	-1.048497
19	8	0	-3.856420	-2.374867	-1.861085
20	1	0	-3.369959	-1.968071	-2.610926
21	1	0	-3.788129	-4.009488	-1.540388
22	8	0	-3.816458	-4.940410	-1.160711
23	1	0	-4.759877	-5.148773	-1.093634
24	8	0	-2.343846	-0.954929	-3.787750
25	1	0	-2.532241	0.017605	-3.543483
26	1	0	-2.535240	-1.025764	-4.734683
27	1	0	-0.602169	-0.956750	-3.276992
28	8	0	0.288856	-0.761069	-2.896859
29	1	0	0.862427	-1.508434	-3.120233
30	1	0	-2.978011	-4.680293	0.350975
31	8	0	-2.484939	-4.357026	1.153269
32	1	0	-2.747172	-4.922112	1.894214

33	1	0	-0.033074	1.684112	-0.976854
34	8	0	-0.061275	1.956127	-1.937384
35	1	0	0.408515	1.236411	-2.399159
36	1	0	-1.661753	1.743254	-2.783386
37	8	0	-2.541017	1.568907	-3.199017
38	1	0	-3.165608	1.975519	-2.541827
39	1	0	-0.773072	3.814799	-1.790150
40	8	0	-1.377273	4.535344	-1.525749
41	1	0	-0.956204	4.962762	-0.744509
42	1	0	-1.637181	1.193209	0.884483
43	8	0	-2.629958	1.224922	0.976383
44	1	0	-2.899600	0.280368	0.934554
45	1	0	-2.845359	2.301293	2.355945
46	8	0	-2.744178	3.014261	3.037903
47	1	0	-3.226191	3.773022	2.675654
48	1	0	-3.319061	2.306428	-0.382586
49	8	0	-3.643969	2.912318	-1.086252
50	1	0	-2.942234	3.601917	-1.171187
51	1	0	0.196318	-2.862525	0.383005
52	8	0	0.333404	-3.814204	0.667140
53	1	0	-0.570584	-4.150450	0.830235
54	1	0	1.237201	-3.605389	2.132123
55	8	0	1.788653	-3.330240	2.914233
56	1	0	2.657108	-3.732609	2.771380
57	1	0	-0.908819	3.332806	2.731217
58	8	0	-0.006130	3.444174	2.354913
59	1	0	0.138695	2.633822	1.807579
60	1	0	-0.074751	4.820476	1.371096
61	8	0	-0.155805	5.611678	0.763531
62	1	0	0.744954	5.946737	0.648835
63	6	0	4.125371	-1.154874	-0.579728
64	6	0	4.718378	0.048198	-0.974282
65	1	0	2.265107	2.191047	-0.005005
66	1	0	4.514546	2.181434	-1.076904
67	1	0	2.406593	-2.079613	0.306795
68	1	0	4.624286	-2.105130	-0.735279
69	8	0	5.955644	0.144245	-1.575842
70	1	0	0.622144	-1.568853	4.652187
71	1	0	0.814829	0.178603	4.918934
72	1	0	-0.757378	-0.597768	5.212832
73	6	0	6.677161	-1.053499	-1.803470
74	1	0	6.896062	-1.577383	-0.863246
75	1	0	6.131742	-1.731750	-2.473489
76	1	0	7.614259	-0.754492	-2.277311

SCF Done: E(RB+HF-LYP) = -1893.05649779 A.U. after 1 cycles

Convg = 0.5166D-08 -V/T = 2.0093
Zero-point correction= 0.640600 (a.u.)
Thermal correction to Energy= 0.693667
Thermal correction to Enthalpy= 0.694611
Thermal correction to Gibbs Free Energy= 0.551135
Sum of electronic and zero-point Energies= -1892.415898
Sum of electronic and thermal Energies= -1892.362831
Sum of electronic and thermal Enthalpies= -1892.361887
Sum of electronic and thermal Free Energies= -1892.505363

(10-3) TS2(am), Y=MeO
tuka02bzome.chk Stoichiometry C10H46NO19(1-)

Standard orientation:

Center Number	Atomic Number	Atomic Type	Coordinates (Angstroms) X	Y	Z
1	6	0	-1.397385	0.904052	-0.604076
2	6	0	-1.803341	-0.572130	-0.470918
3	8	0	-2.601009	-0.845862	0.560041
4	7	0	-0.482102	-1.399609	-0.312873
5	6	0	-0.714152	-2.874418	-0.237084
6	6	0	-1.747948	1.842297	0.375732
7	6	0	-1.375411	3.177837	0.251619
8	6	0	-0.627349	3.598593	-0.855539
9	1	0	0.194294	-1.189325	-1.065292
10	1	0	-1.358156	-3.041969	0.627935
11	1	0	-1.269354	-3.154679	-1.133666
12	6	0	0.586761	-3.662176	-0.120593
13	8	0	-2.295874	-1.054548	-1.725308
14	1	0	-3.185170	-0.656743	-1.852954
15	1	0	-5.215271	-2.952568	-0.871838
16	8	0	-5.533779	-2.929595	-1.821782
17	1	0	-4.858969	-3.410962	-2.323763
18	1	0	-0.005909	-1.055216	0.578291
19	8	0	0.842890	-0.513251	1.916253
20	1	0	0.185315	-0.472247	2.634968
21	1	0	2.369367	-1.281169	1.627565
22	8	0	3.177492	-1.402067	1.059480
23	1	0	3.592739	-2.289187	1.151878
24	1	0	-5.336154	-1.210129	-2.066358
25	8	0	-5.046694	-0.257901	-2.048659
26	1	0	-5.334590	0.139307	-2.883421
27	1	0	-4.147067	-0.054429	0.739275
28	8	0	-5.101473	0.157840	0.917126

29	1	0	-5.445819	0.366424	0.030613
30	1	0	2.509875	-1.212165	-0.607819
31	8	0	2.233777	-1.011017	-1.535373
32	1	0	2.420756	-0.035091	-1.583586
33	1	0	4.332422	-0.089548	0.886273
34	8	0	5.020612	0.585727	0.659180
35	1	0	4.896414	1.287251	1.347518
36	1	0	-3.747260	-2.196696	0.568665
37	8	0	-4.598657	-2.695630	0.700340
38	1	0	-5.173787	-1.983092	1.043882
39	1	0	6.192343	-0.502188	0.001599
40	8	0	6.626953	-1.286876	-0.429827
41	1	0	7.517096	-1.001035	-0.680478
42	1	0	3.760928	-1.998921	-2.096879
43	8	0	4.573521	-2.556457	-2.094880
44	1	0	5.293110	-1.967659	-1.791142
45	1	0	-2.213372	-1.359863	3.588360
46	8	0	-1.912082	-0.550360	3.149166
47	1	0	-2.126232	-0.678973	2.180408
48	1	0	-3.437144	0.719799	3.523370
49	8	0	-4.269583	1.232089	3.552200
50	1	0	-4.725788	1.005585	2.718831
51	1	0	3.813782	1.422389	-0.730520
52	8	0	2.922552	1.613553	-1.087191
53	1	0	2.382801	1.706975	-0.250465
54	1	0	0.986301	2.343897	1.358152
55	8	0	1.714887	1.704341	1.374334
56	1	0	1.221333	0.579346	1.690672
57	6	0	-0.674625	1.345314	-1.716871
58	6	0	-0.280800	2.676830	-1.849442
59	1	0	-2.315382	1.530253	1.244844
60	1	0	-1.649715	3.905526	1.009435
61	8	0	-0.284704	4.927151	-0.886056
62	1	0	-0.409449	0.641310	-2.500478
63	1	0	0.305822	2.973465	-2.710276
64	1	0	1.238781	-3.495270	-0.983472
65	1	0	1.140504	-3.395342	0.784238
66	1	0	0.353878	-4.731871	-0.071693
67	1	0	3.083132	2.202230	2.105864
68	8	0	4.011861	2.391658	2.472080
69	1	0	3.983732	2.099576	3.394759
70	1	0	5.668998	-3.032670	0.605462
71	8	0	4.845301	-3.547525	0.528642
72	1	0	4.631193	-3.436063	-0.429066
73	6	0	0.679305	5.341403	-1.846791
74	1	0	1.595821	4.744561	-1.768361

75	1	0	0.283322	5.272648	-2.868872
76	1	0	0.896998	6.386036	-1.616685

SCF Done: E(RB+HF-LYP) = -1893.04834908 A.U. after 1 cycles
Convg = 0.3567D-08 -V/T = 2.0093
Zero-point correction= 0.638580 (a.u.)
Thermal correction to Energy= 0.691004
Thermal correction to Enthalpy= 0.691948
Thermal correction to Gibbs Free Energy= 0.548583
Sum of electronic and zero-point Energies= -1892.409769
Sum of electronic and thermal Energies= -1892.357345
Sum of electronic and thermal Enthalpies= -1892.356401
Sum of electronic and thermal Free Energies= -1892.499766

(10-4) precursor, Y=NO2
tuka02aano2.for.chk Stoichiometry C9H43N2O20(1-)
Standard orientation:

Center Number	Atomic Number	Atomic Type	Coordinates (Angstroms) X	Y	Z
1	6	0	2.212908	-0.020542	0.713925
2	6	0	0.917334	0.306723	1.408306
3	8	0	0.280904	1.336460	1.064696
4	7	0	0.534228	-0.479950	2.419459
5	6	0	-0.712623	-0.273779	3.163853
6	6	0	2.909068	1.038319	0.111856
7	6	0	4.121385	0.817040	-0.532495
8	6	0	2.736304	-1.321147	0.646108
9	1	0	0.980568	-1.389207	2.552533
10	6	0	-0.615780	-0.879567	4.561686
11	1	0	-0.895826	0.800986	3.220731
12	1	0	-1.542485	-0.718355	2.601157
13	8	0	-0.395642	-1.834101	-0.503284
14	1	0	-0.200809	-1.480738	-1.394625
15	1	0	-1.334789	-1.540270	-0.274459
16	8	0	-2.924274	-1.179194	0.008993
17	1	0	-3.262842	-1.715951	0.741256
18	1	0	-3.282388	-1.823078	-1.230109
19	8	0	-3.464434	-2.347640	-2.143184
20	1	0	-2.630700	-2.815516	-2.315096
21	1	0	-4.407591	-3.611465	-1.493162
22	8	0	-4.826067	-4.370878	-0.991079
23	1	0	-5.598046	-3.993229	-0.544586
24	1	0	-3.056305	-0.940108	-3.326297

25	8	0	-2.525446	-0.343817	-3.902945
26	1	0	-2.738894	0.588980	-3.644827
27	1	0	-0.964126	-0.479204	-3.437347
28	8	0	-0.022271	-0.516443	-3.064848
29	1	0	0.549735	-0.802081	-3.792417
30	1	0	-3.580467	-5.037624	0.081210
31	8	0	-2.867240	-5.459764	0.625679
32	1	0	-2.791150	-6.361416	0.280528
33	1	0	0.110887	1.774979	-0.884830
34	8	0	0.118365	2.014814	-1.833534
35	1	0	0.228718	1.159626	-2.309122
36	1	0	-1.636588	2.288643	-2.753461
37	8	0	-2.520261	2.298953	-3.175415
38	1	0	-3.064649	2.790969	-2.521838
39	1	0	-0.219229	3.990133	-1.637095
40	8	0	-0.711675	4.778026	-1.339868
41	1	0	-0.218423	5.151263	-0.575260
42	1	0	-1.666333	1.289717	0.839749
43	8	0	-2.634455	1.320178	0.717286
44	1	0	-2.869245	0.374248	0.422292
45	1	0	-3.223505	2.651107	1.908281
46	8	0	-3.225271	3.592897	2.193459
47	1	0	-3.409042	4.048884	1.347520
48	1	0	-2.982400	2.775072	-0.363027
49	8	0	-3.216809	3.643529	-0.766480
50	1	0	-2.361550	4.116986	-0.910460
51	1	0	-0.295412	-3.483622	0.052127
52	8	0	-0.242817	-4.331327	0.566987
53	1	0	-1.162935	-4.673327	0.624574
54	1	0	0.504273	-3.799593	1.975477
55	8	0	1.031507	-3.372082	2.715910
56	1	0	1.622224	-4.060026	3.054036
57	1	0	-1.454281	3.757186	2.526007
58	8	0	-0.465064	3.721719	2.592956
59	1	0	-0.207372	2.912740	2.108714
60	1	0	0.153072	5.037815	1.664442
61	8	0	0.479111	5.727902	1.023574
62	1	0	1.426559	5.827758	1.192293
63	6	0	3.944767	-1.557972	-0.001654
64	6	0	4.625430	-0.483303	-0.575786
65	1	0	2.486564	2.035645	0.148151
66	1	0	4.670088	1.627022	-0.996530
67	1	0	2.198116	-2.157935	1.077495
68	1	0	4.356615	-2.557105	-0.070600
69	7	0	5.909405	-0.728589	-1.247297
70	1	0	-0.408251	-1.953213	4.512387

71	1	0	0.177207	-0.396406	5.143901
72	1	0	-1.564007	-0.733438	5.089800
73	8	0	6.340082	-1.885082	-1.272623
74	8	0	6.495113	0.234387	-1.749959

SCF Done: E(RB+HF-LYP) = -1983.07780819 A.U. after 1 cycles
Convg = 0.3822D-08 -V/T = 2.0093
S**2 = 0.0000
Zero-point correction= 0.609038(a.u.)
Thermal correction to Energy= 0.663442
Thermal correction to Enthalpy= 0.664386
Thermal correction to Gibbs Free Energy= 0.514915
Sum of electronic and zero-point Energies= -1982.468770
Sum of electronic and thermal Energies= -1982.414366
Sum of electronic and thermal Enthalpies= -1982.413422
Sum of electronic and thermal Free Energies= -1982.562893

(10-5) TS1(am), Y=NO2
tuka02aano2.chk Stoichiometry C9H43N2O20(1-)

Standard orientation:

Center Number	Atomic Number	Atomic Type	Coordinates (Angstroms) X	Y	Z
1	6	0	2.003506	-0.382541	0.325705
2	6	0	0.596931	-0.193648	0.909974
3	8	0	0.070439	0.989661	0.770460
4	7	0	0.509247	-0.805230	2.167612
5	6	0	-0.694690	-0.672563	2.984689
6	6	0	2.767614	0.754171	0.032045
7	6	0	4.068817	0.638487	-0.448776
8	6	0	2.568593	-1.652578	0.124662
9	1	0	0.898806	-1.745621	2.205278
10	6	0	-0.416350	-1.124921	4.417407
11	1	0	-0.999270	0.375196	2.977277
12	1	0	-1.531640	-1.257010	2.568429
13	8	0	-0.329607	-1.268323	-0.163109
14	1	0	-0.151828	-0.987166	-1.085665
15	1	0	-1.930816	-1.144181	-0.005323
16	8	0	-2.949570	-1.146586	0.027441
17	1	0	-3.189496	-1.898198	0.606302
18	1	0	-3.342257	-1.707211	-1.497842
19	8	0	-3.565829	-2.137946	-2.378265
20	1	0	-2.740546	-2.562874	-2.663358

21	1	0	-4.607579	-3.428673	-1.796403
22	8	0	-5.082680	-4.123620	-1.266840
23	1	0	-6.011817	-3.850657	-1.249148
24	1	0	-3.118304	-0.479956	-3.472258
25	8	0	-2.477799	0.054683	-3.978478
26	1	0	-2.594543	1.006748	-3.699132
27	1	0	-0.931554	-0.264463	-3.399787
28	8	0	-0.030234	-0.407570	-2.978972
29	1	0	0.516582	-0.852129	-3.643222
30	1	0	-4.147658	-4.039452	0.234317
31	8	0	-3.503515	-3.797407	0.949318
32	1	0	-3.775627	-4.261579	1.754389
33	1	0	0.103519	1.782480	-0.852436
34	8	0	0.181671	2.167570	-1.762875
35	1	0	0.375457	1.386371	-2.320615
36	1	0	-1.439766	2.538668	-2.721959
37	8	0	-2.314482	2.614358	-3.162820
38	1	0	-2.855636	3.062768	-2.467751
39	1	0	-0.141590	4.105151	-1.359424
40	8	0	-0.586903	4.894459	-0.996021
41	1	0	-0.113969	5.112318	-0.160411
42	1	0	-1.637798	1.313400	0.807197
43	8	0	-2.610187	1.525413	0.846978
44	1	0	-3.036134	0.670705	0.623256
45	1	0	-2.766730	2.487882	2.333226
46	8	0	-2.597779	3.116427	3.078388
47	1	0	-2.883804	3.978905	2.740159
48	1	0	-3.015318	3.021419	-0.250561
49	8	0	-3.175950	3.812212	-0.808993
50	1	0	-2.325067	4.313550	-0.791848
51	1	0	-0.503920	-2.991522	0.000438
52	8	0	-0.671717	-3.954771	0.196756
53	1	0	-1.615733	-4.005354	0.445772
54	1	0	0.302514	-4.085958	1.643634
55	8	0	0.878957	-3.942784	2.440170
56	1	0	1.574185	-4.614490	2.393433
57	1	0	-0.718467	3.074966	2.978792
58	8	0	0.228983	3.019308	2.716871
59	1	0	0.268817	2.272816	2.072801
60	1	0	0.587813	4.488026	1.934424
61	8	0	0.730664	5.346195	1.443065
62	1	0	1.687829	5.487874	1.425320
63	6	0	3.867200	-1.788960	-0.350757
64	6	0	4.606752	-0.636364	-0.631667
65	1	0	2.328446	1.733388	0.180273
66	1	0	4.664400	1.513608	-0.678016

67	1	0	1.985117	-2.540782	0.337578
68	1	0	4.310836	-2.764439	-0.507828
69	7	0	5.976647	-0.771853	-1.128211
70	1	0	-0.084840	-2.169225	4.441419
71	1	0	0.363143	-0.502944	4.870182
72	1	0	-1.325103	-1.037697	5.024636
73	8	0	6.431629	-1.911096	-1.286579
74	8	0	6.618638	0.257120	-1.365377

SCF Done: E(RB+HF-LYP) = -1983.04975966 A.U. after 1 cycles

Convg = 0.4205D-08 -V/T = 2.0092

S**2 = 0.0000

Zero-point correction= 0.610950 (a.u.)

Thermal correction to Energy= 0.664146

Thermal correction to Enthalpy= 0.665090

Thermal correction to Gibbs Free Energy= 0.519627

Sum of electronic and zero-point Energies= -1982.438810

Sum of electronic and thermal Energies= -1982.385614

Sum of electronic and thermal Enthalpies= -1982.384669

Sum of electronic and thermal Free Energies= -1982.530133

(10-6) TS2(am), Y=NO2

tuka02bzno2.chk Stoichiometry C9H43N2O20(1-)

Standard orientation:

Center Number	Atomic Number	Atomic Type	Coordinates (Angstroms) X	Y	Z
1	6	0	-1.374096	0.701285	-0.568993
2	6	0	-1.660668	-0.813886	-0.448966
3	8	0	-2.444876	-1.148457	0.571402
4	7	0	-0.290762	-1.540934	-0.293374
5	6	0	-0.431687	-3.030150	-0.236717
6	6	0	-1.831543	1.589505	0.412093
7	6	0	-1.643488	2.963112	0.270112
8	6	0	-0.985472	3.437181	-0.865226
9	1	0	0.382446	-1.281021	-1.037491
10	1	0	-1.041056	-3.250115	0.641344
11	1	0	-0.991340	-3.328439	-1.124476
12	6	0	0.915759	-3.741006	-0.166367
13	8	0	-2.114684	-1.293362	-1.714211
14	1	0	-3.057095	-1.024925	-1.806088
15	1	0	-4.944193	-3.479657	-0.795196
16	8	0	-5.316589	-3.470844	-1.724377
17	1	0	-4.699339	-3.995800	-2.255293

18	1	0	0.156441	-1.181639	0.609113
19	8	0	0.956969	-0.617156	1.954490
20	1	0	0.311533	-0.655044	2.683969
21	1	0	2.543655	-1.282626	1.609646
22	8	0	3.334032	-1.337066	1.010575
23	1	0	3.820162	-2.190704	1.078355
24	1	0	-5.163186	-1.753275	-2.003597
25	8	0	-4.916197	-0.789425	-1.986392
26	1	0	-5.224908	-0.405386	-2.820122
27	1	0	-4.055888	-0.504299	0.774341
28	8	0	-5.016519	-0.375520	0.990299
29	1	0	-5.411918	-0.190223	0.120418
30	1	0	2.625995	-1.165680	-0.633031
31	8	0	2.295278	-0.950086	-1.539844
32	1	0	2.406822	0.040746	-1.549852
33	1	0	4.402271	0.048803	0.799000
34	8	0	5.037392	0.769017	0.559448
35	1	0	4.922154	1.427338	1.290423
36	1	0	-3.480034	-2.609892	0.600579
37	8	0	-4.284817	-3.171255	0.755589
38	1	0	-4.905724	-2.504784	1.112230
39	1	0	6.260191	-0.231398	-0.137814
40	8	0	6.733948	-0.983019	-0.587597
41	1	0	7.595073	-0.636754	-0.862515
42	1	0	3.875413	-1.832963	-2.179818
43	8	0	4.713138	-2.349430	-2.198157
44	1	0	5.411805	-1.725692	-1.914935
45	1	0	-2.064821	-1.708956	3.612514
46	8	0	-1.785686	-0.888315	3.179543
47	1	0	-1.987939	-1.014204	2.209637
48	1	0	-3.278227	0.421373	3.487604
49	8	0	-4.060150	1.005299	3.428060
50	1	0	-4.578837	0.650459	2.680520
51	1	0	3.688071	1.580625	-0.722635
52	8	0	2.761963	1.686186	-1.020824
53	1	0	2.269453	1.750435	-0.154325
54	1	0	0.969725	2.301528	1.625861
55	8	0	1.694062	1.669078	1.507769
56	1	0	1.262685	0.502237	1.782022
57	6	0	-0.709519	1.207858	-1.695887
58	6	0	-0.503554	2.574440	-1.851015
59	1	0	-2.354742	1.217885	1.284838
60	1	0	-2.009752	3.656521	1.017447
61	7	0	-0.809552	4.884515	-1.036683
62	1	0	-0.360234	0.529266	-2.467124
63	1	0	0.022661	2.971734	-2.709390

64	1	0	1.532004	-3.528502	-1.045215
65	1	0	1.479222	-3.454075	0.725958
66	1	0	0.744671	-4.822483	-0.124727
67	1	0	3.096944	2.174387	2.198699
68	8	0	4.030077	2.391326	2.529826
69	1	0	4.065985	2.047686	3.434202
70	1	0	5.919154	-2.788761	0.461685
71	8	0	5.122593	-3.347296	0.404418
72	1	0	4.875286	-3.241522	-0.545865
73	8	0	-0.354410	5.292478	-2.109257
74	8	0	-1.133369	5.624433	-0.102598

SCF Done: E(RB+HF-LYP) = -1983.04011267 A.U. after 1 cycles
Convg = 0.6356D-08 -V/T = 2.0092
S**2 = 0.0000
Zero-point correction= 0.607971 (a.u.)
Thermal correction to Energy= 0.660482
Thermal correction to Enthalpy= 0.661426
Thermal correction to Gibbs Free Energy= 0.516834
Sum of electronic and zero-point Energies= -1982.432141
Sum of electronic and thermal Energies= -1982.379631
Sum of electronic and thermal Enthalpies= -1982.378686
Sum of electronic and thermal Free Energies= -1982.523279
